# Meiosis-specific gene discovery in plants: RNA-Seq applied to isolated *Arabidopsis *male meiocytes

**DOI:** 10.1186/1471-2229-10-280

**Published:** 2010-12-17

**Authors:** Changbin Chen, Andrew D Farmer, Raymond J Langley, Joann Mudge, John A Crow, Gregory D May, James Huntley, Alan G Smith, Ernest F Retzel

**Affiliations:** 1 Department of Horticultural Science, University of Minnesota, 1970 Folwell Avenue, St. Paul, MN 55108, USA; 2 National Center for Genome Resources, 2935 Rodeo Park Drive E., Santa Fe, NM 87505, USA; 3 Illumina Inc., Hayward, California 94545, USA; 4 Immunology, Lovelace Respiratory Research Institute, 2425 Ridgecrest Drive SE, Albuquerque, NM 87108, USA

## Abstract

**Background:**

Meiosis is a critical process in the reproduction and life cycle of flowering plants in which homologous chromosomes pair, synapse, recombine and segregate. Understanding meiosis will not only advance our knowledge of the mechanisms of genetic recombination, but also has substantial applications in crop improvement. Despite the tremendous progress in the past decade in other model organisms (e.g., *Saccharomyces cerevisiae *and *Drosophila melanogaster*), the global identification of meiotic genes in flowering plants has remained a challenge due to the lack of efficient methods to collect pure meiocytes for analyzing the temporal and spatial gene expression patterns during meiosis, and for the sensitive identification and quantitation of novel genes.

**Results:**

A high-throughput approach to identify meiosis-specific genes by combining isolated meiocytes, RNA-Seq, bioinformatic and statistical analysis pipelines was developed. By analyzing the studied genes that have a meiosis function, a pipeline for identifying meiosis-specific genes has been defined. More than 1,000 genes that are specifically or preferentially expressed in meiocytes have been identified as candidate meiosis-specific genes. A group of 55 genes that have mitochondrial genome origins and a significant number of transposable element (TE) genes (1,036) were also found to have up-regulated expression levels in meiocytes.

**Conclusion:**

These findings advance our understanding of meiotic genes, gene expression and regulation, especially the transcript profiles of MGI genes and TE genes, and provide a framework for functional analysis of genes in meiosis.

## Background

Despite more than a century of research, the mechanisms of meiosis in flowering plants remain largely unknown with respect to the regulation and progression of homologous chromosome pairing, synapse, recombination, and segregation [1-3]. Until the late 1990s, yeast was the primary model system for investigating the molecular mechanisms of meiosis [4], while flowering plants were only sparingly explored with the exception of cytological studies [5,6]. In the past decade, however, flowering plants have become model systems to unravel the principles of meiosis in multicellular organisms [6-8]. Genetic resources from model plants such as *Arabidopsis *and rice have been significantly enhanced since the year 2000 as genome sequences were completed and genome-wide T-DNA insertion mutants became available [9-12].

**Figure 1:**
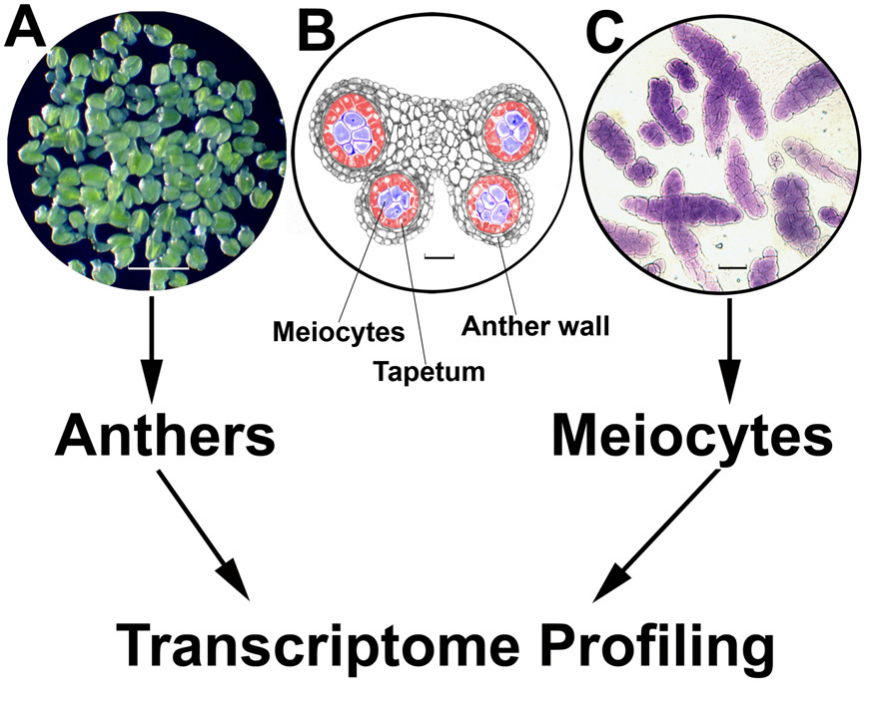
***Arabidopsis *male meiocytes and anthers**. **A**. A collection of anthers that undergo meiosis. In the buffer, the anther lobes should be clear and transparent (anther stages 5-7), otherwise it has passed meiosis and at free microscope stage (anther stages 8 or later). **B**. A thin section of a stage 6 anther. Male meiocytes were developing in the centers of four anther lobes (blue) with thick callose walls, and surrounded by tapetum (red) and the other anther wall cells (gray). **C**. A collection of male meiocytes. Male meiocytes clustered together in anther lobes in meiosis I and develop clear and thick callose-wall through late meiosis I and meiosis II, which are the indicators for distinguishing male meiocytes from somatic cells. Bars, 500 μm (A), 20 μm (B and C).

Compared to the functional genomic studies on pollen/gametophyte, in which significant progress has been made [13-15], using flowering plants to study meiosis has some inherent methodological challenges, especially the relatively small physical size of anthers that undergo meiosis in plants, and the small size is particularly the case in *Arabidopsis *[5]. Although the male meiocytes (pollen mother cells) are highly synchronized in anther lobes, each anther contains only a small fraction of male meiocytes. For instance, male meiocytes constitute about 1% of *Arabidopsis *anther tissues, making the isolation of meiocytes challenging (Figure 1B). To date, several methods have been developed to concentrate meiocytes for transcriptome or proteome profiling. One approach was developed for collecting meiocytes from *Brassica *that have larger anthers [5]. Subsequent meiotic proteomics analysis, however, could not directly characterize gene functions in meiosis due to the limited genetic resources in *Brassica*, i.e., a characterized mutant collection, although a number of genes with potential functions in meiosis were identified [5]. Other researchers have collected anthers that are undergoing meiosis in several species, such as *Arabidopsis *[16], rice [17,18], maize [19] and wheat [20] for transcriptomic studies, since anthers are much easier to obtain compared to meiocytes. As mentioned above, this approach is inefficient for the exploration of meiosis, as only a small portion of cells in anthers are meiocytes (Figure 1B). Genes identified through this approach also included genes that are specific for anther wall development (Figure 1B) [16].

We hypothesize that the meiosis-specific genes can be identified by comparing transcriptome profiles of meiocytes and anthers with seedling controls. A number of techniques are available for sampling specific plant cells, including fluorescence-activated cell sorting (FACS) [21] and laser capture microdissection (LCM) [22,23]. LCM has been successfully applied in specific plant cell sampling and transcriptome analyses [24-28], such as transcriptional profiling during *Arabidopsis *embryogenesis [29,30]. Although FACS and LCM have been very useful in many areas of plant transcriptome analyses, the potential biases, such as the enzyme digestion in FACS and mRNA amplification in LCM make these methods less appealing to researchers who perform transcriptome profiling of key biological processes such as meiosis [22]. The lack of efficient methods to isolate meiocytes largely accounts for the lack of progress in global identifications of plant meiotic genes. At present, only 68 meiotic genes have been identified in *Arabidopsis*, largely through mutagenesis and phylogenetic experiments [6-8,31] (Additional file 1, Table S1). By way of comparison, 915 core-meiotic genes have been found in yeast through microarray analysis, and more than 300 have been studied at the molecular level [4].

In this paper, we describe the application of a newly developed method to effectively collect male meiocytes, which enables the collection of sufficient total RNA for transcriptome studies from highly condensed meiocytes without mRNA amplification. From the total RNA collected using this method, we have applied RNA-Seq technology and bioinformatic analysis to identify meiosis-specific genes in *Arabidopsis*. As a result, 55 genes on pericentromeric region of chromosome II that covers a large mitochondrial genomic insertion (MGI) and 1,036 transposable element (TE) genes were discovered to be preferentially or specifically expressed in meiocytes.

## Results

### Transcriptome sequencing

**Figure 2:**
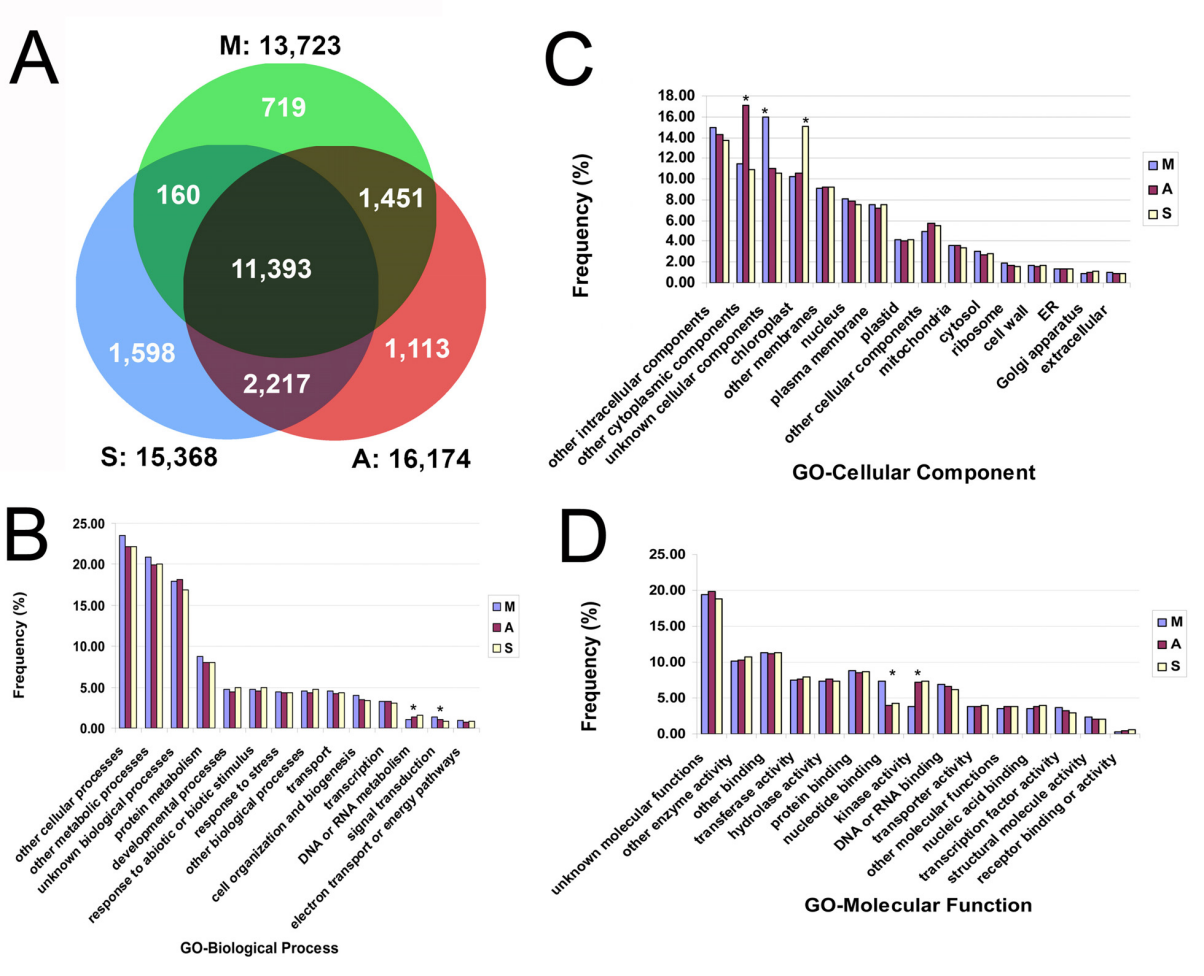
**Distribution of expressed mRNAs among gene function categories**. **A**. Venn diagram of overall gene expression in meiocytes, anthers, and seedlings. **B**. GO-Biological Process: Distribution and comparison of expressed and annotated genes in meiocytes, anthers and seedlings, the significantly over- or underrepresented gene populations in DNA or RNA metabolism and signal transduction are marked with stars (*). **C**. GO-Cellular Components: Distribution and comparison of expressed and annotated genes in meiocytes, anthers and seedlings, the significantly over- or underrepresented genes among tissues are marked with stars (*) in unknown cellular components, other cytoplasmic components, and chloroplast. **D**. GO-Molecular Function: Distribution and comparison of expressed and annotated genes in meiocytes, anthers and seedlings, the significantly over- or underrepresented gene populations in nucleotide binding and kinase activity are marked with stars (*). S = seedling, A = anther, M = meiocyte.

The sequencing of meiocyte, anther and seedling transcriptomes generated average genome and transcriptome coverage over 10×. Technical replicates were highly reproducible (Additional file 2, Figure S1 and Additional file 3, Figure S2). The percentage of reads aligned to the genome was an average of 79%, 75% and 81% for meiocyte, anther and seedling libraries, respectively. The percentage of reads aligned is a function both of the quality of the libraries and the relative completeness of the *Arabidopsis *genome. Through the comparative analysis of sequencing datasets against the TAIR 9 reference data, at the cutoff point of five reads per million reads, a total of 13,723 genes were expressed in meiocytes, with 15,368 and 16,174 genes detected in seedling and anther controls, respectively (Figure 2A). At a cutoff of one read per million reads, however, 23,843, 19,930, and 21,473 genes were expressed in meiocytes, seedlings and anthers, respectively. Together, the results suggest a significantly large population of genes are expressed in meiocytes at low levels.

Gene ontology (GO) analysis revealed groups of functionally-related, annotated genes expressed in meiocytes (Figure S3). A comparative GO analysis among previously annotated nuclear genes that were detected in meiocytes, anthers and seedlings revealed distinct gene expression profiles among the different tissues. Notably, by cellular components, a significantly increased number of functionally unknown cellular component genes were expressed in meiocytes, while an increased percentage of other cytoplasmic component genes were expressed in anthers and chloroplast genes in seedlings (Figure 2C). In addition, when genes expressed in meiocytes were partitioned by biological process, a smaller number of genes function in DNA or RNA metabolism, and a larger number of signal transduction genes were observed, which suggested a lower level of DNA or RNA metabolic activity and a higher level of signal transduction occurs in meiosis (Figure 2B). By molecular function, meiocytes demonstrated significantly higher activities of nucleotide binding and lower levels of kinase activities when compared to anthers and seedlings (Figure 2D).

### Statistical analysis

**Figure 3:**
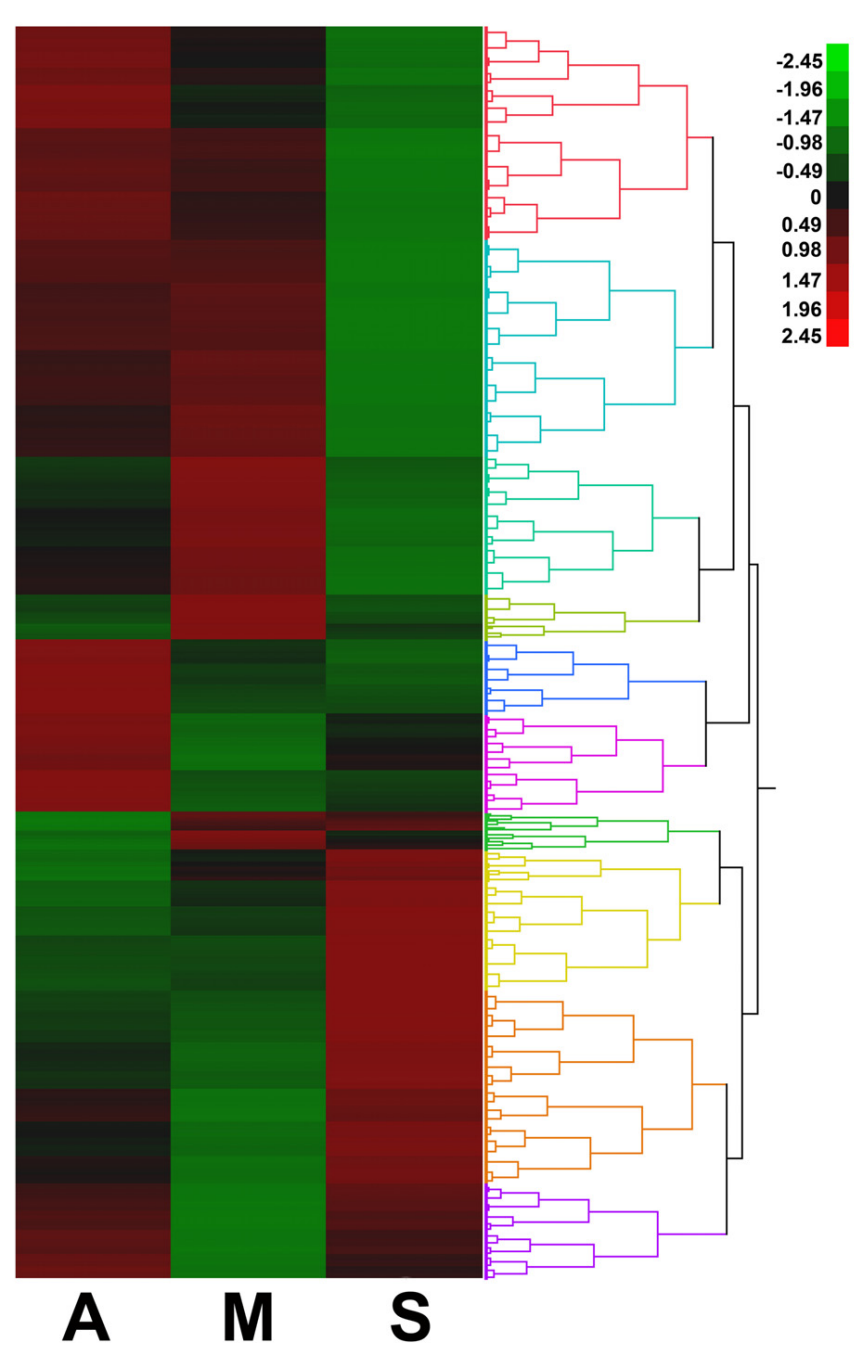
**Heatmap of differential expression between all pairwise comparisons using ANOVA analysis**. S = seedling, A = anther, M = meiocyte.

**Figure 4:**
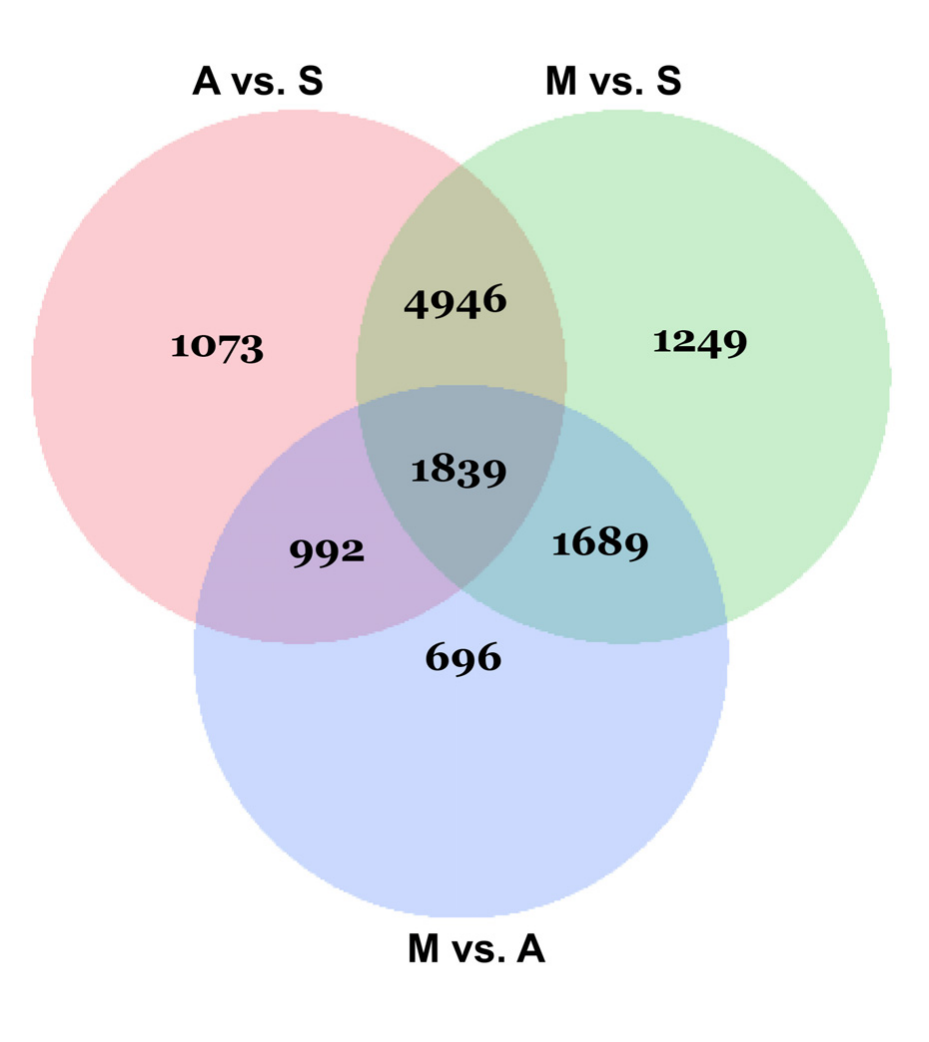
**Venn diagram of uniquely expressed genes, and at least twofold or greater changes between all pairwise comparisons**. S = seedling, A = anther, M = meiocyte.

ANOVA analysis of all pair-wise differences between seedling (S), anther control (A) and anther meiocytes (M) was determined as described in the Materials and Methods. A total of 16,088 differences were noted in the three comparisons (S vs A = 12,871, S vs M = 13,385, A vs M = 10,157). The heat map analysis suggested a number of the changes were unique to anthers or meiocytes (Figure 3). Because of the high similarity in technical replicates (Additional file 2, Figure S1, and Additional file 3, Figure S2), a second filter of at least a twofold or greater expression difference in the pair-wise comparisons was performed to reduce the total number of significant changes. This reduced the total number of significantly expressed genes to 12,484 (Figure 4; S vs A = 8,850, S vs M = 9,723, A vs M = 5,216). To find unique meiosis genes that were only differentially expressed within meiocytes compared to anthers, we performed Venn Diagrams (Figure 4) of the significantly expressed genes after ANOVA and the twofold filter. There were 696 genes that were uniquely expressed in a comparison of meiocytes anthers, among which 607 genes were preferentially expressed in meiocytes.

### Transcript profiling of studied meiotic genes

In *Arabidopsis*, a total of 68 genes have been reported to date with functions in meiosis (Additional file 1, Table S1). Among them, *MS5*, *AtSRP2*, and *AtSRP3 *were not detected in seedlings [32,33]; and *MND1*, *AtSPO11-2*, *AtSRP2 *and *MS5 *were expressed at twofold or greater levels in meiocytes than anthers. However, the expression levels of *AtSRP2 *and *MND1 *were very low in meiocytes, at 0.7 reads per million reads [31-38]. 31 genes were expressed at twofold or greater in meiocytes than in seedlings and 49 genes were expressed at twofold or greater in anthers than in seedlings. 29 of 68 genes were preferentially expressed in both meiocytes and anthers compared to seedlings, which include key meiotic recombination genes, such as *AtSPO11-1*, *AtDMC1*, *AtRAD51C*, *AtXRCC3*, *AtMSH4*, *AtMSH5*, *AtMER3/RCK*, *PTD*, *AtMUS81*, and *SDS *[39-55]. 17 genes didn't show significantly differential expression in all explored tissues, which included genes that were not meiosis-specific, or may function in both meiosis and mitosis, i.e. *AML2*, *AML3*, *AML5*, *ASK2*, *ATK5 *[56-58].

### Mitochondrial genomic insertion genes on chromosome II are preferentially expressed in meiocytes

**Figure 5:**
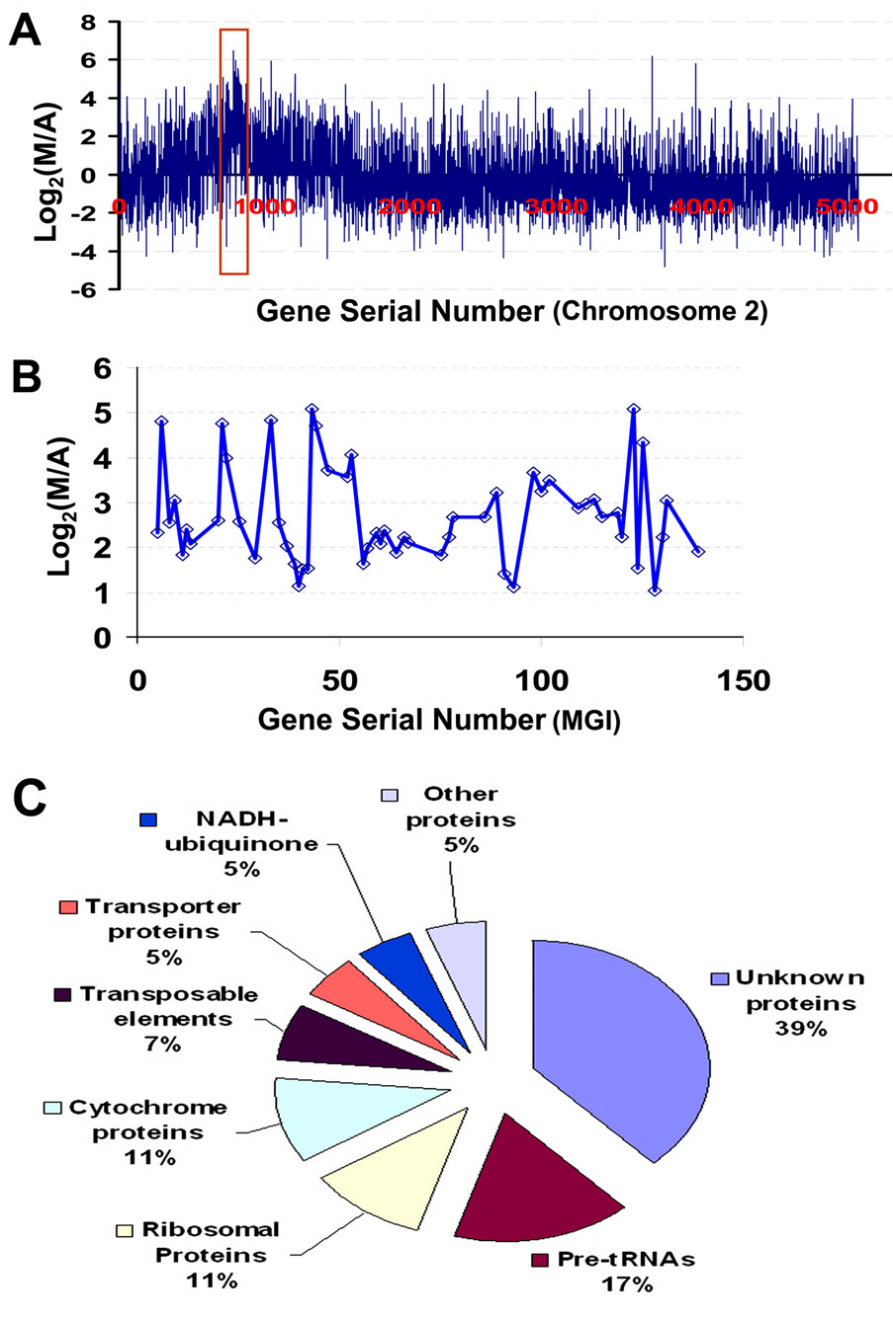
**Distribution of preferentially expressed MGI genes in meiocytes**. **A**. Distribution of genes with significantly differential expression on Chromosome II; **B**. A close up view, showing the distribution of differentially expressed genes on MGI; **C**. Classification of MGI genes that were preferentially expressed in meiocytes (also see the Additional file 4, Table S2). M = meiocyte, A = anther.

A large genomic block on chromosome II pericentromeric region was found to have genes expressed preferentially in meiocytes. The genomic block included a ~270 KB region that was reported to be of mitochondrial genome origin [59,60], which was believed to be approximately 620 KB with repeated mitochondrial genomic fragments and an unsequenced gap [61]. In our analysis, 152 genes were found in the region spanning from At2G07650 to At2G08986 (chromosome positions 3222935-3626460) (Figure 5). With the cutoff of one read per million reads in meiocytes, 100 genes on this block were preferentially or specifically expressed in meiocytes versus anthers. Among the 100 genes, 45 genes were considered to be only expressed in meiocytes with less than one read per million reads in anthers, and 55 genes were preferentially expressed in meiocytes, including 40 genes that were expressed at fourfold or greater in meiocytes than in anthers (Figure 5B and Additional file 4, Table S2).

### Transposable element genes expressed in meiocytes

**Figure 6:**
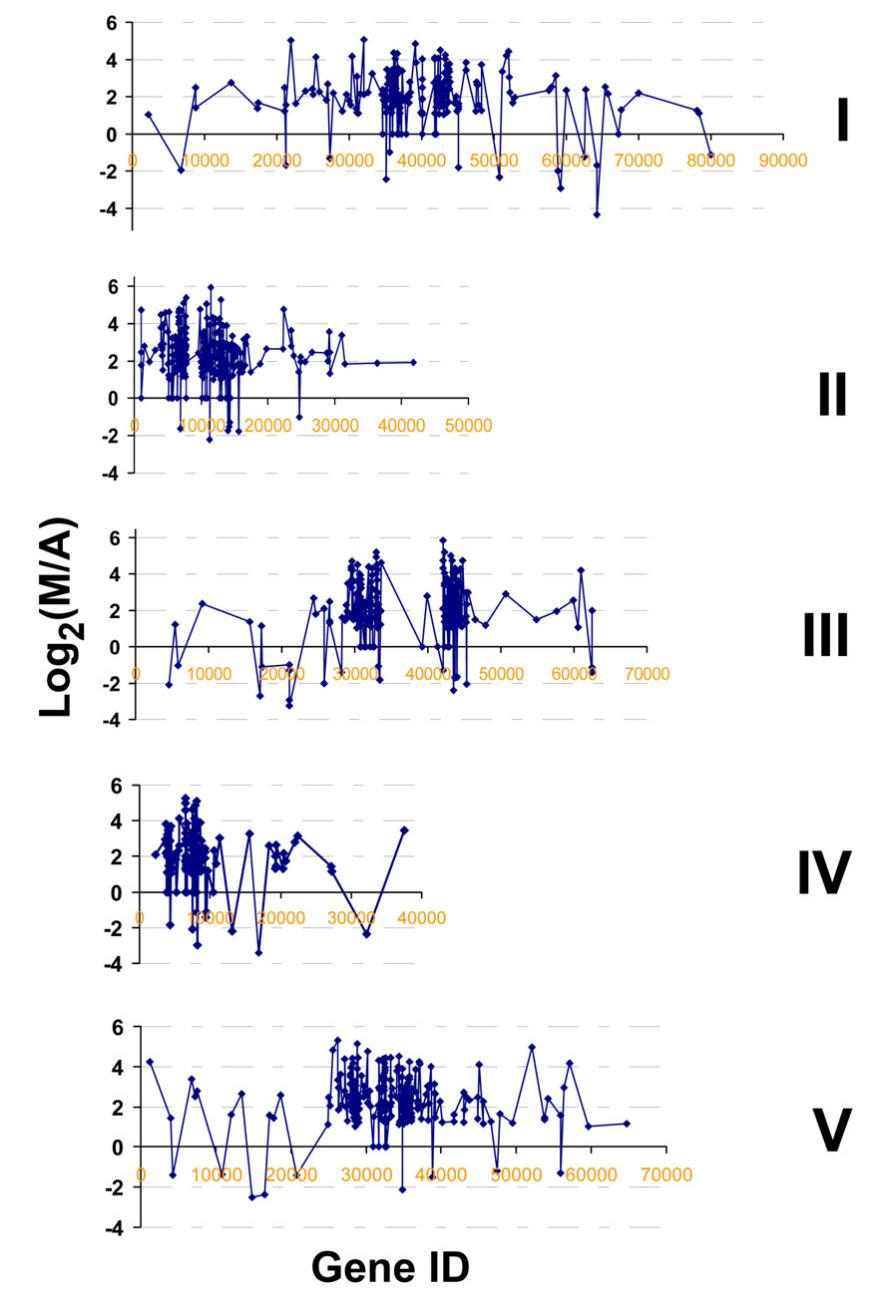
**Chromosomal distribution of differentially expressed TEs in meiocytes and anthers**. The preferentially and specifically expressed TEs in meiocytes are concentrated at pericentromeric regions of all chromosomes. M = meiocyte, A = anther. I, II, III, IV, and V refer to the chromosome numbers.

Transposable elements (TEs) are a ubiquitous feature of plant genomes. At a cutoff of one read per million reads, 1,271, 138, 379 TE genes were expressed in meiocytes, seedlings and anthers, respectively. In this study, a total of 1,117 TE genes demonstrated differential expression in meiocytes and anthers with at least a twofold difference (Additional file 5, Table S3). Among the 1,117 TE genes, 871 genes were only expressed in meiocytes with no reads or less than one read per million reads in anthers, such as At3G30846, At2G13110; and only 18 genes were only expressed in anthers. 228 genes were detected in both meiocytes and anthers with differential expression, including 165 genes that were preferentially expressed in meiocytes versus anthers, i. e. At2G07080, At5G34851, and 63 genes were preferentially expressed in anthers versus meiocytes, such as At1G64270, At4G16870 (Additional file 5, Table S3). Together, there were 1,036 TE genes up-regulated and 81 TE genes down-regulated in meiocytes versus in anthers. Since the TE genes are enriched in the pericentromeric regions, as shown in Figure 6, the distribution of differentially expressed TEs in meiocytes versus anthers is also seen at pericentromeric regions in all 5 chromosomes (Figure 6, Additional file 6, Figure S3 and Additional file 5, Table S3).

**Figure 7:**
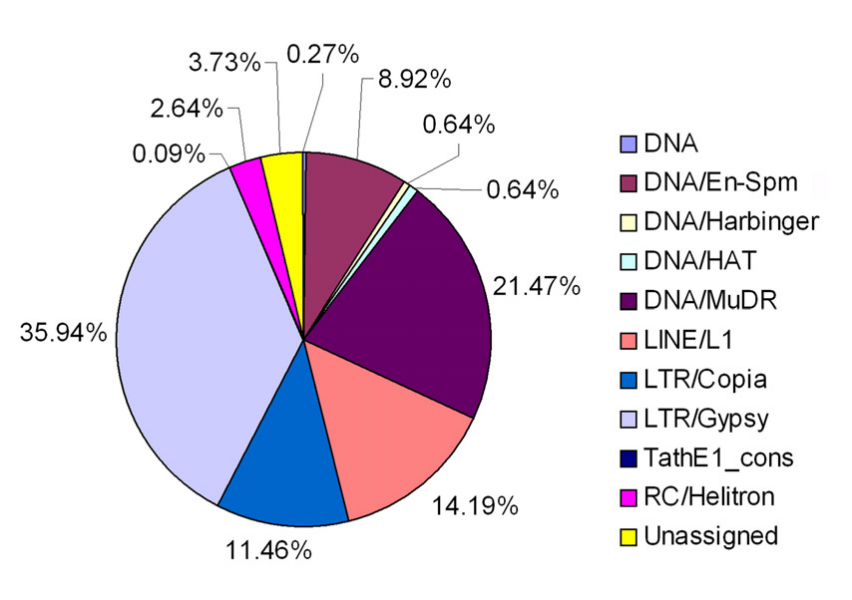
**Super-family distribution of differentially expressed TEs in meiocytes and anthers**.

GO analysis has demonstrated that meiocyte preferentially expressed TE genes belong to 10 TE super families with 3.73% of TEs that are unassigned (Figure 7, Additional file 5, Table S3). 35.94% of differentially expressed TEs detected in meiocytes are LTR/Gypsy super family transposons, and 21.47% of belong to the DNA/MuDR super family. Additional details are also presented (Figure 7, Additional file 5, Table S3). A tree-map of functional categories using Agrigo-Revigo toolkit is also provided (Additional file 7, Figure S4).

A comparative analysis of TE gene expression in meiocytes versus seedlings resulted in a total of 1,223 differentially expressed TE genes (Additional file 8, Table S4). Among the 1,223 TE genes, 1,148 genes were meiocyte-specific with no reads or less than one read per million reads in seedlings; and only 17 genes that were seedling-specific. 58 genes were detected in both meiocytes and seedlings with differential expression, including 27 genes that were preferentially expressed in meiocytes, and 31 genes were preferentially expressed in seedlings. Together, there were 1,165 TE genes up-regulated and 48 TE genes down-regulated in meiocytes versus in seedlings (Additional file 8, Table S4).

## Discussion

### Refining the criteria for meiotic gene identification by profiling studied genes that function in meiosis

68 genes in *Arabidopsis *have been identified and characterized with function in meiosis using forward and reverse genetic approaches (Additional file 1, Table S1). The 68 known *Arabidopsis *genes with functions in meiosis can be assigned to four functional categories: 1) genes which function in homologous chromosome pairing and recombination (e.g., *AtSPO11-1, AtDMC1, AtRAD51, AtRAD51c, AtXRCC3, AtMSH4, AtMER3/RCK*) [40,45,46,55]; 2) genes that encode structural proteins such as cohesin, histone, centromere proteins and proteins for synaptonemal complex assembly, e.g., *SYN1/DIF1, SMC1, ZYP1*, and *ASY1 *[62-66]; 3) genes which function in chromosome spindle organization and movement (e.g. *ATK1, AESP, ATK5/AtKin14B, AtPS1*) [58,67-70]; and 4) genes which encode regulatory proteins such as *MMD1/DUET, SDS, TAM*, and *ASK1 *[54,71-74]. By profiling the gene expression of all 68 genes in meiocytes and control tissues, meiosis-specific candidate genes can be efficiently identified by following the two criteria: 1) genes are expressed at twofold or greater in meiocytes versus anthers; and/or 2) genes are expressed at twofold or greater in both meiocytes and anthers versus seedlings with the exclusion of genes that are expressed at fourfold or greater in anthers versus meiocytes.

Three genes, *MND1*, *AtSRP2*, *AtSRP3*, were expressed at very low levels and only had 0.7 read per million reads in meiocytes, which suggests more sequence data or a lower cutoff point is needed in order to cover all important meiosis-specific genes for these datasets [33,35,36]. Only 4 genes (*AtSPO11-2*, *MS5*, *MND1 *and *AtSRP2*)[32,33,36,75] meet the first criterion with preferential expression in meiocytes, and 29 genes were expressed twofold or greater in both meiocytes and anthers comparing to seedlings, which include key meiotic recombination genes, such as *AtSPO11-1*, *AtDMC1*, *ASY1, AtMLH3, AtRAD51C*, *AtXRCC3*, *AtMSH4*, *AtMSH5*, *AtMER3/RCK*, *PTD*, *AtMUS81*, and *SDS *[49,76-78] (Additional file 1, Table S1). Genes that do not meet the two criteria are unlikely to be meiosis-specific; for example, *ATK5/KIN14B *may also have important roles in mitotic cell division [58,68]. *AtRAD51 *has a meiosis-specific function in *Arabidopsis*, but it is expressed in both meiocytes and non-meiotic somatic cells [41], which is consistent to the *RAD51 *gene expression in other organisms, such as mice B cells [79]. Most of the meiosis-specific genes, especially for those in the meiotic recombination pathways could be identified by comparative analysis of transcriptome profiles of meiocytes, anthers and seedlings [6-8,55] (Additional file 1, Table S1).

A visual representation of all statistically different genes from the ANOVA is presented as in the Ward Hierarchical Clustering, which suggests a number of genes that are uniquely expressed in the anther as well as the meiocyte that may be unique candidate genes for control of expression (Figure 3). As shown in Figure 4, with a cutoff point of 5 reads per million reads in at least one sample, more than 1,000 candidate meiosis-specific genes were identified through this approach with an additional 607 genes that are preferentially expressed in meiocytes (Figure 4).

The main purpose of profiling concentrated meiocytes is to eliminate genes that are expressed and function in anther wall development, which is critical, because those genes would be included in the candidate gene pools for entire anther development if transcriptomic analysis is performed using anther materials [5]. A criterion we suggest for meiotic gene identification is to exclude genes that are expressed at fourfold or greater in anthers versus meiocytes, although the gene expression level may be up-regulated in both meiocytes and anthers versus seedlings. For example, *DYT1 *was found in a gene pool by profiling anther transcriptome compared to other organs, and functions in regulating anther wall development [16,80]. Here we show that *DYT1 *was read at 7.8, 89.3, 0.0 reads per million reads in meiocytes, anthers, and seedlings, respectively. The differential expression indicates that *DYT1 *is specifically expressed in anthers/meiocytes with a significantly preferential expression in anthers, which is consistent to its function in anther wall development [80]. Another example is the *ATA1 *gene that was reported to be highly expressed in tapetum [81], and the RNA-Seq results read at 33.4, 564.6, 0.0 reads per million reads in meiocytes, anthers and seedlings, respectively. Both *DYT1 *and *ATA1 *were preferentially expressed in anthers versus meiocytes, which implies the feasibility of excluding the anther wall genes by comparative analysis of transcriptome profiling of meiocytes, anthers and seedlings. As indicated in criterion 2 for meiosis-specific gene identification, genes that are expressed at fourfold or greater in anthers versus meiosis should be considered as non-meiosis-specific candidate genes, or candidate genes for anther wall development.

Although this study has not included a parallel transcriptome study of microspore/gametophyte, the life after meiosis, a comparison of meiocytes (transition from diploid sporophyte to haploid gametophyte) should advance our understanding of the molecular connections between the two key processes of reproduction development, as well as promoting the means of identification of meiosis-specific genes. Previously, pollen transcriptome profiling using microarrays have found 7,235 genes expressed in *Arabidopsis *Landsberg *erecta *with 387 pollen-specific [14] and 6,587 expressed in *Arabidopsis *Col-0 ecotype[13]. Since the meiocytes we collected included tetrads, there is likely to be a significant overlap between meiocytes and microspores. A further deep transcriptome sequencing using staged meiosis-I meiocytes is currently being performed.

### Genes in a mitochondria genomic insertion on chromosome II were preferentially expressed in meiocytes

Previously, genome sequence analyses of chromosome II revealed a 270 kb chromosome region located on the short arm adjacent to the centromere and was annotated as a putative mitochondrial genome insertion (MGI)[59]. This annotation was adopted by TAIR [http://www.Arabidopsis.org] and Salk T-DNA Express: the *Arabidopsis *Gene Mapping Tool (http://signal.salk.edu/cgi-bin/tdnaexpress). Fiber FISH analysis uncovered the structure of the MGI in Col-0 ecotype as an approximately 620 kb mitochondrial genomic insertion with several duplicated segments and events [61]. Thirty-two orphan RNAs were found on this region [82]. Since this region is in the genetic centromere region and thought to be of mitochondrial origin, the function and transcript profile of this genomic block remains unknown. Our data showed that all 55 genes detected in both meiocytes and anthers were preferentially expressed in meiocytes, in which 40 genes were expressed at fourfold or greater in meiocytes versus anthers. (Figure 5B). 39% of the 55 genes encode unknown proteins, 17% for pre-tRNAs, others are ribosomal proteins (11%), cytochrome proteins (11%), transposable elements (7%), transporter proteins (5%) and NADH-ubiquinone proteins (5%), which suggest this group of genes may function in meiosis (Figure 5C). A preliminary investigation on the T-DNA insertion lines targeted on MGI genes confirmed that some mutants on the MGI block have meiotic phenotypes (Chen, et al., unpublished results), which further suggest the functions of MGI in meiosis in the Col-0 ecotype of *Arabidopsis*.

### Transposable element genes in meiosis

While transposable elements (TEs) make up to 14% of *Arabidopsis *genome [83], the majority of TEs were silenced during plant development since there were a lack of mRNAs, but higher levels of small RNAs were detected [84]. TEs' activities were usually limited in just one or a few of developmental stages, in which TEs were expressed [84]. In comparing the transcriptomes of anther and meiocytes, we observed a large set of TEs that were expressed preferentially or specifically in meiocytes versus anthers. At a cutoff point of one read per million reads, a total of 1,271 TE genes were expressed in meiocytes, which is about 32.5% of 3,907 TE genes reported or annotated in *Arabidopsis *[84] [http://www.arabidopsis.org]. Relatively smaller numbers of TE genes were expressed in controls: 379 and 138 in anthers and seedlings, respectively. With 1,036 TE genes up-regulated and 81 TE genes down-regulated in meiocytes compared to anthers, TE genes may play unique roles in meiosis. In addition, 1,165 TE genes were up-regulated and 48 TE genes down-regulated in meiocytes as compared to seedlings, which are consistent with the comparison between meiocytes and anthers, and demonstrated more significant deviation between meiocytes and seedlings (Additional file 5, Table S3, and Additional file 8, Table S4).

The abundant TE expression in meiocytes suggests substantial activities of TEs in meiosis. It is believed that TEs affect recombination in all meiotic eukaryotes [85]. Recent studies on postmeiotic gametophyte development have found that TE genes are unexpectedly reactivated and transpose only in the vegetative nucleus, but not in the sperm cells of pollen [86], which suggest that small interfering RNAs (siRNAs) from TEs activated in the vegetative nucleus can target silencing those in gametes [86]. In addition, small RNA pathways have been found to be present and functional in the angiosperm male gametophytes [87]. During female gamete formation, AGO9 was found to preferentially interact with siRNAs derived from TEs and the activity of AGO9 is required to silence TEs in female gametes and their accessory cells [88]. The AGO9 gene (At5G21150) was preferentially expressed in anthers versus meiocytes (M/A = 78.24/172.37) in this study, which is consistent with the discovery of a postmeiotic function. In contrast, the two AGO genes, At1G31290 (AGO3) and At5G21030 (AGO8) were preferentially or specifically expressed in meiocytes (At1G31290: M/A = 11.54/3.30; At5G21030: M/A = 3.03/0.71), which suggest that molecules regulating gene silencing and DNA modification in meiosis differ from those of postmeiotic gametophyte development, both in the male and the female. In the postmeiotic gametes, the gene expression map has demonstrated the similarities between plants and animals [89], which may also be true in meiosis. To date, it still remains largely unknown how TEs function in meiosis. It is possible that a large number of TEs are activated in meiosis and then silenced after meiosis through siRNA machinery and/or modification of heterochromatin.

## Conclusion

A high-throughput approach to identify meiosis-specific genes by comparative profiles of meiocyte, anther and seedling transcriptomes using RNA-Seq, bioinformatic and statistical analysis pipelines was established. Two criteria for meiosis-specific gene identification were defined. Using this method, thousands of genes that are preferentially expressed in anthers would be excluded from a meiosis-specific candidate gene pool; a MGI block was found to be specifically expressed during meiosis; and 1,036 transposable element genes were also preferentially expressed in meiocytes with potential functions in meiosis. These findings provide a framework for future functional analysis of genes in meiosis and advance our understanding of meiotic genes, gene expression and regulation.

## Methods

### Plant and growth conditions

The Col-0 ecotype of wild-type *Arabidopsis *used in this study was grown in plant growth chambers at 22°C/20°C (day/night), in 65% humidity with a photoperiod of 16/8 (day/night).

### Microscopy

Inflorescences and young flower buds were dissected using an Olympus SZ40 stereo microscope (Olympus Co., Tokyo, Japan). Inflorescence and anther photos were taken using a SPOT digital camera (Diagnostic instruments, Inc., Sterling Heights, MI, USA). Meiocytes were collected using a modified inverted microscope (see below). For photographs, one hour collections were briefly centrifuged at 1,000 g for 30 second and resuspended in 5 μl 0.1% Toluidine Blue O in PBS buffer (pH = 7.0). Anther thin sections were prepared as previously described [90].

### Male meiocyte collection, and anther and seedling preparation

A manual *Arabidopsis *meiocyte collection method, the *Capillary Collection of Meiocytes *(CCM) was designed for efficient meiocyte sampling. Briefly, anthers undergoing meiosis were collected (Figure 1A) then squashed using a sharp clean forceps to release meiocytes. Capillary glass pipettes were used to collect meiocytes under an inverted microscope (Figure 1C). In this study, meiocytes cover all stages of meiosis, from leptotene to tetrad, were collected, although the CCM allows collecting of staged meiotic cells, especially meiosis I-meiocytes. Meiocytes were transferred to an Eppendorf tube containing 500 μl of RNA*later *(Ambion #7020), and were kept at 4°C for up to one month for multiple collections to achieve the desired cell number. Before extracting RNA, meiocytes were centrifuged at 10,000 g for 5 minutes. The cell pellets were either frozen at -80°C or used to extract RNA directly. One week (~25 hours) of collection resulted in approximately 28,000 cells with a purity of 98% meiocytes, which were used to extract total RNA for building the sequencing library.

For controls, 600 of stage 5-7 anthers were directly dissected and collected from stage 9 flower buds and stored in RNA*later *for up to 4 weeks before RNA extraction. For collecting seedling samples, the *Arabidopsis *Col-0 seeds were sowed in soil mix directly and transferred to a growth chamber for 2 weeks. 10 seedlings were then carefully harvested and soil removed using running water. Whole seedlings including shoots, leaves and roots were then put on paper towel for a few seconds to remove extra water and stored in RNA*later*.

### RNA extraction and measurement

Total RNA from meiocytes, anthers and seedlings were extracted using the Ambion RNAqueous^®^-Micro kit (Ambion, #AM1931) according to the manufacturer's instructions. The total RNA yields were measured using Invitrogen^® ^Qubit™ fluorometer and Agilent Bioanalyzer 2100 microfluidics (Agilent, Santa Clara, CA). 8.04 μg of total RNA resulted from the 28,000 meiocyte collection. 5.15 μg and 4.80 μg of total RNA were extracted from anther and seedling controls, respectively.

### Sequencing library preparation

Poly-A containing RNA was isolated from total RNA using oligo-dT_25 _magnetic beads (Dynabeads; Invitrogen, Carlsbad, CA). After chemically reversing the Dynabead binding, the RNA was denatured and used as template for random-primed cDNA synthesis. The resulting cDNA was end-repaired by incubation in the presence of T4 DNA polymerase, Klenow polymerase and dNTPs. The polished fragments were phosphorylated by T4 polynucleotide kinase, followed by the addition of a single "A" base to the 3'-end of the blunt-ended phosphorylated DNA fragments. The fragments were then ligated to an Illumina adapter oligo which has an overhanging 3'-T. Ligation products were size-selected by electrophoresis for 1-2 hours at 80-110 V in low T_m _agarose containing ethidium bromide with size markers. The gel was visualized with a brief UV exposure, and the desired size range (300-500 bp) was excised. Purified DNA libraries were amplified by PCR for 15 cycles. Libraries were qualitatively and quantitatively assessed by Nanodrop ND-1000 (Thermo Scientific, Waltham, MA) UV/Vis spectroscopy and DNA BioAnalyzer 2100 microfluidics (Agilent, Santa Clara, CA).

### Transcriptome sequencing

Two picomoles per channel of the size-selected meiocyte cDNA library was loaded onto an Illumina single-end flow cell using the Illumina Cluster Station (Illumina, Inc., San Diego, CA). Anther and seedling libraries were sequenced as parallel controls. 36 bp reads were collected on an Illumina Genome Analyzer using sequencing-by-synthesis technology [91,92]. Image data acquired from the sequencing run was base-called and quality analyzed with the Illumina Genome Analysis Pipeline software package. Technical replicates were performed on different days and instruments. Approximately 21, 17 and 13 million reads were collected from meiocyte, anther and seedling libraries, respectively, with an average Illumina quality score of 31.

### Data analysis and de novo assembly of transcriptome

Reads were aligned to the TAIR Release 9 of the *Arabidopsis *genome and its associated annotations and gene calls using GSNAP [93], the follow-on program to GMAP [94]. This program aligns short read data to the reference genomes and transcriptomes, with accommodation for sequencing errors, indels and alternative splicing. The alignment was managed through a pipeline associated with the Alpheus data management software [95], and loaded into the Alpheus-associated database for further analysis. The gene expression functionality in Alpheus was used to provide basic normalization and to return genomically-aligned reads per million per library. Candidate differential gene expression was developed by querying the database for read counts which were different in bi-directional comparisons between each of the tissue pairs.

In addition to alignments to the genome, reads were assembled *de novo *using a hybrid assembly technique. Reads were preliminarily assembled using ABySS-P [96,97] and SSAKE [98], and the results of these assemblies merged using PCAP [99] to create the final assembly. ABySS-P uses a parallel implementation of a de Bruijn graph for assembly; SSAKE uses a stringent prefix tree. Both robustly handle large numbers of short reads to give an initial contig assembly. PCAP uses a more classical overlap-layout-consensus approach to combine and extend contigs. GO analysis was performed using TAIR GO tools [http://www.Arabidopsis.org]. The Agrigo-Revigo toolkit was applied for the classification and annotation of TEs [100].

### Visualization of genomic alignments

In addition to the individual read visualization in the Alpheus software [95], data was extracted from the database and loaded into a modified version of the Comparative Map and Trait Viewer [101]. This visualization software allows the simultaneous viewing of the genomic tract, a representation of the TAIR annotations and reads from individual libraries and technical replicates.

### Statistical analysis of sequence-based differential expression

Illumina GA reads that aligned to the genomic *Arabidopsis *TAIR9 database were normalized by total reads per million, and analysis was limited to one or more total reads per million in at least one of the five samples. Technical replicates were performed on anther and meiocytes. To provide a technical replicate for the seedling controls, the seedling reads were split into two unique columns based from the position of the polony (each lane on the flow cell contains two columns that are imaged - the columns were split into two separate columns and were individually normalized by total reads per million). All six samples were then log2+1 transformed. Statistical analysis was performed as previously described [102]. Briefly, normal distribution was determined by overlaid kernel density estimates, univariate distribution results, Mahalanobis distances, correlation coefficients of pairwise sample comparisons, unsupervised principal component analysis (by Pearson product-moment correlation) and Ward hierarchical clustering of Pearson product-moment correlations of read frequencies were performed with JMP Genomics, Version 4.0 (SAS Institute, Cary, NC). Analysis of variance against all pairwise sample comparisons was performed with a 5% false discovery rate (FDR).

### Meiotic gene identification and functional analysis

By comparing the sequencing datasets from three tissues, genes which were differentially expressed in the different organs suggest a potentially tissue-specific function. Genes that were expressed only in meiocytes or at twofold or greater in meiocytes versus anthers were considered as candidates for meiosis-specific genes. In addition, we identified novel candidate genes or exons by comparing the alignment of the *Arabidopsis *transcriptome and gene predictions to those reads which aligned to unannotated regions of the genome.

## List of abbreviations

CCM: capillary collection of meiocyte; MGI: mitochondrial genomic insertion; GO: gene ontology; TE: transposable element.

## Competing interests

The authors declare that they have no competing interests.

## Authors' contributions

CC, GDM, AGS, EFR designed the study and contributed to write the manuscript. CC collected male meiocytes and prepared total RNA samples for RNA-Seq, GDM and JH performed RNA-Seq and deposited datasets in Alpheus, ADF, RJL, JM, JC, CC and EFR processed and analyzed the datasets. All authors contributed to the manuscript preparation, and read and approved the final manuscript.

## Supplementary Material

Additional file 1 **Table S1. Transcript profiling of genes that function in meiosis**. Transcript profiling of 68 previously reported genes that function in meiosis. Showing the signal intensity by reads per million reads. M = meiocyte, A = anther, S = seedling. Click here for file

Additional file 2 **Figure S1. Parallel plot to demonstrate the similarity of technical replicates**. Showing high similarity of technical replicates. Red--anther; green--meiocyte; blue--seedling. In this figure, no technical replicates for seedlings were presented. Click here for file

Additional file 3 **Supplementary Figure S2. Figure S2. Scatterplot matrix to demonstrate the correlations among all samples**. The pairs plots show the correlations among all samples. Anther_control = anthers; Anther_meiosis = meiocytes. Click here for file

Additional file 4 **Table S2. A list of MGI genes that are preferentially expressed in meiocytes**. SN = serial number, MGI = mitochondrial genomic insertion, M = meiocyte, A = anther. Click here for file

Additional file 5 **Supplementary Table S3. Table S3. A list of differentially expressed TEs in meiocytes and anthers**. The list of differentially expressed TEs in meiocytes and anthers, the label of "--" refers to zero (0) reads from anther. In addition to the mRNA signal intensity of read counts (normalized as reads per million reads), this table also provides gene ID, transposon ID, transposon family and super family. The shaded rows are genes that down-regulated in meiocytes and preferentially expressed in anthers. M = meiocyte, A = anther. Click here for file

Additional file 6 **Figure S3. Distribution of expressed mRNAs in meiocytes among gene function categories**. Percentage of gene distribution and raw data are presented next to each category. Click here for file

Additional file 7 **Figure S4. Distribution of expressed TEs in meiocytes among gene function categories**. Treemaps of expressed TEs in meiocytes generated by REVIGO. In each category, the size of the rectangle is proportional to the population of functional groups. **A**. Biological process. **B**. Cellular component. **C**. Molecular function. Click here for file

Additional file 8 **Table S4. A list of differentially expressed TEs in meiocytes and seedlings**. The list of differentially expressed TEs in meiocytes and seedlings, the label of "--" refers to zero (0) reads from seedling. The shaded rows are genes that down-regulated in meiocytes and preferentially expressed in seedlings. M = meiocyte, S = seedling. Click here for file
