# Supplementary material for: Meiosis-specific gene discovery in plants: RNA-Seq applied to isolated Arabidopsis male meiocytes
Source: BMC Plant Biol. 2010 Dec 17;10:280. doi: 10.1186/1471-2229-10-280 (PMC3018465; doi:10.1186/1471-2229-10-280)
Supplement: Additional file 1 — Table S1. Transcript profiling of genes that function in meiosis. Transcript profiling of 68 previously reported genes that function in meiosis. Showing the signal intensity by reads per million reads. M = meiocyte, A = anther, S = seedling. [file 1471-2229-10-280-S1.PDF]

Supplemental Table S1. Transcript profiling of genes that have functions in meiosis

Gene ID	Name/Description	Anther	Meiocyte	Seedling	M/A	M/S	A/S
AT4G22970	*AESP*	14.0	3.7	6.7	0.3	0.5	2.1
AT1G13330	*AHP2*	9.7	6.2	4.8	0.6	1.3	2.0
AT5G61960	*AML1*	144.5	106.5	66.1	0.7	1.6	2.2
AT2G42890	*AML2*	10.7	9.7	24.2	0.9	0.4	0.4
AT4G18120	*AML3*	45.8	17.9	32.1	0.4	0.6	1.4
AT5G07290	*AML4*	67.2	18.1	33.0	0.3	0.5	2.0
AT1G29400	*AML5*	43.2	28.5	90.4	0.7	0.3	0.5
AT1G75950	*ASK1*	289.6	96.1	94.3	0.3	1.0	3.1
AT5G42190	*ASK2*	70.6	58.1	82.8	0.8	0.7	0.9
AT1G67370	*ASY1*	34.1	10.4	0.8	0.3	12.7	41.4
AT5G40820	*AtATR*	21.1	11.5	8.2	0.5	1.4	2.6
AT3G52115	*AtGR1=COM1*	6.9	4.0	2.8	0.6	1.4	2.5
AT4G21270	*ATK1=KIN14A*	19.0	6.3	10.2	0.3	0.6	1.9
AT4G05190	*ATK5=KIN14B*	20.2	13.7	15.3	0.7	0.9	1.3
AT3G48190	*ATM*	86.0	37.1	38.3	0.4	1.0	2.2
AT4G35520	*AtMLH3*	7.9	7.6	0.8	1.0	9.2	9.6
AT5G54260	*AtMRE11*	15.0	9.8	7.0	0.6	1.4	2.1
AT3G18520	*AtMSH2*	70.1	29.3	39.7	0.4	0.7	1.8
AT4G17380	*AtMSH4*	11.3	6.0	0.2	0.5	36.5	68.7
AT3G20475	*AtMSH5*	27.0	12.9	0.8	0.5	15.7	32.8
AT4G30870	*AtMUS81*	18.7	17.4	4.4	0.9	4.0	4.3
AT4G14180	*AtPRD1*	5.9	4.7	1.1	0.8	4.4	5.5
AT5G57880	*AtPRD2=MPS1*	4.7	2.7	2.7	0.6	1.0	1.7
At1G01690	*AtPRD3*	14.7	3.3	5.0	0.2	0.7	2.9
AT1G34355	*AtPS1*	31.9	18.3	6.9	0.6	2.6	4.6
AT5G20850	*AtRAD51*	13.3	6.3	3.9	0.5	1.6	3.4
AT2G45280	*AtRAD51C*	14.6	12.5	4.4	0.9	2.9	3.3
AT5G63540	*AtRMI1*	3.8	2.1	0.7	0.6	2.9	5.2
AT2G06510	*AtRPA1A*	39.4	11.1	10.6	0.3	1.0	3.7
AT3G54670	*AtSMC1=TTN8*	96.0	83.0	44.9	0.9	1.8	2.1
AT3G47460	*AtSMC2=CAPE2*	23.6	9.4	21.2	0.4	0.4	1.1
AT5G19400	*AtSMG7*	76.4	40.2	64.6	0.5	0.6	1.2
AT1G28260	*AtSMG7L*	16.6	6.6	14.2	0.4	0.5	1.2
AT3G13170	*AtSPO11-1*	2.8	1.0	0.3	0.3	2.9	8.5
AT2G14540	*AtSRP2*	0.2	0.7	0.0	3.1	-	-
AT1G64030	*AtSRP3*	3.6	0.7	0.0	0.2	-	-
AT5G63920	*AtTOP3A*	27.5	6.3	10.8	0.2	0.6	2.6
AT5G57450	*AtXRCC3*	2.3	2.6	0.7	1.1	3.5	3.1
AT5G48390	*AtZIP4*	15.1	5.2	0.6	0.3	9.0	26.3
AT5G01630	*BRACA2B*	27.8	6.4	10.8	0.2	0.6	2.6
AT4G00020	*BRCA2A*	26.7	10.3	15.2	0.4	0.7	1.8
AT3G25100	*CDC45*	6.8	1.8	4.3	0.3	0.4	1.6
AT1G77390	*CYCA1,2=TAM*	12.8	5.8	1.0	0.4	5.8	13.0
AT5G05490	*DIF1=SYN1*	14.7	6.7	0.4	0.5	16.3	35.7
AT3G22880	*DMC1*	47.0	25.2	4.2	0.5	6.0	11.2
AT1G66170	*DUET=MMD1*	30.4	9.0	0.3	0.3	27.4	92.3
AT1G77320	*MEI1*	13.3	7.6	4.8	0.6	1.6	2.8

AT4G27170	*MND1*	0.1	0.7	0.1	11.5	8.1	0.7
AT1G63770	*MPA1*	131.4	29.1	123.7	0.2	0.2	1.1
AT4G20900	*MS5=TDM*	35.5	69.3	0.0	2.0	-	-
AT3G02680	*NBS1*	11.1	7.1	4.4	0.6	1.6	2.5
AT3G57860	*OSD1*	39.9	14.8	7.9	0.4	1.9	5.0
AT1G12790	*PTD*	12.9	10.1	2.0	0.8	5.1	6.5
AT2G31970	*RAD50*	40.4	19.1	25.1	0.5	0.8	1.6
AT3G27730	*RCK=AtMER3*	12.4	5.4	0.6	0.4	9.4	21.5
AT2G47980	*SCC3*	128.5	32.1	59.9	0.2	0.5	2.1
AT1G14750	*SDS*	9.5	5.0	1.5	0.5	3.4	6.4
AT5G52290	*SHOC1*	6.3	1.1	0.3	0.2	3.4	19.0
AT5G62410	*SMC2=TTN3=AtCAPE1*	38.1	13.9	21.0	0.4	0.7	1.8
AT2G27170	*SMC3=TTN7*	61.3	55.7	39.1	0.9	1.4	1.6
AT1G63990	*SPO11-2*	7.7	17.5	8.8	2.3	2.0	0.9
AT3G43210	*STD=TES=NACK2*	44.0	30.1	10.5	0.7	2.9	4.2
AT5G51330	*SWI1=DYAD*	9.4	3.2	0.1	0.3	39.2	113.9
AT5G40840	*SYN2=AtRAD21.1*	12.0	5.6	2.6	0.5	2.1	4.6
AT3G59550	*SYN3*	13.8	9.9	3.1	0.7	3.2	4.4
AT5G48720	*XRI1*	18.6	10.1	5.9	0.5	1.7	3.1
AT1G22260	*ZYP1A*	31.0	16.8	1.0	0.5	17.0	31.4
AT1G22275	*ZYP1B*	25.7	10.1	1.5	0.4	6.8	17.3
