# Supplementary figures and images for: Meiosis-specific gene discovery in plants: RNA-Seq applied to isolated Arabidopsis male meiocytes
Source: BMC Plant Biol. 2010 Dec 17;10:280. doi: 10.1186/1471-2229-10-280 (PMC3018465; doi:10.1186/1471-2229-10-280)

Parallel Plot

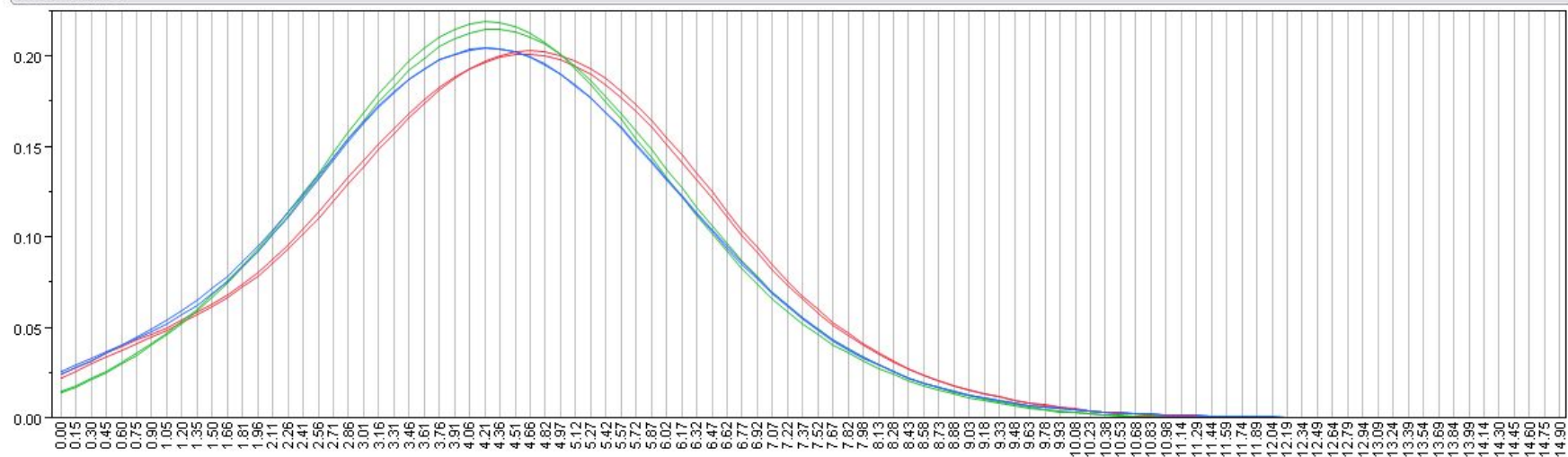

Supplement: Additional file 2 — Figure S1. Parallel plot to demonstrate the similarity of technical replicates. Showing high similarity of technical replicates. Red--anther; green--meiocyte; blue--seedling. In this figure, no technical replicates for seedlings were presented. [file 1471-2229-10-280-S2.PDF]

Scatterplot Matrix

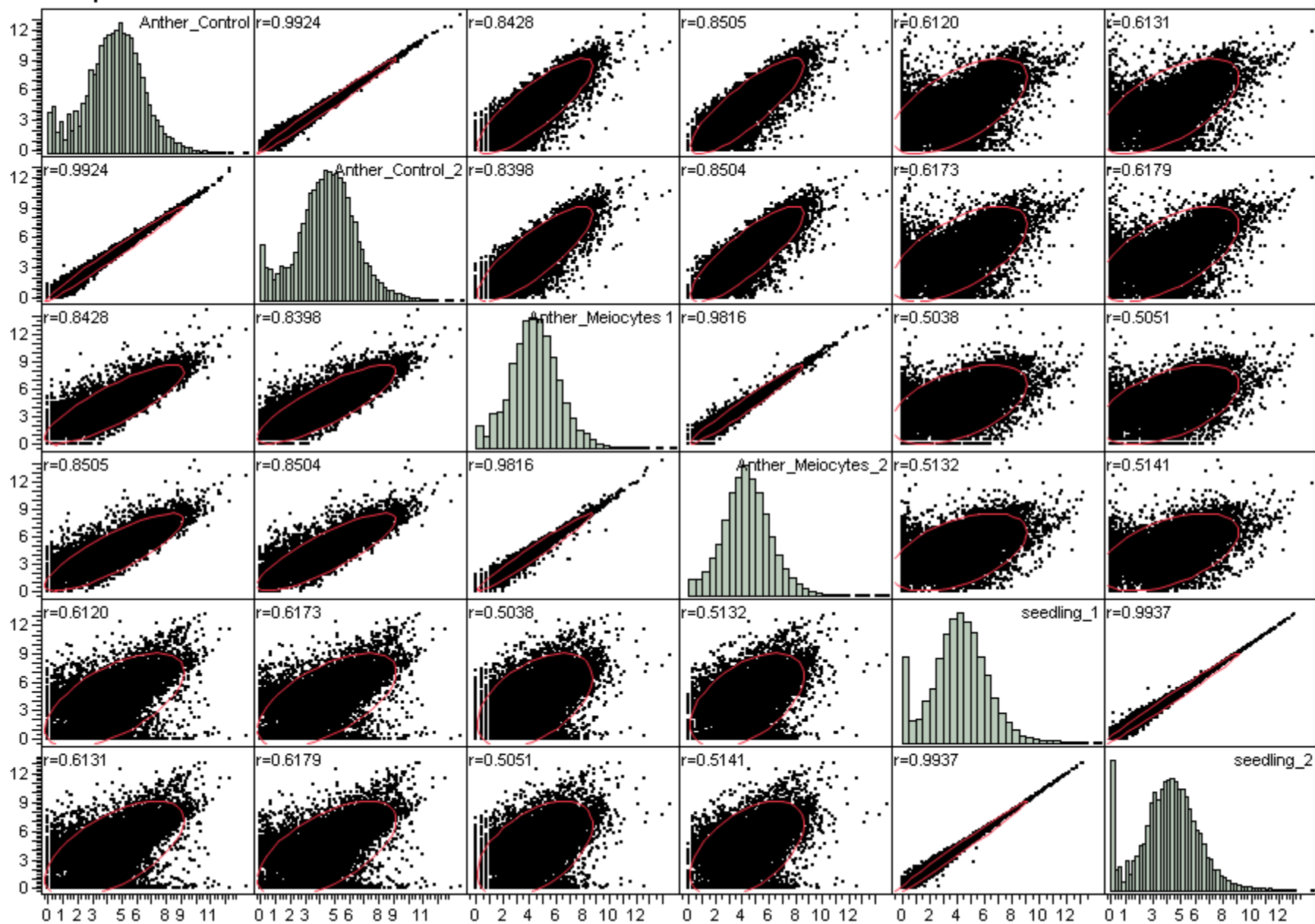

Supplement: Additional file 3 — Supplementary Figure S2. Figure S2. Scatterplot matrix to demonstrate the correlations among all samples. The pairs plots show the correlations among all samples. Anther_control = anthers; Anther_meiosis = meiocytes. [file 1471-2229-10-280-S3.PDF]

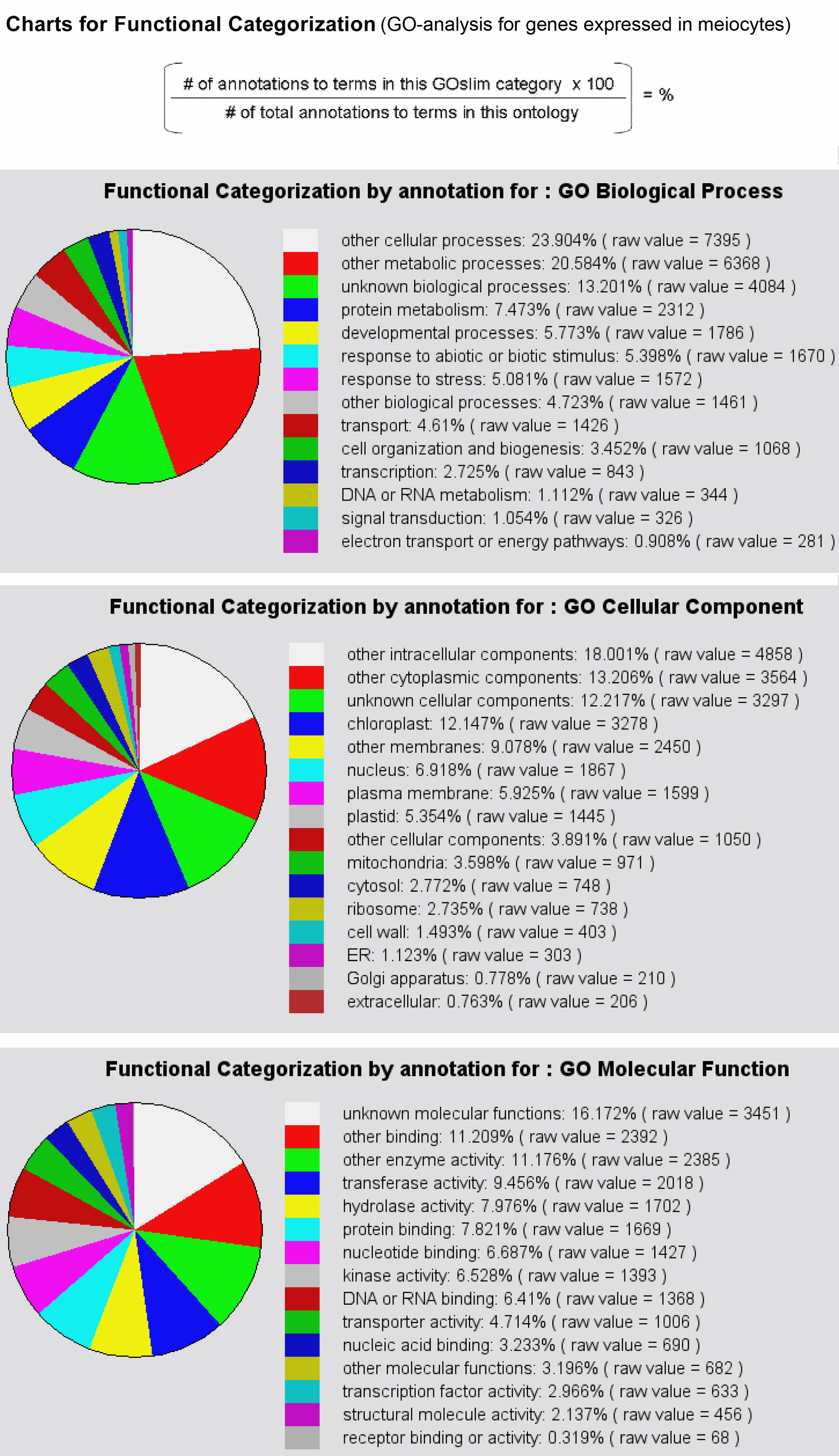

Supplement: Additional file 6 — Figure S3. Distribution of expressed mRNAs in meiocytes among gene function categories. Percentage of gene distribution and raw data are presented next to each category. [file 1471-2229-10-280-S6.JPEG]

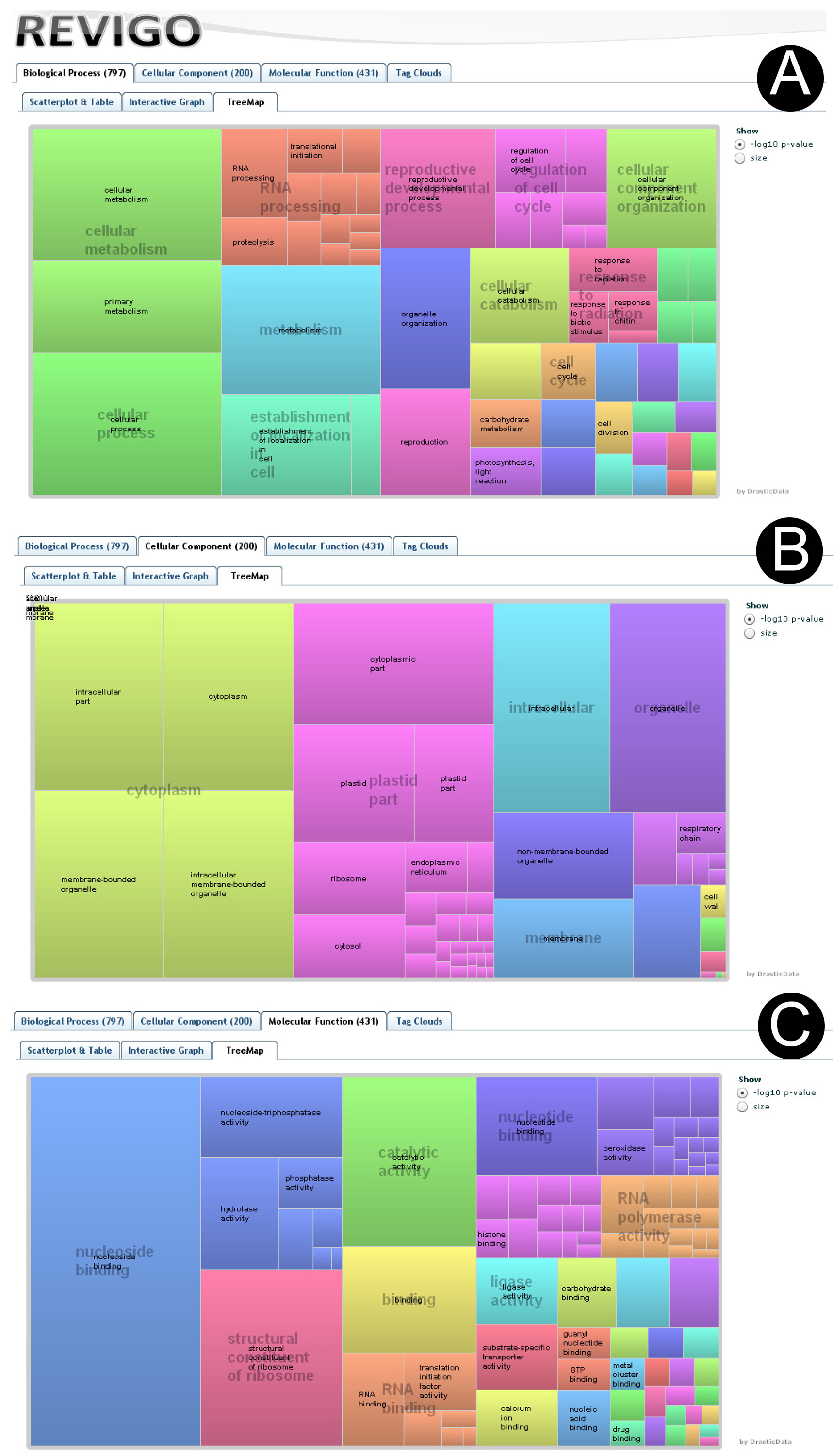

Supplement: Additional file 7 — Figure S4. Distribution of expressed TEs in meiocytes among gene function categories. Treemaps of expressed TEs in meiocytes generated by REVIGO. In each category, the size of the rectangle is proportional to the population of functional groups. A. Biological process. B. Cellular component. C. Molecular function. [file 1471-2229-10-280-S7.JPEG]
