# Supplementary material for: Meiosis-specific gene discovery in plants: RNA-Seq applied to isolated Arabidopsis male meiocytes
Source: BMC Plant Biol. 2010 Dec 17;10:280. doi: 10.1186/1471-2229-10-280 (PMC3018465; doi:10.1186/1471-2229-10-280)
Supplement: Additional file 4 — Table S2. A list of MGI genes that are preferentially expressed in meiocytes. SN = serial number, MGI = mitochondrial genomic insertion, M = meiocyte, A = anther. [file 1471-2229-10-280-S4.PDF]

Supplemental Table S2. Transcripts that are preferentially expressed in meiocytes versus anthers on MGI region

Gene ID	Description	SN of MGI	M	A	M/A	Log2
AT2G07771	cytochrome c biogenesis protein-related	5	7.8	1.5	5.0	2.3
AT2G07773	unknown protein	6	358.6	12.6	28.2	4.8
AT2G07776	unknown protein	8	6.7	1.1	5.8	2.5
AT2G07749	unknown protein	9	11.3	1.3	8.3	3.0
AT2G07777	unknown protein	11	19.1	5.3	3.5	1.8
AT2G07671	H+-transporting two-sector ATPase, C subunit family protein	12	156.5	29.3	5.3	2.4
AT2G07779	unknown protein	13	5.8	1.3	4.2	2.0
AT2G07674	unknown protein	20	15.4	2.5	6.0	2.6
AT2G07751	NADH-ubiquinone oxidoreductase chain 3, putative	21	293.9	10.8	27.1	4.7
AT2G07675	ribosomal protein S12 mitochondrial family protein	22	404.1	25.5	15.8	3.9
AT2G07768	heme transporter	25	7.0	1.1	5.9	2.5
AT2G07748	pre-tRNA	29	8.2	2.4	3.3	1.7
AT2G07679	ribosomal protein, putative	33	357.4	12.5	28.5	4.8
AT2G07681	cytochrome c biogenesis protein, putative	35	7.4	1.2	5.8	2.5
AT2G07682	transposable element gene	37	26.2	6.4	4.0	2.0
AT2G07772	unknown protein	39	9.2	2.9	3.0	1.6
AT2G07683	transposable element gene	40	13.3	6.0	2.2	1.1
AT2G07752	pre-tRNA	41	3.6	1.2	2.8	1.5
AT2G07753	pre-tRNA	42	4.0	1.4	2.8	1.5
AT2G07754	pre-tRNA	43	64.6	1.8	34.1	5.0
AT2G07774	unknown protein	44	135.6	5.1	26.3	4.7
AT2G07687	cytochrome c oxidase subunit 3	47	39.6	3.0	13.0	3.7
AT2G07792	pre-tRNA	52	17.1	1.4	11.8	3.5
AT2G07689	NADH-ubiquinone oxidoreductase, putative	53	19.2	1.1	16.7	4.0
AT2G07692	unknown protein	56	3.5	1.1	3.1	1.6
AT2G07693	transposable element gene	57	5.4	1.3	3.9	1.9
AT2G07695	cytochrome c oxidase subunit II, putative	59	19.0	3.8	5.0	2.3
AT2G07785	NADH-ubiquinone oxidoreductase, putative	60	21.8	5.1	4.2	2.0
AT2G07696	ribosomal protein S7 family protein	61	11.0	2.1	5.1	2.3
AT2G07798	unknown protein	64	9.3	2.5	3.7	1.8

AT2G07698	ATP synthase alpha chain, mitochondrial, putative	66	174.9	36.9	4.7	2.2
AT2G07667	unknown protein	67	5.5	1.2	4.3	2.1
AT2G07706	unknown protein	75	18.0	5.0	3.5	1.8
AT2G07707	hydrogen ion transmembrane transporter	77	27.5	5.9	4.6	2.2
AT2G07708	unknown protein	78	36.2	5.6	6.4	2.6
AT2G07715	ribosomal protein L2, putative	86	18.7	2.9	6.4	2.6
AT2G07718	cytochrome b, putative	89	14.9	1.6	9.3	3.2
AT2G07719	unknown protein	91	17.4	6.5	2.6	1.4
AT2G07722	unknown protein	93	17.9	8.3	2.1	1.1
AT2G07725	ribosomal protein L5 (RPL5)	98	32.1	2.5	12.6	3.6
AT2G07727	cytochrome b (MTCYB) (COB) (CYTB)	100	75.8	8.0	9.4	3.2
AT2G07728	unknown protein	102	22.5	1.9	11.3	3.5
AT2G07732	ribulose-bisphosphate carboxylase	109	49.7	6.7	7.3	2.8
AT2G07734	ribosomal protein S4 (RPS4)	111	21.6	2.7	7.8	2.9
AT2G07827	unknown protein	113	11.8	1.4	8.4	3.0
AT2G07787	unknown protein	115	19.4	3.0	6.3	2.6
AT2G07775	unknown protein	119	8.6	1.2	6.8	2.7
AT2G07806	unknown protein	120	12.0	2.5	4.7	2.2
AT2G07759	pre-tRNA	123	64.4	1.8	34.0	5.0
AT2G07761	pre-tRNA	124	4.0	1.4	2.8	1.5
AT2G07762	pre-tRNA	125	29.1	1.4	20.1	4.3
AT2G07764	pre-tRNA	128	5.6	2.7	2.0	1.0
AT2G07835	unknown protein	130	7.5	1.6	4.7	2.2
AT2G07741	ATPase subunit 6, putative	131	39.9	4.8	8.2	3.0
AT2G07789	transposable element gene	139	4.0	1.0	3.7	1.9
