# Supplementary material for: Meiosis-specific gene discovery in plants: RNA-Seq applied to isolated Arabidopsis male meiocytes
Source: BMC Plant Biol. 2010 Dec 17;10:280. doi: 10.1186/1471-2229-10-280 (PMC3018465; doi:10.1186/1471-2229-10-280)
Supplement: Additional file 5 — Supplementary Table S3. Table S3. A list of differentially expressed TEs in meiocytes and anthers. The list of differentially expressed TEs in meiocytes and anthers, the label of "--" refers to zero (0) reads from anther. In addition to the mRNA signal intensity of read counts (normalized as reads per million reads), this table also provides gene ID, transposon ID, transposon family and super family. The shaded rows are genes that down-regulated in meiocytes and preferentially expressed in anthers. M = meiocyte, A = anther. [file 1471-2229-10-280-S5.PDF]

Supplemental Table S3. A list of differentiallly expressed TEs in meiocytes and anthers

Gene	M	A	M/A	Transposon	Transposon Family	Transposon Super Family
AT2G06980	2.44	0.70	3.47	AT2TE12570	ATDNA12T3A	DNA
AT4G05145	1.57	0.45	3.52	AT4TE12380	ATTIRX1D	DNA
AT4G06477	47.00	4.78	9.84	AT4TE14180	ATDNA12T3_2	DNA
AT1G30150	3.70	1.27	2.92	AT1TE34315	ATENSPM10	DNA/En-Spm
AT1G31570	1.73	0.39	4.47	AT1TE36570	ATENSPM3	DNA/En-Spm
AT1G33135	1.39	0.15	9.35	AT1TE38940	ATENSPM1A	DNA/En-Spm
AT1G35300	1.49	0.23	6.41	AT1TE42225	ATENSPM5	DNA/En-Spm
AT1G35590	1.12	0.15	7.56	AT1TE42890	ATENSPM5	DNA/En-Spm
AT1G35600	2.73	0.35	7.83	AT1TE42890	ATENSPM5	DNA/En-Spm
AT1G36190	1.47	0.39	3.80	AT1TE44450	ATENSPM4	DNA/En-Spm
AT1G36270	3.05	0.59	5.19	AT1TE44630	ATENSPM2	DNA/En-Spm
AT1G36460	1.35	0.38	3.53	AT1TE45020	ATENSPM5	DNA/En-Spm
AT1G36630	6.38	0.70	9.05	AT1TE45375	ATENSPM3	DNA/En-Spm
AT1G37040	1.59	0.45	3.56	AT1TE46235	ATENSPM9	DNA/En-Spm
AT1G39110	1.68	0.06	28.87	AT1TE48035	ATENSPM5	DNA/En-Spm
AT1G39270	2.93	0.21	14.18	AT1TE48035	ATENSPM5	DNA/En-Spm
AT1G40105	1.86	0.50	3.69	AT1TE49675	ATENSPM6	DNA/En-Spm
AT1G40107	1.09	0.54	2.03	AT1TE49740	ATENSPM7	DNA/En-Spm
AT1G40109	1.82	0.50	3.60	AT1TE49740	ATENSPM7	DNA/En-Spm
AT1G40123	1.03	0.00	--	AT1TE49955	ATENSPM5	DNA/En-Spm
AT1G42130	2.93	0.38	7.67	AT1TE51820	ATENSPM3	DNA/En-Spm
AT1G42150	2.08	0.59	3.51	AT1TE51820	ATENSPM3	DNA/En-Spm
AT1G42500	1.49	0.23	6.41	AT1TE52590	ATENSPM2	DNA/En-Spm
AT1G42510	3.15	0.63	5.03	AT1TE52590	ATENSPM2	DNA/En-Spm
AT1G43840	1.50	0.15	10.10	AT1TE54760	ATENSPM1A	DNA/En-Spm
AT1G52850	2.30	0.59	3.87	AT1TE65060	ATENSPM4	DNA/En-Spm
AT2G04210	1.44	0.24	6.03	AT2TE06620	ATENSPM6	DNA/En-Spm
AT2G05660	2.25	0.24	9.41	AT2TE09635	ATENSPM3	DNA/En-Spm
AT2G05750	1.49	0.30	5.02	AT2TE09955	ATENSPM2	DNA/En-Spm
AT2G06590	6.09	0.30	20.49	AT2TE11805	ATENSPM1A	DNA/En-Spm
AT2G06670	8.67	0.36	24.39	AT2TE11890	ATENSPM1A	DNA/En-Spm
AT2G06720	1.58	0.06	27.09	AT2TE11920	ATENSPM6	DNA/En-Spm
AT2G10070	1.62	0.53	3.06	AT2TE16420	ATENSPM9	DNA/En-Spm
AT2G10150	1.04	0.00	--	AT2TE16510	ATENSPM5	DNA/En-Spm
AT2G10490	1.18	0.15	7.95	AT2TE17110	ATENSPM6	DNA/En-Spm
AT2G10640	1.04	0.15	6.98	AT2TE17600	ATENSPM6	DNA/En-Spm
AT2G10650	2.14	0.74	2.88	AT2TE17600	ATENSPM6	DNA/En-Spm
AT2G11060	1.06	0.33	3.23	AT2TE18415	ATENSPM3	DNA/En-Spm
AT2G11550	2.61	0.86	3.03	AT2TE19200	ATENSPM7	DNA/En-Spm
AT2G11590	2.31	0.89	2.61	AT2TE19235	ATENSPM3	DNA/En-Spm
AT2G12210	2.51	0.57	4.41	AT2TE20205	ATENSPM3	DNA/En-Spm
AT2G12980	1.30	0.27	4.92	AT2TE21710	ATENSPM2	DNA/En-Spm
AT2G13000	3.31	1.65	2.00	AT2TE21710	ATENSPM2	DNA/En-Spm
AT2G13120	2.05	0.47	4.35	AT2TE21920	ATENSPM2	DNA/En-Spm
AT2G13160	1.44	0.27	5.43	AT2TE22040	ATENSPM1	DNA/En-Spm
AT2G13175	1.79	0.70	2.54	AT2TE22075	ATENSPM9	DNA/En-Spm
AT2G13310	1.31	0.09	14.52	AT2TE22335	ATENSPM1A	DNA/En-Spm
AT2G13750	1.11	0.30	3.75	AT2TE23185	ATENSPM9	DNA/En-Spm
AT2G13870	2.48	0.71	3.49	AT2TE23705	ATENSPM4	DNA/En-Spm

AT2G14970	2.45	0.78	3.16	AT2TE26360	ATENSPM7	DNA/En-Spm
AT2G14980	1.50	0.30	5.05	AT2TE26380	ATENSPM3	DNA/En-Spm
AT2G34130	1.74	0.86	2.02	AT2TE63800	ATENSPM1	DNA/En-Spm
AT3G30190	1.32	0.41	3.19	AT3TE49180	ATENSPM6	DNA/En-Spm
AT3G30330	1.15	0.57	2.02	AT3TE49700	ATENSPM4	DNA/En-Spm
AT3G30396	1.33	0.06	22.80	AT3TE50025	ATENSPM5	DNA/En-Spm
AT3G30670	1.47	0.09	16.32	AT3TE50675	ATENSPM5	DNA/En-Spm
AT3G30680	1.07	0.24	4.48	AT3TE50675	ATENSPM5	DNA/En-Spm
AT3G30721	1.40	0.21	6.76	AT3TE51140	ATENSPM11	DNA/En-Spm
AT3G30722	1.00	0.17	5.75	AT3TE51140	ATENSPM11	DNA/En-Spm
AT3G30743	2.01	0.48	4.20	AT3TE51250	ATENSPM9	DNA/En-Spm
AT3G30780	1.32	0.39	3.41	AT3TE51460	ATENSPM2	DNA/En-Spm
AT3G30790	4.85	1.50	3.23	AT3TE51460	ATENSPM2	DNA/En-Spm
AT3G30836	1.68	0.18	9.31	AT3TE51735	ATENSPM3	DNA/En-Spm
AT3G30837	1.95	0.15	13.10	AT3TE51735	ATENSPM3	DNA/En-Spm
AT3G31920	1.03	0.44	2.34	AT3TE53045	ATENSPM10	DNA/En-Spm
AT3G32226	1.54	0.56	2.73	AT3TE53985	ATENSPM2	DNA/En-Spm
AT3G32230	1.60	0.41	3.87	AT3TE53985	ATENSPM2	DNA/En-Spm
AT3G32677	1.13	0.00	--	AT3TE54690	ATENSPM5	DNA/En-Spm
AT3G32950	2.37	0.50	4.69	AT3TE54935	ATENSPM3	DNA/En-Spm
AT3G41345	1.23	0.00	--	AT3TE58090	ATENSPM6	DNA/En-Spm
AT3G42305	2.72	0.85	3.19	AT3TE59255	ATENSPM2	DNA/En-Spm
AT3G42650	1.36	0.45	3.00	AT3TE60280	ATENSPM4	DNA/En-Spm
AT3G42720	1.01	0.00	--	AT3TE60550	ATENSPM6	DNA/En-Spm
AT3G46487	1.09	0.39	2.81	AT3TE69350	ATENSPM9	DNA/En-Spm
AT4G02314	2.02	0.47	4.29	AT4TE05190	ATENSPM1	DNA/En-Spm
AT4G03910	1.62	0.17	9.28	AT4TE09085	ATENSPM5	DNA/En-Spm
AT4G04170	1.80	0.15	12.12	AT4TE09565	ATENSPM2	DNA/En-Spm
AT4G04270	1.65	0.36	4.63	AT4TE09780	ATENSPM9	DNA/En-Spm
AT4G04430	1.16	0.09	12.80	AT4TE10350	ATENSPM1	DNA/En-Spm
AT4G07518	1.72	0.50	3.40	AT4TE18435	ATENSPM6	DNA/En-Spm
AT4G07750	1.17	0.00	--	AT4TE19135	ATENSPM3	DNA/En-Spm
AT4G07760	2.03	0.12	17.45	AT4TE19135	ATENSPM3	DNA/En-Spm
AT4G08053	3.65	0.74	4.96	AT4TE20465	ATENSPM2	DNA/En-Spm
AT4G08060	2.24	0.50	4.44	AT4TE20535	ATENSPM7	DNA/En-Spm
AT4G08070	1.08	0.39	2.79	AT4TE20535	ATENSPM7	DNA/En-Spm
AT5G28165	2.46	0.12	21.12	AT5TE36940	ATENSPM5	DNA/En-Spm
AT5G28285	1.61	0.09	17.77	AT5TE37455	ATENSPM9	DNA/En-Spm
AT5G28526	3.01	1.22	2.46	AT5TE38340	ATENSPM3	DNA/En-Spm
AT5G28785	1.68	0.27	6.33	AT5TE39315	ATENSPM2	DNA/En-Spm
AT5G28923	1.28	0.06	21.95	AT5TE39830	ATENSPM5	DNA/En-Spm
AT5G29015	4.21	1.15	3.65	AT5TE40080	ATENSPM2	DNA/En-Spm
AT5G29408	1.06	0.09	11.69	AT5TE40425	ATENSPM1	DNA/En-Spm
AT5G30545	1.03	0.15	6.92	AT5TE42010	ATENSPM6	DNA/En-Spm
AT5G33234	3.53	1.27	2.79	AT5TE44240	ATENSPM6	DNA/En-Spm
AT5G34412	2.07	0.09	22.87	AT5TE45540	ATENSPM5	DNA/En-Spm
AT5G35116	1.07	0.30	3.60	AT5TE47400	ATENSPM1	DNA/En-Spm
AT5G35606	1.37	0.15	9.24	AT5TE49100	ATENSPM5	DNA/En-Spm
AT5G36650	1.30	0.09	14.42	AT5TE51685	ATENSPM1A	DNA/En-Spm
AT5G36655	1.00	0.15	6.76	AT5TE51685	ATENSPM1A	DNA/En-Spm
AT5G45082	2.00	0.12	17.14	AT5TE65540	ATENSPM5	DNA/En-Spm
AT5G59640	3.38	1.65	2.04	AT5TE86430	ATENSPM6	DNA/En-Spm

AT1G38185	8.30	1.93	4.30	AT1TE47100	HARBINGER	DNA/Harbinger
AT1G42580	0.31	1.51	0.20	AT1TE52715	ATIS112A	DNA/Harbinger
AT2G05025	1.97	0.56	3.55	AT2TE08160	ATIS112A	DNA/Harbinger
AT2G26630	1.49	0.27	5.51	AT2TE48990	HARBINGER	DNA/Harbinger
AT3G31915	1.40	0.30	4.70	AT3TE53010	ATIS112A	DNA/Harbinger
AT4G04635	1.17	0.48	2.46	AT4TE10925	HARBINGER	DNA/Harbinger
AT5G32621	1.08	0.41	2.61	AT5TE43580	ATIS112A	DNA/Harbinger
AT5G35205	6.40	1.92	3.33	AT5TE47735	ATIS112A	DNA/Harbinger
AT1G42110	1.48	0.21	7.17	AT1TE51770	TAG2	DNA/HAT
AT1G46192	1.58	0.15	10.62	AT1TE57040	TAG2	DNA/HAT
AT2G10410	73.53	193.18	0.38	AT2TE16940	ATHAT10	DNA/HAT
AT2G15940	1.59	0.57	2.79	AT2TE28280	TAG2	DNA/HAT
AT3G45270	1.16	0.15	7.79	AT3TE67325	ATHAT2	DNA/HAT
AT4G13120	1.58	7.17	0.22	AT4TE33430	ATHAT7	DNA/HAT
AT5G64685	8.35	3.79	2.21	AT5TE93050	TAG2	DNA/HAT
AT1G08735	3.81	0.68	5.56	AT1TE09080	VANDAL14	DNA/MuDR
AT1G08740	10.87	4.14	2.62	AT1TE09080	VANDAL14	DNA/MuDR
AT1G13660	2.98	0.45	6.70	AT1TE15170	AT9MU1	DNA/MuDR
AT1G17275	1.63	0.63	2.60	AT1TE19130	ATMU3	DNA/MuDR
AT1G21020	9.03	3.85	2.34	AT1TE23740	VANDAL14	DNA/MuDR
AT1G21040	3.81	0.68	5.56	AT1TE23740	VANDAL14	DNA/MuDR
AT1G24938	5.45	1.03	5.27	AT1TE28370	VANDAL5	DNA/MuDR
AT1G24967	7.25	1.67	4.35	AT1TE28370	VANDAL5	DNA/MuDR
AT1G25886	2.00	0.41	4.83	AT1TE29060	VANDAL2	DNA/MuDR
AT1G27780	2.17	0.47	4.60	AT1TE31190	VANDAL2	DNA/MuDR
AT1G29075	4.80	2.09	2.29	AT1TE32755	VANDAL1	DNA/MuDR
AT1G30980	3.01	1.16	2.59	AT1TE35680	VANDAL2N1	DNA/MuDR
AT1G34620	2.47	0.48	5.17	AT1TE41350	VANDAL16	DNA/MuDR
AT1G34740	2.37	0.68	3.49	AT1TE41460	VANDAL2	DNA/MuDR
AT1G35060	7.67	1.52	5.05	AT1TE41745	VANDAL5	DNA/MuDR
AT1G35080	2.86	0.83	3.43	AT1TE41745	VANDAL5	DNA/MuDR
AT1G35090	1.32	0.59	2.22	AT1TE41745	VANDAL5	DNA/MuDR
AT1G35612	3.35	6.74	0.50	AT1TE42900	VANDAL12	DNA/MuDR
AT1G35650	2.26	0.89	2.53	AT1TE43050	VANDAL14	DNA/MuDR
AT1G35760	9.72	3.88	2.51	AT1TE43355	VANDAL9	DNA/MuDR
AT1G35770	21.50	5.18	4.15	AT1TE43355	VANDAL9	DNA/MuDR
AT1G35995	1.06	0.00	--	AT1TE43880	ATMU11	DNA/MuDR
AT1G36440	4.36	0.80	5.44	AT1TE44980	VANDAL17	DNA/MuDR
AT1G37170	1.02	0.00	--	AT1TE46625	VANDAL8	DNA/MuDR
AT1G42450	3.18	0.58	5.47	AT1TE52385	VANDAL9	DNA/MuDR
AT1G42460	2.80	0.89	3.16	AT1TE52420	VANDAL11	DNA/MuDR
AT1G42590	1.80	0.39	4.65	AT1TE52735	VANDAL3	DNA/MuDR
AT1G42620	1.17	0.39	3.01	AT1TE52810	VANDAL4	DNA/MuDR
AT1G42650	2.57	0.50	5.10	AT1TE52825	VANDAL4	DNA/MuDR
AT1G43200	1.42	0.15	9.57	AT1TE53635	VANDAL8	DNA/MuDR
AT1G43220	1.05	0.15	7.09	AT1TE53660	VANDAL8	DNA/MuDR
AT1G43240	1.11	0.06	19.13	AT1TE53660	VANDAL8	DNA/MuDR
AT1G43280	1.08	0.21	5.22	AT1TE53765	VANDAL22	DNA/MuDR
AT1G43300	1.89	0.12	16.19	AT1TE53765	VANDAL22	DNA/MuDR
AT1G44840	2.85	0.71	4.01	AT1TE56135	VANDAL4	DNA/MuDR
AT1G44880	1.18	0.50	2.35	AT1TE56135	VANDAL4	DNA/MuDR
AT1G44935	3.20	1.30	2.46	AT1TE56230	BRODYAGA2	DNA/MuDR

AT1G45070	2.92	0.94	3.10	AT1TE56425	VANDAL1	DNA/MuDR
AT1G45090	1.72	0.62	2.76	AT1TE56425	VANDAL1	DNA/MuDR
AT1G47816	2.76	0.45	6.19	AT1TE58455	VANDAL18	DNA/MuDR
AT1G48250	1.14	0.48	2.39	AT1TE59130	ATMU3	DNA/MuDR
AT1G48290	3.92	0.30	13.19	AT1TE59235	ATMU7	DNA/MuDR
AT1G52020	1.26	0.06	21.64	AT1TE64080	VANDAL2	DNA/MuDR
AT1G52087	3.38	0.41	8.17	AT1TE64170	VANDAL5	DNA/MuDR
AT1G52610	1.59	0.50	3.15	AT1TE64825	ATMU1	DNA/MuDR
AT1G67240	1.97	0.00	--	AT1TE82600	ATMU2	DNA/MuDR
AT1G78095	2.73	1.16	2.36	AT1TE96100	ATMU11	DNA/MuDR
AT1G78350	2.81	1.31	2.15	AT1TE96425	ATMU3	DNA/MuDR
AT2G03990	1.99	0.09	21.99	AT2TE05755	VANDAL21	DNA/MuDR
AT2G04010	1.60	0.12	13.70	AT2TE05755	VANDAL21	DNA/MuDR
AT2G04310	1.79	0.12	15.38	AT2TE06955	VANDAL21	DNA/MuDR
AT2G04330	1.54	0.21	7.45	AT2TE06955	VANDAL21	DNA/MuDR
AT2G04990	1.74	0.15	11.73	AT2TE08135	VANDAL6	DNA/MuDR
AT2G05130	1.78	0.00	--	AT2TE08495	ATMU2	DNA/MuDR
AT2G05200	1.40	0.06	24.11	AT2TE08660	ATDNAI27T9C	DNA/MuDR
AT2G05490	2.67	1.18	2.26	AT2TE09205	VANDAL1	DNA/MuDR
AT2G05680	4.06	1.04	3.90	AT2TE09685	VANDAL3	DNA/MuDR
AT2G05690	1.57	0.23	6.73	AT2TE09685	VANDAL3	DNA/MuDR
AT2G05800	1.06	0.00	--	AT2TE10160	VANDAL18	DNA/MuDR
AT2G06120	2.37	0.24	9.91	AT2TE10965	VANDAL17	DNA/MuDR
AT2G06220	1.18	0.24	4.96	AT2TE11110	VANDAL7	DNA/MuDR
AT2G06600	7.36	0.86	8.57	AT2TE11815	VANDAL1	DNA/MuDR
AT2G06620	1.42	0.09	15.67	AT2TE11815	VANDAL1	DNA/MuDR
AT2G06680	11.45	1.31	8.77	AT2TE11900	VANDAL1	DNA/MuDR
AT2G06700	2.60	0.15	17.50	AT2TE11900	VANDAL1	DNA/MuDR
AT2G06710	1.64	0.36	4.60	AT2TE11900	VANDAL1	DNA/MuDR
AT2G06860	25.03	5.35	4.68	AT2TE12225	VANDAL9	DNA/MuDR
AT2G06870	4.88	0.89	5.51	AT2TE12225	VANDAL9	DNA/MuDR
AT2G07100	1.50	0.06	25.78	AT2TE12825	ATMU9	DNA/MuDR
AT2G07380	3.22	1.36	2.36	AT2TE13335	VANDAL3	DNA/MuDR
AT2G07395	4.93	1.45	3.40	AT2TE13335	VANDAL3	DNA/MuDR
AT2G07400	2.95	0.44	6.70	AT2TE13335	VANDAL3	DNA/MuDR
AT2G07580	1.64	0.68	2.41	AT2TE13750	VANDAL3	DNA/MuDR
AT2G07770	1.49	0.33	4.53	AT2TE15655	VANDAL17	DNA/MuDR
AT2G07780	2.28	0.47	4.83	AT2TE15655	VANDAL17	DNA/MuDR
AT2G10350	1.61	0.27	6.06	AT2TE16930	VANDAL4	DNA/MuDR
AT2G10400	2.22	0.59	3.74	AT2TE16930	VANDAL4	DNA/MuDR
AT2G11210	1.41	0.32	4.35	AT2TE18695	VANDAL20	DNA/MuDR
AT2G11480	1.68	0.36	4.65	AT2TE19070	VANDAL5	DNA/MuDR
AT2G11600	1.30	0.36	3.67	AT2TE19270	ATMU9	DNA/MuDR
AT2G11790	2.16	0.45	4.85	AT2TE19615	VANDAL21	DNA/MuDR
AT2G12060	1.45	0.09	16.04	AT2TE20140	VANDAL21	DNA/MuDR
AT2G12083	2.69	0.24	11.28	AT2TE20140	VANDAL21	DNA/MuDR
AT2G12100	1.67	0.68	2.46	AT2TE20160	VANDAL1	DNA/MuDR
AT2G12110	1.09	0.06	18.71	AT2TE20160	VANDAL1	DNA/MuDR
AT2G12150	2.79	1.00	2.78	AT2TE20160	VANDAL1	DNA/MuDR
AT2G12680	5.31	1.60	3.31	AT2TE21295	VANDAL3	DNA/MuDR
AT2G12700	1.28	0.27	4.82	AT2TE21295	VANDAL3	DNA/MuDR
AT2G12720	2.43	0.56	4.32	AT2TE21295	VANDAL3	DNA/MuDR

AT2G12730	1.08	0.09	11.96	AT2TE21335	AT9MU1	DNA/MuDR
AT2G13975	4.21	14.07	0.30	AT2TE23870	ATMU10	DNA/MuDR
AT2G14320	1.77	4.38	0.40	AT2TE24735	VANDAL7	DNA/MuDR
AT2G14330	0.40	1.31	0.31	AT2TE24735	VANDAL7	DNA/MuDR
AT2G14350	2.99	0.50	5.94	AT2TE24735	VANDAL7	DNA/MuDR
AT2G14380	1.93	0.00	--	AT2TE24845	ATDNAI27T9C	DNA/MuDR
AT2G14590	2.67	0.36	7.50	AT2TE25395	VANDAL12	DNA/MuDR
AT2G14595	3.42	1.00	3.41	AT2TE25400	VANDAL9	DNA/MuDR
AT2G14770	1.01	0.44	2.30	AT2TE25840	VANDAL2	DNA/MuDR
AT2G16150	2.99	1.00	3.00	AT2TE28565	VANDAL1	DNA/MuDR
AT2G16180	1.55	0.59	2.64	AT2TE28565	VANDAL1	DNA/MuDR
AT2G16832	2.30	0.23	9.86	AT2TE29730	VANDAL2N1	DNA/MuDR
AT2G17460	2.73	1.04	2.63	AT2TE31215	VANDAL2N1	DNA/MuDR
AT2G23480	2.04	0.30	6.86	AT2TE42810	VANDAL21	DNA/MuDR
AT2G23500	2.56	0.21	12.39	AT2TE42810	VANDAL21	DNA/MuDR
AT2G24890	2.62	0.56	4.65	AT2TE45465	VANDAL3	DNA/MuDR
AT2G24910	1.23	0.27	4.63	AT2TE45465	VANDAL3	DNA/MuDR
AT2G24930	2.98	0.77	3.87	AT2TE45465	VANDAL3	DNA/MuDR
AT2G29230	4.03	0.74	5.43	AT2TE54910	VANDAL14	DNA/MuDR
AT2G29240	10.58	4.26	2.48	AT2TE54910	VANDAL14	DNA/MuDR
AT3G09165	2.47	0.48	5.17	AT3TE11850	VANDAL16	DNA/MuDR
AT3G15602	1.22	0.47	2.58	AT3TE22200	VANDAL17	DNA/MuDR
AT3G17260	15.15	6.90	2.20	AT3TE24790	ARNOLD2	DNA/MuDR
AT3G24390	2.05	0.32	6.34	AT3TE37060	VANDAL2	DNA/MuDR
AT3G26525	3.81	0.68	5.56	AT3TE40530	VANDAL14	DNA/MuDR
AT3G26530	10.87	4.14	2.62	AT3TE40530	VANDAL14	DNA/MuDR
AT3G29612	1.53	0.06	26.25	AT3TE47555	VANDAL21	DNA/MuDR
AT3G29618	1.12	0.12	9.59	AT3TE47645	VANDAL17	DNA/MuDR
AT3G29642	3.79	1.09	3.49	AT3TE47905	VANDAL3	DNA/MuDR
AT3G29695	2.44	0.21	11.82	AT3TE48070	VANDAL21	DNA/MuDR
AT3G29710	2.73	0.15	18.34	AT3TE48070	VANDAL21	DNA/MuDR
AT3G29777	2.47	0.89	2.77	AT3TE48465	VANDAL2N1	DNA/MuDR
AT3G30170	2.21	0.30	7.45	AT3TE49090	ATMU5	DNA/MuDR
AT3G30465	3.75	0.30	12.61	AT3TE50375	VANDAL3	DNA/MuDR
AT3G30490	1.10	0.27	4.14	AT3TE50375	VANDAL3	DNA/MuDR
AT3G30763	1.79	0.81	2.22	AT3TE51350	VANDAL3	DNA/MuDR
AT3G31410	1.03	0.21	4.97	AT3TE52540	VANDAL21	DNA/MuDR
AT3G31450	3.38	1.10	3.08	AT3TE52665	VANDAL21	DNA/MuDR
AT3G32060	1.37	0.24	5.75	AT3TE53580	VANDAL21	DNA/MuDR
AT3G32080	1.42	0.00	--	AT3TE53580	VANDAL21	DNA/MuDR
AT3G32393	1.15	0.06	19.71	AT3TE54510	VANDAL2	DNA/MuDR
AT3G32425	2.73	0.30	9.17	AT3TE54575	ATMU9	DNA/MuDR
AT3G32475	1.35	0.00	--	AT3TE54670	VANDAL4	DNA/MuDR
AT3G32914	2.79	0.09	30.87	AT3TE54820	ARNOLD2	DNA/MuDR
AT3G32966	2.57	0.65	3.94	AT3TE55005	VANDAL3	DNA/MuDR
AT3G32968	1.88	0.12	16.14	AT3TE55010	VANDAL3	DNA/MuDR
AT3G33005	2.08	0.09	22.97	AT3TE55140	VANDAL2	DNA/MuDR
AT3G33009	1.48	0.12	12.73	AT3TE55160	VANDAL2	DNA/MuDR
AT3G33225	1.50	0.30	5.05	AT3TE57780	VANDAL7	DNA/MuDR
AT3G33235	2.91	6.22	0.47	AT3TE57780	VANDAL7	DNA/MuDR
AT3G33377	0.86	7.61	0.11	AT3TE57780	VANDAL7	DNA/MuDR
AT3G33448	1.64	5.74	0.29	AT3TE57780	VANDAL7	DNA/MuDR

AT3G34299	0.72	5.84	0.12	AT3TE57935	VANDAL7	DNA/MuDR
AT3G42478	1.82	0.39	4.69	AT3TE59865	VANDAL4	DNA/MuDR
AT3G42530	1.00	0.23	4.31	AT3TE59865	VANDAL4	DNA/MuDR
AT3G42690	1.40	0.68	2.04	AT3TE60385	VANDAL20	DNA/MuDR
AT3G42794	1.03	0.17	5.89	AT3TE60750	ATMU3	DNA/MuDR
AT3G42820	18.24	7.20	2.53	AT3TE60875	VANDAL12	DNA/MuDR
AT3G42900	1.04	0.17	5.94	AT3TE61025	VANDAL9	DNA/MuDR
AT3G42910	1.83	0.15	12.34	AT3TE61035	VANDAL11	DNA/MuDR
AT3G42945	3.81	0.69	5.51	AT3TE61205	VANDAL15	DNA/MuDR
AT3G43080	1.72	0.45	3.87	AT3TE61420	VANDAL4	DNA/MuDR
AT3G43360	1.35	0.36	3.79	AT3TE62260	VANDAL2	DNA/MuDR
AT3G43370	1.32	0.51	2.59	AT3TE62260	VANDAL2	DNA/MuDR
AT3G43390	1.53	0.09	16.89	AT3TE62260	VANDAL2	DNA/MuDR
AT3G43526	1.16	0.15	7.79	AT3TE62595	VANDAL17	DNA/MuDR
AT3G43530	4.75	0.92	5.18	AT3TE62595	VANDAL17	DNA/MuDR
AT3G43730	1.55	0.41	3.75	AT3TE63205	VANDAL8	DNA/MuDR
AT3G43780	2.16	0.80	2.70	AT3TE63285	VANDAL5	DNA/MuDR
AT3G44470	1.13	0.06	19.44	AT3TE65100	ARNOLD1	DNA/MuDR
AT3G45340	1.18	4.91	0.24	AT3TE67445	VANDAL7	DNA/MuDR
AT3G45350	0.18	1.27	0.14	AT3TE67445	VANDAL7	DNA/MuDR
AT3G45380	1.95	0.39	5.02	AT3TE67445	VANDAL7	DNA/MuDR
AT3G59860	1.73	0.30	5.83	AT3TE90030	VANDAL2N1	DNA/MuDR
AT4G03970	1.12	0.54	2.08	AT4TE09295	VANDAL4	DNA/MuDR
AT4G03981	2.12	0.54	3.95	AT4TE09295	VANDAL4	DNA/MuDR
AT4G04130	2.55	0.92	2.77	AT4TE09480	VANDAL20	DNA/MuDR
AT4G04393	1.05	3.82	0.27	AT4TE10210	VANDAL7	DNA/MuDR
AT4G04395	0.45	1.12	0.40	AT4TE10210	VANDAL7	DNA/MuDR
AT4G04400	1.58	0.39	4.07	AT4TE10210	VANDAL7	DNA/MuDR
AT4G04530	1.84	0.41	4.44	AT4TE10490	VANDAL18	DNA/MuDR
AT4G05280	1.19	0.00	--	AT4TE12615	VANDAL2	DNA/MuDR
AT4G05585	3.06	0.51	6.01	AT4TE13435	VANDAL2	DNA/MuDR
AT4G06579	2.22	0.33	6.75	AT4TE15955	VANDAL3	DNA/MuDR
AT4G07520	1.45	0.06	24.84	AT4TE18470	VANDAL8	DNA/MuDR
AT4G07680	6.02	1.45	4.16	AT4TE18865	VANDAL3	DNA/MuDR
AT4G07693	2.48	0.74	3.34	AT4TE18865	VANDAL3	DNA/MuDR
AT4G07696	2.17	0.54	4.05	AT4TE18875	VANDAL4	DNA/MuDR
AT4G08220	2.01	16.10	0.13	AT4TE21570	VANDAL17	DNA/MuDR
AT4G08340	25.84	9.14	2.83	AT4TE21900	VANDAL11	DNA/MuDR
AT4G08600	1.33	0.09	14.70	AT4TE22970	VANDAL8	DNA/MuDR
AT4G08650	2.08	0.54	3.89	AT4TE23125	VANDAL3	DNA/MuDR
AT4G08660	2.39	0.74	3.25	AT4TE23125	VANDAL3	DNA/MuDR
AT4G08680	2.73	1.21	2.25	AT4TE23190	ATMU3	DNA/MuDR
AT4G08710	1.64	0.41	3.96	AT4TE23340	VANDAL21	DNA/MuDR
AT4G08720	2.33	0.92	2.52	AT4TE23345	VANDAL21	DNA/MuDR
AT4G08880	2.11	0.38	5.53	AT4TE23825	VANDAL2	DNA/MuDR
AT4G08890	1.06	0.30	3.58	AT4TE23825	VANDAL2	DNA/MuDR
AT4G09290	1.90	0.50	3.78	AT4TE24825	VANDAL3	DNA/MuDR
AT4G09400	0.54	1.42	0.38	AT4TE25065	VANDAL7	DNA/MuDR
AT4G09410	1.13	3.05	0.37	AT4TE25065	VANDAL7	DNA/MuDR
AT4G19280	2.64	0.65	4.05	AT4TE48195	VANDAL5	DNA/MuDR
AT4G19300	1.46	0.59	2.45	AT4TE48215	VANDAL5	DNA/MuDR
AT4G19310	5.32	1.90	2.80	AT4TE48230	VANDAL5	DNA/MuDR

AT4G19320	4.65	0.74	6.31	AT4TE48230	VANDAL5	DNA/MuDR
AT5G03950	1.84	0.68	2.72	AT5TE03885	VANDAL2N1	DNA/MuDR
AT5G20750	1.85	4.91	0.38	AT5TE25460	VANDAL20	DNA/MuDR
AT5G26345	2.74	0.36	7.72	AT5TE33455	ATMU1	DNA/MuDR
AT5G26350	1.59	0.44	3.61	AT5TE33455	ATMU1	DNA/MuDR
AT5G27160	2.20	0.32	6.82	AT5TE34730	VANDAL8	DNA/MuDR
AT5G27190	1.86	0.09	20.57	AT5TE34730	VANDAL8	DNA/MuDR
AT5G27500	1.22	0.50	2.45	AT5TE35230	ATMU7	DNA/MuDR
AT5G27505	3.66	0.65	5.66	AT5TE35265	ATMU5	DNA/MuDR
AT5G27902	1.04	0.09	11.48	AT5TE36140	ATMU11	DNA/MuDR
AT5G28170	1.88	0.56	3.37	AT5TE36975	VANDAL5A	DNA/MuDR
AT5G28173	7.60	1.79	4.25	AT5TE36975	VANDAL5A	DNA/MuDR
AT5G28200	2.90	0.83	3.50	AT5TE37085	VANDAL12	DNA/MuDR
AT5G28250	1.78	0.54	3.32	AT5TE37270	VANDAL14	DNA/MuDR
AT5G28253	1.09	0.17	6.24	AT5TE37290	VANDAL11	DNA/MuDR
AT5G28263	6.75	0.83	8.16	AT5TE37350	VANDAL1	DNA/MuDR
AT5G28480	2.21	0.62	3.57	AT5TE38070	VANDAL1	DNA/MuDR
AT5G28484	1.08	0.12	9.28	AT5TE38070	VANDAL1	DNA/MuDR
AT5G28487	3.74	1.15	3.25	AT5TE38070	VANDAL1	DNA/MuDR
AT5G28600	22.87	11.18	2.05	AT5TE38675	VANDAL9	DNA/MuDR
AT5G28605	3.62	1.45	2.50	AT5TE38675	VANDAL9	DNA/MuDR
AT5G28760	1.15	0.21	5.56	AT5TE39230	VANDAL8	DNA/MuDR
AT5G28970	10.00	2.70	3.70	AT5TE40000	VANDAL5	DNA/MuDR
AT5G28980	1.60	0.59	2.69	AT5TE40000	VANDAL5	DNA/MuDR
AT5G28993	6.53	1.82	3.60	AT5TE40000	VANDAL5	DNA/MuDR
AT5G33220	1.54	0.38	4.05	AT5TE44200	VANDAL6	DNA/MuDR
AT5G33402	2.18	0.47	4.62	AT5TE44880	VANDAL3	DNA/MuDR
AT5G35025	2.89	1.01	2.87	AT5TE47130	VANDAL3	DNA/MuDR
AT5G35035	1.05	0.38	2.74	AT5TE47130	VANDAL3	DNA/MuDR
AT5G35045	1.34	0.33	4.06	AT5TE47130	VANDAL3	DNA/MuDR
AT5G35420	0.78	1.80	0.43	AT5TE48590	VANDAL16	DNA/MuDR
AT5G35575	1.13	0.15	7.62	AT5TE48980	ATMU11	DNA/MuDR
AT5G35643	1.17	0.27	4.43	AT5TE49325	VANDAL2	DNA/MuDR
AT5G35791	1.70	0.15	11.42	AT5TE49755	VANDAL21	DNA/MuDR
AT5G35794	3.67	0.36	10.32	AT5TE49755	VANDAL21	DNA/MuDR
AT5G36020	4.50	0.59	7.65	AT5TE50560	VANDAL5	DNA/MuDR
AT5G36030	4.63	1.63	2.83	AT5TE50560	VANDAL5	DNA/MuDR
AT5G36040	1.04	0.39	2.68	AT5TE50560	VANDAL5	DNA/MuDR
AT5G36060	1.02	0.21	4.93	AT5TE50560	VANDAL5	DNA/MuDR
AT5G36070	1.39	0.57	2.45	AT5TE50590	VANDAL4	DNA/MuDR
AT5G36075	7.48	2.35	3.18	AT5TE50620	VANDAL5	DNA/MuDR
AT5G36860	2.13	0.47	4.51	AT5TE52200	VANDAL2	DNA/MuDR
AT5G37330	1.09	0.41	2.63	AT5TE53310	VANDAL3	DNA/MuDR
AT5G38870	8.03	22.90	0.35	AT5TE56250	ATMU10	DNA/MuDR
AT5G40110	1.53	0.65	2.34	AT5TE57960	VANDAL2N1	DNA/MuDR
AT5G43015	3.51	0.54	6.55	AT5TE62345	AT9NMU1	DNA/MuDR
AT5G44875	3.81	0.68	5.56	AT5TE65330	VANDAL14	DNA/MuDR
AT5G44890	10.87	4.14	2.62	AT5TE65330	VANDAL14	DNA/MuDR
AT5G45576	1.33	0.59	2.23	AT5TE66500	VANDAL3	DNA/MuDR
AT5G56367	3.49	0.45	7.83	AT5TE82140	AT9MU1	DNA/MuDR
AT1G02228	1.21	0.59	2.05	AT1TE01405	ATLINE1A	LINE/L1
AT1G17390	5.42	1.72	3.14	AT1TE19270	ATLINE1_2	LINE/L1

AT1G22560	1.64	0.53	3.09	AT1TE25645	TA11	LINE/L1
AT1G23990	3.35	0.67	4.99	AT1TE27405	ATLINEIII	LINE/L1
AT1G26860	1.47	0.41	3.54	AT1TE29990	ATLINE1A	LINE/L1
AT1G29650	3.47	0.80	4.37	AT1TE33490	TA11	LINE/L1
AT1G30030	3.05	0.89	3.42	AT1TE34040	ATLINE1_5	LINE/L1
AT1G31030	3.86	1.75	2.21	AT1TE35720	ATLINEIII	LINE/L1
AT1G31100	3.01	0.36	8.47	AT1TE35855	ATLINE2	LINE/L1
AT1G34842	0.76	3.55	0.22	AT1TE41560	ATLINE1_6	LINE/L1
AT1G35146	2.49	0.63	3.97	AT1TE41860	TA11	LINE/L1
AT1G35186	2.98	0.27	11.01	AT1TE41940	ATLINE1_5	LINE/L1
AT1G35390	1.71	0.21	8.25	AT1TE42410	TA11	LINE/L1
AT1G35960	1.65	0.15	11.07	AT1TE43780	TA11	LINE/L1
AT1G36010	3.23	0.94	3.42	AT1TE43910	ATLINE1_1	LINE/L1
AT1G36360	2.35	0.57	4.14	AT1TE44820	TA11	LINE/L1
AT1G37057	1.23	0.39	3.17	AT1TE46270	ATLINEIII	LINE/L1
AT1G43270	3.29	0.83	3.98	AT1TE53730	ATLINE1_6	LINE/L1
AT1G43570	1.90	0.83	2.29	AT1TE54030	ATLINEIII	LINE/L1
AT1G43960	2.02	0.30	6.81	AT1TE54975	ATLINE1_2	LINE/L1
AT1G45140	1.10	0.45	2.48	AT1TE56505	ATLINE1A	LINE/L1
AT1G47606	1.20	0.17	6.87	AT1TE58005	ATLINE1_2	LINE/L1
AT1G47910	2.82	0.97	2.91	AT1TE58625	ATLINE1A	LINE/L1
AT1G51750	1.65	0.09	18.24	AT1TE63610	ATLINEIII	LINE/L1
AT1G58020	12.96	2.25	5.76	AT1TE70805	ATLINE1_1	LINE/L1
AT1G62695	6.69	1.29	5.18	AT1TE76520	ATLINE1_1	LINE/L1
AT1G65750	1.17	0.27	4.43	AT1TE80320	ATLINE1_1	LINE/L1
AT1G67626	6.89	2.79	2.48	AT1TE83175	ATLINE1_1	LINE/L1
AT2G01550	2.20	0.32	6.82	AT2TE01000	ATLINEIII	LINE/L1
AT2G02260	1.60	0.41	3.86	AT2TE02530	ATLINEIII	LINE/L1
AT2G03940	1.27	0.17	7.27	AT2TE05535	ATLINE2	LINE/L1
AT2G04180	2.74	0.42	6.54	AT2TE06530	ATLINE1_4	LINE/L1
AT2G04200	1.73	0.36	4.88	AT2TE06590	TA11	LINE/L1
AT2G05110	1.71	0.74	2.30	AT2TE08375	TA11	LINE/L1
AT2G05550	1.22	0.00	--	AT2TE09360	ATLINEIII	LINE/L1
AT2G06400	2.33	0.44	5.30	AT2TE11435	ATLINE1_5	LINE/L1
AT2G06580	4.29	1.09	3.93	AT2TE11770	ATLINE1_6	LINE/L1
AT2G06650	3.52	1.03	3.41	AT2TE11820	ATLINE1_6	LINE/L1
AT2G07150	1.80	0.47	3.82	AT2TE12910	ATLINE1A	LINE/L1
AT2G07160	5.54	2.43	2.28	AT2TE12955	ATLINE1_5	LINE/L1
AT2G07570	2.35	0.50	4.66	AT2TE13735	TA11	LINE/L1
AT2G07650	2.04	0.15	13.74	AT2TE13950	ATLINE1_2	LINE/L1
AT2G07730	3.33	0.68	4.91	AT2TE15565	ATLINE1_2	LINE/L1
AT2G07740	2.43	0.06	41.66	AT2TE15565	ATLINE1_2	LINE/L1
AT2G07767	1.81	0.27	6.68	AT2TE14150	ATLINEIII	LINE/L1
AT2G07784	1.37	0.39	3.54	AT2TE14370	TA11	LINE/L1
AT2G09860	1.58	0.06	27.09	AT2TE16160	ATLINE2	LINE/L1
AT2G10910	1.14	0.06	19.60	AT2TE18030	ATLINE1_4	LINE/L1
AT2G11240	43.51	204.08	0.21	AT2TE18800	ATLINE1_6	LINE/L1
AT2G11800	2.25	1.12	2.01	AT2TE19650	ATLINE1A	LINE/L1
AT2G12195	3.81	0.50	7.64	AT2TE20185	TA11	LINE/L1
AT2G12770	3.92	0.92	4.24	AT2TE21380	ATLINE1_6	LINE/L1
AT2G13300	4.15	1.86	2.23	AT2TE22315	ATLINE1_6	LINE/L1
AT2G13460	1.75	0.27	6.61	AT2TE22630	TA11	LINE/L1

AT2G13470	1.13	0.15	7.62	AT2TE22720	TA11	LINE/L1
AT2G13520	1.32	0.36	3.72	AT2TE22740	TA11	LINE/L1
AT2G14430	1.83	0.83	2.22	AT2TE24950	ATLINE2	LINE/L1
AT2G14990	4.80	1.07	4.50	AT2TE26470	ATLINE1_4	LINE/L1
AT2G15250	2.66	0.50	5.27	AT2TE26980	TA11	LINE/L1
AT2G15540	1.26	0.36	3.55	AT2TE27670	ATLINE1_4	LINE/L1
AT2G16420	3.91	0.44	8.89	AT2TE28955	ATLINE1_5	LINE/L1
AT2G18820	2.95	0.83	3.57	AT2TE33890	ATLINE2	LINE/L1
AT2G22210	3.50	0.56	6.22	AT2TE40160	ATLINE1_4	LINE/L1
AT2G22350	1.58	0.06	27.20	AT2TE40405	ATLINE1_1	LINE/L1
AT2G23880	5.49	1.12	4.88	AT2TE43485	ATLINEIII	LINE/L1
AT2G25550	10.31	2.67	3.86	AT2TE46755	ATLINE1_4	LINE/L1
AT2G28750	1.89	0.36	5.31	AT2TE53790	ATLINE1_4	LINE/L1
AT2G28980	1.86	0.47	3.94	AT2TE54360	ATLINEIII	LINE/L1
AT2G36402	1.31	0.36	3.69	AT2TE68040	ATLINE2	LINE/L1
AT2G41745	2.55	0.68	3.75	AT2TE78600	ATLINE1A	LINE/L1
AT3G05415	4.70	2.02	2.33	AT3TE06550	ATLINE2	LINE/L1
AT3G24675	1.54	0.44	3.49	AT3TE37690	TA11	LINE/L1
AT3G25815	2.38	0.56	4.23	AT3TE39395	ATLINE2	LINE/L1
AT3G25826	2.77	11.04	0.25	AT3TE39430	ATLINEIII	LINE/L1
AT3G26614	1.45	0.59	2.44	AT3TE40730	ATLINEIII	LINE/L1
AT3G28705	1.84	0.68	2.72	AT3TE44790	TA11	LINE/L1
AT3G28865	13.08	2.69	4.85	AT3TE45240	ATLINE1_3A	LINE/L1
AT3G28915	5.28	1.36	3.87	AT3TE45385	ATLINE1_6	LINE/L1
AT3G28945	2.65	0.87	3.06	AT3TE45620	TA11	LINE/L1
AT3G29078	1.34	0.36	3.70	AT3TE45995	ATLINE2	LINE/L1
AT3G29778	2.18	0.59	3.67	AT3TE48455	ATLINEIII	LINE/L1
AT3G30183	1.29	0.30	4.35	AT3TE49170	ATLINE1A	LINE/L1
AT3G30570	2.14	0.71	3.01	AT3TE50505	ATLINE1_2	LINE/L1
AT3G31317	2.29	0.42	5.45	AT3TE52190	TA11	LINE/L1
AT3G31630	2.93	0.54	5.47	AT3TE52800	ATLINE1_6	LINE/L1
AT3G32110	2.67	0.54	4.99	AT3TE53630	ATLINE1_1	LINE/L1
AT3G33565	1.58	0.41	3.81	AT3TE57650	TA11	LINE/L1
AT3G42545	8.37	2.86	2.92	AT3TE59985	ATLINE1_6	LINE/L1
AT3G42803	2.15	0.39	5.54	AT3TE60830	ATLINE1_5	LINE/L1
AT3G43175	1.90	0.06	32.54	AT3TE61685	ATLINE1_2	LINE/L1
AT3G43315	1.40	0.68	2.06	AT3TE62085	ATLINEIII	LINE/L1
AT3G43357	1.57	0.06	26.94	AT3TE62220	ATLINEIII	LINE/L1
AT3G43546	2.00	0.99	2.02	AT3TE62655	TA11	LINE/L1
AT3G43573	1.64	0.47	3.47	AT3TE62765	TA11	LINE/L1
AT3G43575	1.36	0.39	3.50	AT3TE62785	ATLINE1_6	LINE/L1
AT3G43625	2.81	0.41	6.80	AT3TE62915	TA11	LINE/L1
AT3G43715	1.61	0.47	3.41	AT3TE63085	ATLINE1_6	LINE/L1
AT3G43760	1.09	0.15	7.33	AT3TE63270	ATLINEIII	LINE/L1
AT3G43835	2.84	0.77	3.69	AT3TE63470	TA11	LINE/L1
AT3G44096	1.93	0.62	3.11	AT3TE64160	ATLINE2	LINE/L1
AT3G44175	2.15	0.68	3.17	AT3TE64355	ATLINE1A	LINE/L1
AT3G44425	2.89	1.29	2.23	AT3TE64985	ATLINEIII	LINE/L1
AT3G44650	1.90	0.89	2.14	AT3TE65655	ATLINEIII	LINE/L1
AT3G45253	24.13	7.50	3.22	AT3TE67295	ATLINE1_1	LINE/L1
AT3G45256	1.21	0.48	2.53	AT3TE67310	ATLINEIII	LINE/L1
AT3G47875	1.14	0.50	2.26	AT3TE71565	ATLINE1_4	LINE/L1

AT3G57586	1.84	0.48	3.86	AT3TE86800	TA11	LINE/L1
AT4G03920	1.48	0.68	2.17	AT4TE09130	ATLINEIII	LINE/L1
AT4G04000	2.02	0.95	2.12	AT4TE09315	ATLINE1_6	LINE/L1
AT4G07516	5.63	0.70	7.99	AT4TE18420	ATLINE1_1	LINE/L1
AT4G08262	8.13	3.77	2.16	AT4TE21695	ATLINE1_6	LINE/L1
AT4G08820	1.53	0.21	7.38	AT4TE23575	ATLINE1_1	LINE/L1
AT4G08830	2.12	0.47	4.49	AT4TE23575	ATLINE1_1	LINE/L1
AT4G09710	2.21	0.98	2.26	AT4TE25865	ATLINE1_6	LINE/L1
AT4G10830	4.25	1.40	3.04	AT4TE28510	ATLINE1_4	LINE/L1
AT4G15590	6.50	0.68	9.57	AT4TE39815	ATLINE1_2	LINE/L1
AT4G20725	8.68	2.60	3.33	AT4TE50995	ATLINE1_1	LINE/L1
AT5G04235	4.04	10.75	0.38	AT5TE04240	TA11	LINE/L1
AT5G06805	1.19	0.12	10.22	AT5TE07650	ATLINE1_2	LINE/L1
AT5G07505	4.85	0.71	6.83	AT5TE08630	ATLINE1_1	LINE/L1
AT5G10850	1.67	4.30	0.39	AT5TE12450	ATLINE1_1	LINE/L1
AT5G13475	2.23	0.36	6.27	AT5TE15640	ATLINE2	LINE/L1
AT5G17725	1.76	0.65	2.72	AT5TE21155	ATLINE1_5	LINE/L1
AT5G18633	1.79	0.30	6.01	AT5TE22490	TA11	LINE/L1
AT5G24915	6.21	2.84	2.19	AT5TE31020	ATLINE1_6	LINE/L1
AT5G25205	2.36	0.56	4.20	AT5TE31540	TA11	LINE/L1
AT5G26775	6.30	1.49	4.24	AT5TE34170	ATLINE1_6	LINE/L1
AT5G27905	2.84	0.23	12.17	AT5TE36155	ATLINEIII	LINE/L1
AT5G28053	2.92	0.62	4.70	AT5TE36620	ATLINE1_6	LINE/L1
AT5G28641	1.23	0.15	8.26	AT5TE38810	ATLINE1_4	LINE/L1
AT5G28773	5.46	1.57	3.48	AT5TE39270	ATLINE1_6	LINE/L1
AT5G28913	6.03	12.82	0.47	AT5TE39790	ATLINE1A	LINE/L1
AT5G32616	1.27	0.48	2.66	AT5TE43575	ATLINE2	LINE/L1
AT5G33360	5.68	0.97	5.86	AT5TE44640	ATLINE1_1	LINE/L1
AT5G35076	3.65	0.95	3.85	AT5TE47280	TA11	LINE/L1
AT5G35331	1.95	0.50	3.86	AT5TE47990	TA11	LINE/L1
AT5G35413	3.27	0.98	3.33	AT5TE48530	ATLINE1_5	LINE/L1
AT5G35416	4.02	1.06	3.79	AT5TE48550	ATLINE1_2	LINE/L1
AT5G35495	1.48	0.63	2.37	AT5TE48740	ATLINE1_4	LINE/L1
AT5G35535	3.74	0.62	6.03	AT5TE48820	ATLINE1_6	LINE/L1
AT5G35720	2.53	0.98	2.60	AT5TE49480	ATLINEIII	LINE/L1
AT5G35725	7.34	2.09	3.52	AT5TE49485	ATLINEIII	LINE/L1
AT5G35756	1.76	0.36	4.86	AT5TE49650	ATLINE1_2	LINE/L1
AT5G35802	1.10	0.06	18.86	AT5TE49820	ATLINEIII	LINE/L1
AT5G36005	1.22	0.32	3.77	AT5TE50485	TA11	LINE/L1
AT5G36905	6.47	1.33	4.88	AT5TE52345	ATLINE1_2	LINE/L1
AT5G36935	10.10	2.73	3.70	AT5TE52400	ATLINE1_2	LINE/L1
AT5G38285	4.90	1.93	2.54	AT5TE55355	ATLINE1_5	LINE/L1
AT5G39245	2.78	1.01	2.74	AT5TE56780	ATLINEIII	LINE/L1
AT5G39862	3.12	0.65	4.78	AT5TE57635	ATLINEIII	LINE/L1
AT5G41755	1.07	0.36	3.01	AT5TE60290	ATLINE1_6	LINE/L1
AT5G43065	9.09	3.90	2.33	AT5TE62455	ATLINEIII	LINE/L1
AT5G43105	13.30	3.67	3.62	AT5TE62545	ATLINE1_1	LINE/L1
AT5G43415	3.37	0.61	5.49	AT5TE63010	ATLINE1_6	LINE/L1
AT5G49465	1.29	0.56	2.30	AT5TE72235	TA11	LINE/L1
AT5G52065	1.80	0.06	30.92	AT5TE76190	ATLINE2	LINE/L1
AT5G53775	7.42	2.90	2.56	AT5TE78640	TA11	LINE/L1
AT1G21945	22.40	0.68	33.00	AT1TE24850	ATCOPIA78	LTR/Copia

AT1G26990	1.31	0.21	6.34	AT1TE30195	ATCOPIA16	LTR/Copia
AT1G27285	2.29	5.75	0.40	AT1TE30550	ATCOPIA3	LTR/Copia
AT1G30340	1.04	0.06	17.82	AT1TE34580	ATCOPIA73	LTR/Copia
AT1G31210	9.88	4.62	2.14	AT1TE36030	ATCOPIA25	LTR/Copia
AT1G32590	2.75	0.59	4.68	AT1TE38210	ATCOPIA31	LTR/Copia
AT1G34545	1.00	0.23	4.31	AT1TE41280	ATCOPIA15	LTR/Copia
AT1G34904	2.83	0.57	4.98	AT1TE41570	ATCOPIA93	LTR/Copia
AT1G34967	4.90	1.76	2.79	AT1TE41580	ATCOPIA93	LTR/Copia
AT1G35115	6.53	35.67	0.18	AT1TE41800	ATCOPIA36	LTR/Copia
AT1G35535	1.46	0.15	9.82	AT1TE42800	ATCOPIA69	LTR/Copia
AT1G35840	1.62	0.74	2.20	AT1TE43585	ATCOPIA84	LTR/Copia
AT1G36035	3.46	1.00	3.45	AT1TE43975	ATCOPIA43	LTR/Copia
AT1G36600	1.26	0.00	--	AT1TE45330	ATCOPIA41	LTR/Copia
AT1G42350	1.50	0.18	8.31	AT1TE52070	ATCOPIA49	LTR/Copia
AT1G42924	2.40	0.67	3.56	AT1TE53090	ENDOVIR1	LTR/Copia
AT1G43715	1.33	0.12	11.42	AT1TE54400	ATCOPIA48	LTR/Copia
AT1G43785	1.07	0.33	3.25	AT1TE54625	ATCOPIA35	LTR/Copia
AT1G44510	3.40	1.07	3.19	AT1TE55795	ATCOPIA4	LTR/Copia
AT1G45120	2.74	9.59	0.29	AT1TE56445	ATCOPIA1	LTR/Copia
AT1G46120	1.28	0.09	14.15	AT1TE57025	ATCOPIA63	LTR/Copia
AT1G47520	2.88	1.25	2.31	AT1TE57805	ATCOPIA15	LTR/Copia
AT1G47650	1.11	0.18	6.17	AT1TE58075	ATCOPIA38A	LTR/Copia
AT1G50810	1.89	9.47	0.20	AT1TE62440	ATCOPIA36	LTR/Copia
AT1G52210	1.54	0.33	4.66	AT1TE64390	ATCOPIA94	LTR/Copia
AT1G57640	3.16	0.62	5.09	AT1TE70490	ATCOPIA22	LTR/Copia
AT1G58561	1.79	0.21	8.63	AT1TE71775	ATCOPIA8B	LTR/Copia
AT1G58889	2.33	9.36	0.25	AT1TE71950	ATRE1	LTR/Copia
AT1G59265	1.07	8.14	0.13	AT1TE72060	ATRE1	LTR/Copia
AT1G60020	3.45	0.68	5.09	AT1TE72970	ATCOPIA5	LTR/Copia
AT1G62460	3.01	7.19	0.42	AT1TE76215	ATCOPIA51	LTR/Copia
AT1G65347	2.69	0.47	5.78	AT1TE79810	ATCOPIA15	LTR/Copia
AT1G70010	2.57	0.56	4.58	AT1TE86360	ATCOPIA11	LTR/Copia
AT2G03100	2.15	0.36	5.95	AT2TE04130	ATCOPIA56	LTR/Copia
AT2G04490	0.87	1.78	0.49	AT2TE07175	ATCOPIA61	LTR/Copia
AT2G05240	2.16	1.05	2.07	AT2TE08785	ATCOPIA66	LTR/Copia
AT2G05930	1.60	0.61	2.60	AT2TE10495	ATCOPIA58	LTR/Copia
AT2G06830	2.69	0.50	5.41	AT2TE12115	ATCOPIA67	LTR/Copia
AT2G06840	4.50	1.49	3.03	AT2TE12140	ATCOPIA75	LTR/Copia
AT2G06930	1.43	0.12	12.29	AT2TE12470	ATCOPIA68	LTR/Copia
AT2G06950	48.32	148.83	0.32	AT2TE12510	ATCOPIA8A	LTR/Copia
AT2G07010	1.79	0.65	2.75	AT2TE12660	ATCOPIA13	LTR/Copia
AT2G07080	65.01	14.77	4.40	AT2TE12815	ATCOPIA43	LTR/Copia
AT2G07420	1.98	0.06	34.06	AT2TE13385	ATCOPIA20	LTR/Copia
AT2G07683	13.39	6.07	2.20	AT2TE14285	ATCOPIA8A	LTR/Copia
AT2G07685	2.47	0.12	21.20	AT2TE14315	ATCOPIA55	LTR/Copia
AT2G07693	5.45	1.38	3.94	AT2TE14490	ATCOPIA76	LTR/Copia
AT2G07694	1.59	0.24	6.64	AT2TE14495	ATCOPIA23	LTR/Copia
AT2G07697	5.20	0.47	11.01	AT2TE14540	ATCOPIA22	LTR/Copia
AT2G07703	1.19	0.33	3.62	AT2TE14650	ATCOPIA53	LTR/Copia
AT2G07704	1.43	0.23	6.14	AT2TE14660	ATCOPIA11	LTR/Copia
AT2G07729	3.20	0.45	7.17	AT2TE15045	ATCOPIA11	LTR/Copia
AT2G07735	1.36	0.00	--	AT2TE15150	ATCOPIA49	LTR/Copia

AT2G07736	2.06	0.23	8.83	AT2TE15150	ATCOPIA49	LTR/Copia
AT2G07769	1.60	0.12	13.70	AT2TE14160	ATCOPIA50	LTR/Copia
AT2G10860	1.12	0.00	--	AT2TE17985	ATCOPIA41	LTR/Copia
AT2G11220	1.00	0.32	3.11	AT2TE18710	ATCOPIA35	LTR/Copia
AT2G13110	3.04	0.00	--	AT2TE21855	ATCOPIA27	LTR/Copia
AT2G13700	1.76	0.00	--	AT2TE23120	ATCOPIA97	LTR/Copia
AT2G13940	3.13	0.92	3.41	AT2TE23855	ATCOPIA13	LTR/Copia
AT2G14200	1.07	0.00	--	AT2TE24460	ATCOPIA45	LTR/Copia
AT2G14220	1.38	3.93	0.35	AT2TE24520	ATCOPIA77	LTR/Copia
AT2G15650	2.92	10.10	0.29	AT2TE27820	ATCOPIA36	LTR/Copia
AT2G15920	1.56	0.29	5.37	AT2TE28260	ATCOPIA38	LTR/Copia
AT2G16000	1.37	0.39	3.54	AT2TE28325	ATCOPIA38B	LTR/Copia
AT2G16670	1.29	0.17	7.41	AT2TE29450	ATCOPIA70	LTR/Copia
AT2G24660	1.40	0.53	2.64	AT2TE45020	ATCOPIA12	LTR/Copia
AT2G29165	1.06	0.09	11.69	AT2TE54780	ATCOPIA63	LTR/Copia
AT3G28160	5.33	14.55	0.37	AT3TE43605	ATCOPIA50	LTR/Copia
AT3G28315	3.78	1.24	3.04	AT3TE44000	ATCOPIA31A	LTR/Copia
AT3G29032	3.26	1.01	3.21	AT3TE45800	ATCOPIA5	LTR/Copia
AT3G29510	3.68	0.17	21.03	AT3TE47235	ATCOPIA27	LTR/Copia
AT3G30582	1.11	0.17	6.38	AT3TE50550	ATCOPIA67	LTR/Copia
AT3G42837	1.78	0.15	11.95	AT3TE60925	ATCOPIA13	LTR/Copia
AT3G43635	1.41	0.17	8.05	AT3TE62940	ATCOPIA43	LTR/Copia
AT3G43688	1.17	0.06	20.17	AT3TE63050	ATCOPIA43	LTR/Copia
AT3G43825	3.52	1.25	2.83	AT3TE63380	ATCOPIA12	LTR/Copia
AT3G43955	4.83	15.13	0.32	AT3TE63765	ATCOPIA62	LTR/Copia
AT3G44215	1.16	0.12	9.93	AT3TE64435	ATCOPIA11	LTR/Copia
AT3G44325	3.03	1.13	2.68	AT3TE64755	ATCOPIA96	LTR/Copia
AT3G45095	2.35	0.83	2.82	AT3TE66880	ATCOPIA46	LTR/Copia
AT3G45520	1.65	0.21	7.97	AT3TE67635	ATCOPIA32	LTR/Copia
AT3G50625	1.96	0.27	7.38	AT3TE76225	ATCOPIA52	LTR/Copia
AT3G60565	13.63	6.60	2.06	AT3TE91175	ATCOPIA46	LTR/Copia
AT3G62455	2.85	7.65	0.37	AT3TE94195	ATCOPIA23	LTR/Copia
AT4G04410	0.50	3.66	0.14	AT4TE10320	ATCOPIA93	LTR/Copia
AT4G04420	2.21	1.08	2.04	AT4TE10335	ATCOPIA58	LTR/Copia
AT4G05073	1.04	0.24	4.34	AT4TE12175	ATCOPIA69	LTR/Copia
AT4G05133	2.10	0.50	4.17	AT4TE12355	ATCOPIA56	LTR/Copia
AT4G08054	1.03	0.30	3.46	AT4TE20510	ROMANIAT5	LTR/Copia
AT4G09313	4.45	0.94	4.71	AT4TE24880	ATCOPIA9	LTR/Copia
AT4G09316	1.11	0.21	5.39	AT4TE24910	ATCOPIA9	LTR/Copia
AT4G09455	3.55	7.67	0.46	AT4TE25200	ATCOPIA51	LTR/Copia
AT4G10460	1.15	0.00	--	AT4TE27640	ATCOPIA50	LTR/Copia
AT4G11375	2.62	0.32	8.09	AT4TE29830	ATCOPIA22	LTR/Copia
AT4G16870	3.11	33.17	0.09	AT4TE42860	ATCOPIA4	LTR/Copia
AT4G20365	1.48	0.59	2.50	AT4TE50435	ATCOPIA47	LTR/Copia
AT4G22040	3.29	0.48	6.89	AT4TE53965	ATCOPIA22	LTR/Copia
AT4G27200	2.25	0.83	2.72	AT4TE64175	ATCOPIA8A	LTR/Copia
AT4G27210	3.28	1.48	2.22	AT4TE64180	ATCOPIA8B	LTR/Copia
AT5G01185	4.42	0.23	18.98	AT5TE00230	ATCOPIA27	LTR/Copia
AT5G17125	4.54	1.52	2.99	AT5TE20395	ATCOPIA93	LTR/Copia
AT5G25045	3.11	0.56	5.54	AT5TE31200	ATCOPIA22	LTR/Copia
AT5G25615	4.91	0.17	28.11	AT5TE32355	ATCOPIA27	LTR/Copia
AT5G26618	1.10	0.09	12.16	AT5TE33540	ATCOPIA63	LTR/Copia

AT5G28468	1.00	0.15	6.76	AT5TE37985	ATCOPIA70	LTR/Copia
AT5G28635	1.18	0.24	4.96	AT5TE38745	ATCOPIA13	LTR/Copia
AT5G28776	5.42	1.36	3.99	AT5TE39295	ATCOPIA45	LTR/Copia
AT5G29646	1.18	0.21	5.72	AT5TE41975	ATCOPIA66	LTR/Copia
AT5G30269	4.20	0.91	4.61	AT5TE41885	ATCOPIA65	LTR/Copia
AT5G30584	1.33	0.32	4.11	AT5TE41245	ROMANIAT5	LTR/Copia
AT5G32484	2.99	0.23	12.84	AT5TE43215	ATCOPIA27	LTR/Copia
AT5G32702	1.26	0.00	--	AT5TE43780	ATCOPIA51	LTR/Copia
AT5G35820	2.53	0.42	6.03	AT5TE49915	ATCOPIA56	LTR/Copia
AT5G37145	1.03	0.06	17.66	AT5TE52925	ATCOPIA65	LTR/Copia
AT5G38035	3.45	0.56	6.13	AT5TE54905	ATCOPIA87	LTR/Copia
AT5G38437	1.00	0.12	8.62	AT5TE55665	ATCOPIA22	LTR/Copia
AT5G39095	1.81	0.21	8.75	AT5TE56585	ATCOPIA25	LTR/Copia
AT5G39155	11.39	1.80	6.34	AT5TE56645	ATCOPIA83	LTR/Copia
AT5G39185	11.34	1.80	6.31	AT5TE56665	ATCOPIA83	LTR/Copia
AT5G43800	6.42	1.27	5.06	AT5TE63610	ENDOVIR1	LTR/Copia
AT5G45605	2.80	0.59	4.75	AT5TE66565	ATCOPIA31	LTR/Copia
AT5G46665	2.16	0.91	2.39	AT5TE68140	ATCOPIA58	LTR/Copia
AT5G47445	6.27	14.30	0.44	AT5TE69240	ATCOPIA62	LTR/Copia
AT5G53815	3.37	1.21	2.79	AT5TE78710	ATCOPIA16	LTR/Copia
AT5G54203	3.43	0.65	5.26	AT5TE79225	ATCOPIA22	LTR/Copia
AT5G55875	2.29	0.77	2.98	AT5TE81385	ATCOPIA13	LTR/Copia
AT5G57126	1.05	0.06	17.97	AT5TE83215	ATCOPIA49	LTR/Copia
AT1G31993	7.72	0.23	33.16	AT1TE37210	ATLANTYS3	LTR/Gypsy
AT1G32040	1.66	0.38	4.36	AT1TE37265	ATLANTYS2	LTR/Gypsy
AT1G34600	1.15	0.00	--	AT1TE41345	ATLANTYS2	LTR/Gypsy
AT1G35790	4.45	0.65	6.88	AT1TE43420	ATHILA4	LTR/Gypsy
AT1G35970	1.30	0.39	3.36	AT1TE43805	ATHILA4	LTR/Gypsy
AT1G36120	7.07	0.74	9.52	AT1TE44230	ATGP1	LTR/Gypsy
AT1G36130	3.23	1.13	2.86	AT1TE44235	ATHILA4A	LTR/Gypsy
AT1G36210	2.39	0.59	4.03	AT1TE44525	ATHILA4	LTR/Gypsy
AT1G36300	2.74	0.27	10.33	AT1TE44670	ATHILA6B	LTR/Gypsy
AT1G36305	10.41	0.62	16.77	AT1TE44720	ATGP1	LTR/Gypsy
AT1G36520	8.57	2.20	3.90	AT1TE45135	ATHILA2	LTR/Gypsy
AT1G36530	1.07	0.12	9.20	AT1TE45155	ATHILA4A	LTR/Gypsy
AT1G36610	2.34	0.12	20.12	AT1TE45355	ATGP1	LTR/Gypsy
AT1G36620	1.30	0.00	--	AT1TE45355	ATGP1	LTR/Gypsy
AT1G36720	4.07	0.36	11.46	AT1TE45560	ATGP1	LTR/Gypsy
AT1G36790	1.03	0.00	--	AT1TE45770	ATHILA4	LTR/Gypsy
AT1G37015	1.19	0.56	2.12	AT1TE46140	ATHILA	LTR/Gypsy
AT1G37050	1.41	0.53	2.65	AT1TE46255	ATHILA4A	LTR/Gypsy
AT1G37060	2.11	0.50	4.19	AT1TE46265	ATHILA6B	LTR/Gypsy
AT1G37340	1.21	0.12	10.38	AT1TE46665	ATHILA3	LTR/Gypsy
AT1G37471	2.54	0.72	3.54	AT1TE46700	ATHILA	LTR/Gypsy
AT1G37735	1.32	0.36	3.72	AT1TE46770	ATHILA2	LTR/Gypsy
AT1G37867	1.29	0.00	--	AT1TE46805	ATHILA3	LTR/Gypsy
AT1G38230	2.21	0.56	3.98	AT1TE47225	ATHILA4A	LTR/Gypsy
AT1G38300	1.20	0.38	3.14	AT1TE47445	ATHILA4A	LTR/Gypsy
AT1G38360	2.07	0.30	6.95	AT1TE47540	ATHILA4C	LTR/Gypsy
AT1G38423	2.07	0.44	4.70	AT1TE47645	ATHILA4A	LTR/Gypsy
AT1G39910	1.31	0.59	2.21	AT1TE48745	ATHILA	LTR/Gypsy
AT1G40073	1.31	0.59	2.21	AT1TE48765	ATHILA	LTR/Gypsy

AT1G40093	2.70	0.48	5.65	AT1TE49260	ATHILA6B	LTR/Gypsy
AT1G40095	7.97	1.04	7.66	AT1TE49260	ATHILA6B	LTR/Gypsy
AT1G40101	3.37	0.21	16.29	AT1TE49300	ATHILA2	LTR/Gypsy
AT1G41775	3.02	0.45	6.79	AT1TE51155	ATHILA2	LTR/Gypsy
AT1G41790	2.35	0.00	--	AT1TE51175	ATLANTYS2	LTR/Gypsy
AT1G41795	3.55	0.21	17.16	AT1TE51200	ATHILA	LTR/Gypsy
AT1G41797	3.35	0.21	16.22	AT1TE51225	ATHILA	LTR/Gypsy
AT1G41803	1.48	0.00	--	AT1TE51260	ATLANTYS2	LTR/Gypsy
AT1G41840	1.07	0.50	2.13	AT1TE51370	ATHILA2	LTR/Gypsy
AT1G42045	1.77	0.65	2.71	AT1TE51695	ATHILA4A	LTR/Gypsy
AT1G42050	1.38	0.21	6.68	AT1TE51735	ATHILA2	LTR/Gypsy
AT1G42060	1.02	0.00	--	AT1TE51735	ATHILA2	LTR/Gypsy
AT1G42140	5.29	1.62	3.26	AT1TE51825	ATHILA4A	LTR/Gypsy
AT1G42320	2.94	0.17	16.82	AT1TE52025	ATGP1	LTR/Gypsy
AT1G42377	1.30	0.62	2.10	AT1TE52160	ATHILA4A	LTR/Gypsy
AT1G42605	5.23	0.23	22.47	AT1TE52795	ATGP1	LTR/Gypsy
AT1G42695	1.34	0.48	2.80	AT1TE52865	ATHILA4	LTR/Gypsy
AT1G42888	2.84	0.50	5.71	AT1TE53045	ATHILA4	LTR/Gypsy
AT1G43060	5.50	2.73	2.02	AT1TE53370	ATHILA5	LTR/Gypsy
AT1G43444	2.62	0.21	12.69	AT1TE53995	ATGP1	LTR/Gypsy
AT1G43740	1.24	0.15	8.36	AT1TE54480	ATHILA5	LTR/Gypsy
AT1G43830	1.59	0.12	13.68	AT1TE54655	ATHILA5	LTR/Gypsy
AT1G43883	2.38	0.45	5.35	AT1TE54805	ATLANTYS1	LTR/Gypsy
AT1G47565	0.88	4.40	0.20	AT1TE57910	ATGP3	LTR/Gypsy
AT1G51175	9.55	0.94	10.12	AT1TE62820	ATLANTYS1	LTR/Gypsy
AT2G01022	3.84	1.12	3.43	AT2TE00010	ATGP1	LTR/Gypsy
AT2G01026	1.54	0.06	26.36	AT2TE00015	ATGP2	LTR/Gypsy
AT2G01029	3.41	0.63	5.44	AT2TE00015	ATGP2	LTR/Gypsy
AT2G01034	1.38	0.00	--	AT2TE00075	ATGP2	LTR/Gypsy
AT2G01037	4.42	0.00	--	AT2TE00110	ATGP2	LTR/Gypsy
AT2G04670	5.66	0.24	23.68	AT2TE07550	ATGP1	LTR/Gypsy
AT2G06150	1.32	0.15	8.87	AT2TE10980	ATHILA	LTR/Gypsy
AT2G06340	1.96	0.65	3.04	AT2TE11355	ATHILA	LTR/Gypsy
AT2G06350	2.81	0.38	7.37	AT2TE11370	ATHILA6A	LTR/Gypsy
AT2G06470	2.29	0.00	--	AT2TE11540	ATGP2	LTR/Gypsy
AT2G06890	2.95	0.36	8.30	AT2TE12315	ATGP5	LTR/Gypsy
AT2G06965	1.31	0.21	6.34	AT2TE12555	ATHILA3	LTR/Gypsy
AT2G06967	1.71	0.21	8.25	AT2TE12555	ATHILA3	LTR/Gypsy
AT2G07682	26.29	6.43	4.09	AT2TE14250	ATGP3	LTR/Gypsy
AT2G07686	3.98	0.66	6.05	AT2TE14330	ATHILA7	LTR/Gypsy
AT2G07789	4.00	1.07	3.75	AT2TE15490	ATHILA	LTR/Gypsy
AT2G09589	2.82	0.52	5.39	AT2TE15920	ATHILA4A	LTR/Gypsy
AT2G09920	2.98	0.78	3.85	AT2TE16235	ATLANTYS3	LTR/Gypsy
AT2G10080	1.36	0.30	4.59	AT2TE16440	ATHILA4A	LTR/Gypsy
AT2G10110	1.22	0.48	2.55	AT2TE16475	ATHILA2	LTR/Gypsy
AT2G10120	1.33	0.41	3.22	AT2TE16485	ATHILA4A	LTR/Gypsy
AT2G10180	1.27	0.12	10.90	AT2TE16575	ATHILA2	LTR/Gypsy
AT2G10280	19.77	9.21	2.15	AT2TE16810	ATHILA4B_LTR	LTR/Gypsy
AT2G10290	1.20	0.27	4.53	AT2TE16825	ATHILA	LTR/Gypsy
AT2G10300	1.25	0.32	3.87	AT2TE16800	ATLANTYS3	LTR/Gypsy
AT2G10310	2.35	0.29	8.06	AT2TE16865	ATHILA2	LTR/Gypsy
AT2G10320	1.65	0.17	9.47	AT2TE16865	ATHILA2	LTR/Gypsy

AT2G10330	6.15	1.27	4.85	AT2TE16895	ATLANTYS3	LTR/Gypsy
AT2G10540	4.05	0.94	4.29	AT2TE17305	ATGP3	LTR/Gypsy
AT2G10600	13.04	2.01	6.49	AT2TE17420	ATHILA	LTR/Gypsy
AT2G10620	1.65	0.00	--	AT2TE17580	ATGP10	LTR/Gypsy
AT2G10660	1.55	0.24	6.49	AT2TE17625	ATHILA6A	LTR/Gypsy
AT2G10670	2.94	0.78	3.80	AT2TE17675	ATHILA	LTR/Gypsy
AT2G10780	1.92	0.06	32.96	AT2TE17875	ATGP2	LTR/Gypsy
AT2G10890	1.20	0.27	4.53	AT2TE18010	ATHILA6B	LTR/Gypsy
AT2G11230	2.65	0.17	15.16	AT2TE18715	ATHILA4	LTR/Gypsy
AT2G11430	7.02	0.12	60.27	AT2TE18980	ATGP1	LTR/Gypsy
AT2G11450	1.70	0.23	7.28	AT2TE19005	ATHILA0_I	LTR/Gypsy
AT2G11700	2.58	0.56	4.58	AT2TE19515	ATHILA	LTR/Gypsy
AT2G11720	1.17	0.06	20.02	AT2TE19535	ATHILA	LTR/Gypsy
AT2G11770	1.91	0.24	8.01	AT2TE19590	ATHILA4C	LTR/Gypsy
AT2G12020	3.41	0.48	7.14	AT2TE20075	ATHILA4C	LTR/Gypsy
AT2G12570	1.11	0.15	7.50	AT2TE21030	ATHILA6B	LTR/Gypsy
AT2G12610	1.89	0.80	2.35	AT2TE21065	ATHILA4A	LTR/Gypsy
AT2G12740	23.66	1.98	11.92	AT2TE21345	ATGP1	LTR/Gypsy
AT2G12750	18.30	0.97	18.86	AT2TE21355	ATGP1	LTR/Gypsy
AT2G12760	8.79	0.54	16.40	AT2TE21360	ATGP1	LTR/Gypsy
AT2G12800	1.13	0.00	--	AT2TE21420	ATHILA2	LTR/Gypsy
AT2G12930	1.87	0.32	5.78	AT2TE21605	ATLANTYS2	LTR/Gypsy
AT2G12970	3.40	0.33	10.32	AT2TE21705	ATGP1	LTR/Gypsy
AT2G13020	2.24	0.06	38.41	AT2TE21745	ATHILA4C	LTR/Gypsy
AT2G13050	1.27	0.00	--	AT2TE21765	ATLANTYS1	LTR/Gypsy
AT2G13080	1.03	0.00	--	AT2TE21760	ATLANTYS1	LTR/Gypsy
AT2G13170	1.67	0.77	2.17	AT2TE22065	ATGP5	LTR/Gypsy
AT2G13260	1.17	0.15	7.91	AT2TE22235	ATHILA7A	LTR/Gypsy
AT2G13270	2.16	0.89	2.44	AT2TE22245	ATHILA4A	LTR/Gypsy
AT2G13330	1.87	0.54	3.48	AT2TE22345	ATLANTYS1	LTR/Gypsy
AT2G13380	2.37	0.71	3.33	AT2TE22490	TAT1_ATH	LTR/Gypsy
AT2G13390	2.46	0.76	3.22	AT2TE22505	ATHILA2	LTR/Gypsy
AT2G13740	2.07	0.77	2.69	AT2TE23180	ATHILA4A	LTR/Gypsy
AT2G13830	3.82	0.50	7.59	AT2TE23510	ATGP1	LTR/Gypsy
AT2G13860	3.94	0.27	14.88	AT2TE23670	ATGP1	LTR/Gypsy
AT2G13990	3.60	0.57	6.34	AT2TE23940	ATGP2	LTR/Gypsy
AT2G14040	1.43	0.21	6.89	AT2TE24035	ATHILA7	LTR/Gypsy
AT2G14400	1.31	0.00	--	AT2TE24865	TAT1_ATH	LTR/Gypsy
AT2G14640	3.45	0.62	5.57	AT2TE25515	ATGP3	LTR/Gypsy
AT2G14650	5.09	0.50	10.10	AT2TE25540	ATGP1	LTR/Gypsy
AT2G15100	1.43	0.21	6.92	AT2TE26780	TAT1_ATH	LTR/Gypsy
AT2G15410	8.16	1.27	6.44	AT2TE27460	ATLANTYS1	LTR/Gypsy
AT3G17050	20.73	134.92	0.15	AT3TE24450	ATGP1	LTR/Gypsy
AT3G29076	3.29	0.30	11.07	AT3TE45980	ATGP1	LTR/Gypsy
AT3G29480	3.93	0.21	18.99	AT3TE47230	ATGP1	LTR/Gypsy
AT3G29615	2.33	0.18	12.89	AT3TE47590	ATLANTYS2	LTR/Gypsy
AT3G29641	2.38	0.80	2.98	AT3TE47875	ATHILA4A	LTR/Gypsy
AT3G30400	1.78	0.15	12.00	AT3TE50035	ATHILA7A	LTR/Gypsy
AT3G30433	1.30	0.48	2.73	AT3TE50255	ATHILA4A	LTR/Gypsy
AT3G30436	1.31	0.30	4.42	AT3TE50260	ATHILA4	LTR/Gypsy
AT3G30655	1.72	0.38	4.50	AT3TE50630	ATHILA	LTR/Gypsy
AT3G30695	24.74	5.48	4.51	AT3TE50760	ATHILA2	LTR/Gypsy

AT3G30703	10.05	0.63	16.04	AT3TE50775	ATGP1	LTR/Gypsy
AT3G30749	1.90	0.57	3.34	AT3TE51300	ATHILA2	LTR/Gypsy
AT3G30802	6.77	0.45	15.19	AT3TE51505	ATGP1	LTR/Gypsy
AT3G30803	1.12	0.00	--	AT3TE51505	ATGP1	LTR/Gypsy
AT3G30810	2.85	0.27	10.74	AT3TE51535	ATLANTYS1	LTR/Gypsy
AT3G30811	1.62	0.00	--	AT3TE51535	ATLANTYS1	LTR/Gypsy
AT3G30819	1.56	0.44	3.55	AT3TE51590	ATHILA3	LTR/Gypsy
AT3G30821	1.30	0.00	--	AT3TE51610	ATHILA7A	LTR/Gypsy
AT3G30825	1.55	0.27	5.86	AT3TE51650	ATHILA2	LTR/Gypsy
AT3G30833	1.12	0.42	2.68	AT3TE51685	ATGP6	LTR/Gypsy
AT3G30838	1.41	0.15	9.47	AT3TE51810	ATHILA2	LTR/Gypsy
AT3G30846	5.07	0.00	--	AT3TE51930	ATGP1	LTR/Gypsy
AT3G31310	1.17	0.00	--	AT3TE52155	ATLANTYS2	LTR/Gypsy
AT3G31490	1.10	0.00	--	AT3TE52725	ATGP1	LTR/Gypsy
AT3G31500	1.36	0.00	--	AT3TE52730	ATHILA7A	LTR/Gypsy
AT3G31935	2.06	0.36	5.79	AT3TE53075	ATHILA0_I	LTR/Gypsy
AT3G31945	1.62	0.29	5.57	AT3TE53080	ATLANTYS2	LTR/Gypsy
AT3G31970	5.00	0.78	6.45	AT3TE53170	ATGP1	LTR/Gypsy
AT3G32033	1.03	0.21	4.97	AT3TE53290	ATHILA4A	LTR/Gypsy
AT3G32092	2.95	0.92	3.21	AT3TE53605	ATLANTYS2	LTR/Gypsy
AT3G32118	1.08	0.21	5.22	AT3TE53690	ATHILA	LTR/Gypsy
AT3G32164	1.73	0.15	11.65	AT3TE53765	ATLANTYS1	LTR/Gypsy
AT3G32168	1.58	0.56	2.81	AT3TE53835	ATLANTYS1	LTR/Gypsy
AT3G32195	1.03	0.21	4.97	AT3TE53845	ATGP5	LTR/Gypsy
AT3G32210	8.92	4.13	2.16	AT3TE53970	ATHILA	LTR/Gypsy
AT3G32383	1.38	0.09	15.27	AT3TE54450	ATLANTYS3	LTR/Gypsy
AT3G32894	2.06	0.24	8.61	AT3TE54710	ATHILA0_I	LTR/Gypsy
AT3G32895	7.44	2.38	3.13	AT3TE54760	ATGP5	LTR/Gypsy
AT3G32917	3.36	0.09	37.19	AT3TE54860	ATGP2N	LTR/Gypsy
AT3G32970	2.55	0.53	4.82	AT3TE55025	ATHILA2	LTR/Gypsy
AT3G32975	1.34	0.21	6.46	AT3TE55035	ATHILA6B	LTR/Gypsy
AT3G33058	3.18	0.89	3.57	AT3TE55330	ATHILA2	LTR/Gypsy
AT3G33069	2.52	1.15	2.19	AT3TE56630	ATHILA	LTR/Gypsy
AT3G33076	8.14	2.84	2.87	AT3TE56040	ATGP3	LTR/Gypsy
AT3G33084	8.40	3.10	2.71	AT3TE55470	ATGP7	LTR/Gypsy
AT3G33091	4.40	1.42	3.11	AT3TE56700	ATHILA	LTR/Gypsy
AT3G33100	3.27	0.94	3.47	AT3TE56735	ATHILA	LTR/Gypsy
AT3G33106	2.37	0.51	4.64	AT3TE57050	ATHILA	LTR/Gypsy
AT3G33109	1.16	0.56	2.06	AT3TE57050	ATHILA	LTR/Gypsy
AT3G33115	1.10	0.06	18.86	AT3TE57085	ATLANTYS2	LTR/Gypsy
AT3G33133	2.26	0.92	2.46	AT3TE57180	ATHILA	LTR/Gypsy
AT3G33136	1.65	0.53	3.12	AT3TE57210	ATHILA3	LTR/Gypsy
AT3G33175	1.18	0.15	7.97	AT3TE57465	ATHILA4A	LTR/Gypsy
AT3G33537	4.45	1.12	3.96	AT3TE57685	ATHILA	LTR/Gypsy
AT3G33555	3.09	1.33	2.34	AT3TE57670	ATHILA4A	LTR/Gypsy
AT3G33595	1.43	0.06	24.58	AT3TE57590	ATHILA3	LTR/Gypsy
AT3G39230	2.23	0.00	--	AT3TE58050	ATGP2	LTR/Gypsy
AT3G39935	1.03	0.15	6.92	AT3TE58065	ATHILA4	LTR/Gypsy
AT3G42052	12.52	31.09	0.40	AT3TE58550	TAT1_ATH	LTR/Gypsy
AT3G42057	6.66	0.12	57.17	AT3TE58575	ATLANTYS3	LTR/Gypsy
AT3G42070	1.17	0.06	20.02	AT3TE58605	ATHILA4C	LTR/Gypsy
AT3G42120	1.26	0.30	4.24	AT3TE58720	ATHILA4A	LTR/Gypsy

AT3G42252	3.25	1.25	2.59	AT3TE59100	ATHILA2	LTR/Gypsy
AT3G42256	1.35	0.30	4.53	AT3TE59155	ATHILA2	LTR/Gypsy
AT3G42257	1.54	0.00	--	AT3TE59185	ATHILA6B	LTR/Gypsy
AT3G42270	1.54	0.15	10.39	AT3TE59205	ATHILA4	LTR/Gypsy
AT3G42290	2.12	0.06	36.37	AT3TE59230	ATGP1	LTR/Gypsy
AT3G42300	1.50	0.09	16.62	AT3TE59245	ATHILA4C	LTR/Gypsy
AT3G42313	10.28	0.92	11.20	AT3TE59290	ATGP1	LTR/Gypsy
AT3G42431	1.10	0.41	2.67	AT3TE59600	ATHILA2	LTR/Gypsy
AT3G42436	1.38	0.24	5.79	AT3TE59660	ATHILA4C	LTR/Gypsy
AT3G42445	1.13	0.18	6.27	AT3TE59695	ATHILA2	LTR/Gypsy
AT3G42626	1.58	0.12	13.55	AT3TE60200	ATHILA3	LTR/Gypsy
AT3G42645	3.87	1.69	2.28	AT3TE60240	ATGP5	LTR/Gypsy
AT3G42836	1.22	0.21	5.89	AT3TE60910	ATLANTYS3	LTR/Gypsy
AT3G42935	1.13	0.21	5.47	AT3TE61145	ATGP3	LTR/Gypsy
AT3G42993	1.20	0.00	--	AT3TE61290	ATLANTYS2	LTR/Gypsy
AT3G42996	1.81	0.00	--	AT3TE61295	ATGP2	LTR/Gypsy
AT3G43090	1.13	0.50	2.27	AT3TE61455	ATGP8	LTR/Gypsy
AT3G43144	1.83	0.36	5.14	AT3TE61535	ATHILA0_I	LTR/Gypsy
AT3G43151	1.67	0.32	5.17	AT3TE61630	ATLANTYS2	LTR/Gypsy
AT3G43154	2.88	0.68	4.21	AT3TE61640	ATHILA2	LTR/Gypsy
AT3G43156	1.90	0.71	2.68	AT3TE61645	ATHILA	LTR/Gypsy
AT3G43157	1.16	0.15	7.79	AT3TE61645	ATHILA	LTR/Gypsy
AT3G43302	2.14	0.21	10.36	AT3TE62000	ATLANTYS3	LTR/Gypsy
AT3G43304	2.01	0.41	4.85	AT3TE62020	ATHILA0_I	LTR/Gypsy
AT3G43307	5.08	1.89	2.69	AT3TE62025	ATHILA2	LTR/Gypsy
AT3G43566	1.87	0.27	7.06	AT3TE62685	ATHILA0_I	LTR/Gypsy
AT3G43654	4.37	1.12	3.89	AT3TE62965	ATGP3	LTR/Gypsy
AT3G43675	1.00	0.12	8.60	AT3TE63020	ATGP3	LTR/Gypsy
AT3G43680	3.81	0.80	4.75	AT3TE63035	ATLANTYS2	LTR/Gypsy
AT3G43681	4.39	0.32	13.57	AT3TE63035	ATLANTYS2	LTR/Gypsy
AT3G43830	2.11	0.84	2.51	AT3TE63410	ATGP8	LTR/Gypsy
AT3G43862	2.53	0.38	6.62	AT3TE63540	ATHILA2	LTR/Gypsy
AT3G43863	6.65	2.86	2.33	AT3TE63540	ATHILA2	LTR/Gypsy
AT3G43864	1.00	0.06	17.24	AT3TE63540	ATHILA2	LTR/Gypsy
AT3G44093	1.84	0.15	12.41	AT3TE64120	ATGP3	LTR/Gypsy
AT3G44796	13.58	0.50	26.95	AT3TE66175	ATGP1	LTR/Gypsy
AT3G54823	3.00	1.07	2.81	AT3TE82555	ATGP3	LTR/Gypsy
AT3G60935	1.07	0.06	18.39	AT3TE91745	ATLANTYS2	LTR/Gypsy
AT3G62475	3.60	0.92	3.92	AT3TE94210	ATLANTYS3	LTR/Gypsy
AT3G62490	4.14	9.24	0.45	AT3TE94220	ATLANTYS3	LTR/Gypsy
AT4G03650	2.33	0.30	7.85	AT4TE08245	ATGP1	LTR/Gypsy
AT4G03770	3.30	0.23	14.16	AT4TE08445	ATLANTYS2	LTR/Gypsy
AT4G03790	1.86	0.41	4.50	AT4TE08475	ATHILA2	LTR/Gypsy
AT4G03816	3.15	0.50	6.25	AT4TE08850	ATHILA2	LTR/Gypsy
AT4G03840	1.36	0.00	--	AT4TE08980	ATGP2	LTR/Gypsy
AT4G03860	1.25	0.27	4.72	AT4TE08990	ATHILA6A	LTR/Gypsy
AT4G04050	1.57	0.27	5.92	AT4TE09385	ATLANTYS1	LTR/Gypsy
AT4G04070	1.00	0.09	11.11	AT4TE09385	ATLANTYS1	LTR/Gypsy
AT4G04157	1.44	0.00	--	AT4TE09560	ATLANTYS2	LTR/Gypsy
AT4G04165	2.04	0.45	4.57	AT4TE09560	ATLANTYS2	LTR/Gypsy
AT4G04310	1.08	0.15	7.27	AT4TE09925	ATGP5	LTR/Gypsy
AT4G05570	1.33	0.27	5.02	AT4TE13320	ATHILA2	LTR/Gypsy

AT4G05613	1.00	0.06	17.24	AT4TE13835	ATHILA	LTR/Gypsy
AT4G06485	3.41	0.98	3.47	AT4TE14295	ATHILA2	LTR/Gypsy
AT4G06499	1.15	0.15	7.74	AT4TE14500	ATHILA3	LTR/Gypsy
AT4G06506	1.79	0.45	4.02	AT4TE14540	ATHILA2	LTR/Gypsy
AT4G06509	3.41	0.53	6.44	AT4TE14555	ATGP10	LTR/Gypsy
AT4G06510	1.87	0.06	32.07	AT4TE14580	ATGP10	LTR/Gypsy
AT4G06516	1.40	0.06	24.00	AT4TE15030	ATHILA6A	LTR/Gypsy
AT4G06517	2.23	0.06	38.30	AT4TE15030	ATHILA6A	LTR/Gypsy
AT4G06518	1.39	0.00	--	AT4TE15005	ATHILA3	LTR/Gypsy
AT4G06529	1.18	0.48	2.48	AT4TE15230	TAT1_ATH	LTR/Gypsy
AT4G06556	1.15	0.36	3.23	AT4TE15675	ATHILA4C	LTR/Gypsy
AT4G06584	1.36	0.18	7.50	AT4TE16020	ATLANTYS1	LTR/Gypsy
AT4G06656	25.52	8.78	2.90	AT4TE16900	ATHILA	LTR/Gypsy
AT4G06660	4.57	1.39	3.29	AT4TE16940	ATGP10	LTR/Gypsy
AT4G06664	14.55	5.39	2.70	AT4TE16945	ATHILA	LTR/Gypsy
AT4G06666	4.70	2.04	2.31	AT4TE16945	ATHILA	LTR/Gypsy
AT4G06670	1.09	0.41	2.63	AT4TE16980	ATGP3	LTR/Gypsy
AT4G06704	1.95	0.27	7.35	AT4TE17240	ATHILA2	LTR/Gypsy
AT4G06712	20.75	8.02	2.59	AT4TE17360	ATHILA	LTR/Gypsy
AT4G06714	8.34	2.71	3.07	AT4TE17360	ATHILA	LTR/Gypsy
AT4G06724	1.12	0.36	3.16	AT4TE17480	ATHILA	LTR/Gypsy
AT4G06726	2.74	0.68	4.01	AT4TE17490	ATHILA2	LTR/Gypsy
AT4G06728	1.36	0.18	7.50	AT4TE17500	ATHILA	LTR/Gypsy
AT4G06734	1.35	0.00	--	AT4TE17510	ATHILA2	LTR/Gypsy
AT4G06736	1.17	0.17	6.72	AT4TE17510	ATHILA2	LTR/Gypsy
AT4G06752	1.68	0.12	14.44	AT4TE17765	ATGP1	LTR/Gypsy
AT4G07315	1.42	0.15	9.53	AT4TE17800	TAT1_ATH	LTR/Gypsy
AT4G07334	1.83	0.23	7.87	AT4TE17925	ATHILA4C	LTR/Gypsy
AT4G07339	1.20	0.56	2.13	AT4TE18010	ATHILA4A	LTR/Gypsy
AT4G07454	1.69	0.32	5.22	AT4TE18225	ATGP7	LTR/Gypsy
AT4G07456	2.61	0.86	3.04	AT4TE18245	ATHILA	LTR/Gypsy
AT4G07458	4.73	1.52	3.12	AT4TE18245	ATHILA	LTR/Gypsy
AT4G07460	1.84	0.36	5.19	AT4TE18270	ATHILA4A	LTR/Gypsy
AT4G07498	1.00	0.00	--	AT4TE18360	ATHILA4	LTR/Gypsy
AT4G07502	2.15	0.65	3.29	AT4TE18365	ATHILA4C	LTR/Gypsy
AT4G07504	1.02	0.15	6.86	AT4TE18365	ATHILA4C	LTR/Gypsy
AT4G07507	1.83	7.76	0.24	AT4TE18380	ATHILA4A	LTR/Gypsy
AT4G07528	1.12	0.18	6.22	AT4TE18500	ATLANTYS1	LTR/Gypsy
AT4G07600	1.07	0.18	5.93	AT4TE18695	ATHILA2	LTR/Gypsy
AT4G07664	1.60	0.21	7.72	AT4TE18825	ATHILA0_I	LTR/Gypsy
AT4G07668	1.51	0.45	3.38	AT4TE18845	ATGP5	LTR/Gypsy
AT4G07700	2.08	0.38	5.44	AT4TE18880	ATGP5	LTR/Gypsy
AT4G07725	1.66	0.21	8.04	AT4TE18995	ATLANTYS1	LTR/Gypsy
AT4G07733	4.18	0.68	6.11	AT4TE19030	ATHILA4C	LTR/Gypsy
AT4G07738	3.71	0.54	6.91	AT4TE19050	ATHILA4C	LTR/Gypsy
AT4G07742	1.29	0.47	2.74	AT4TE19075	ATLANTYS1	LTR/Gypsy
AT4G07850	1.25	0.41	3.03	AT4TE19655	ATGP7	LTR/Gypsy
AT4G07856	2.71	0.24	11.34	AT4TE19675	ATGP1	LTR/Gypsy
AT4G07890	1.88	0.00	--	AT4TE19755	ATHILA8A	LTR/Gypsy
AT4G07893	2.59	0.00	--	AT4TE19780	ATHILA2	LTR/Gypsy
AT4G07920	1.11	0.00	--	AT4TE19830	ATLANTYS1	LTR/Gypsy
AT4G07933	4.88	2.35	2.08	AT4TE19840	ATLANTYS2	LTR/Gypsy

AT4G07934	2.21	0.71	3.10	AT4TE19845	ATHILA4A	LTR/Gypsy
AT4G07935	1.69	0.06	28.98	AT4TE19855	ATHILA4B_LTR	LTR/Gypsy
AT4G08030	1.21	0.48	2.53	AT4TE20205	ATHILA4	LTR/Gypsy
AT4G08050	4.26	0.38	11.17	AT4TE20400	ATHILA2	LTR/Gypsy
AT4G08078	1.36	0.53	2.56	AT4TE20745	ATHILA2	LTR/Gypsy
AT4G08080	1.92	0.38	5.03	AT4TE20745	ATHILA2	LTR/Gypsy
AT4G08096	1.00	0.00	--	AT4TE20885	ATGP2N	LTR/Gypsy
AT4G08099	1.24	0.56	2.21	AT4TE20915	ATLANTYS2	LTR/Gypsy
AT4G08101	1.35	0.44	3.06	AT4TE20930	ATHILA4A	LTR/Gypsy
AT4G08103	1.54	0.29	5.27	AT4TE20950	ATHILA6A	LTR/Gypsy
AT4G08105	5.09	0.15	34.26	AT4TE20975	ATGP1	LTR/Gypsy
AT4G08114	1.29	0.15	8.71	AT4TE21090	ATLANTYS1	LTR/Gypsy
AT4G08120	1.22	0.21	5.89	AT4TE21135	ATHILA4A	LTR/Gypsy
AT4G08130	1.13	0.23	4.86	AT4TE21135	ATHILA4A	LTR/Gypsy
AT4G08131	1.20	0.30	4.04	AT4TE21170	ATHILA3	LTR/Gypsy
AT4G08138	4.90	0.39	12.65	AT4TE21295	ATGP1	LTR/Gypsy
AT4G08490	2.32	0.76	3.04	AT4TE22565	ATHILA4A	LTR/Gypsy
AT4G10580	3.72	0.74	5.00	AT4TE27915	ATGP1	LTR/Gypsy
AT4G16910	0.45	1.91	0.24	AT4TE42950	ATGP2	LTR/Gypsy
AT4G20490	2.66	0.59	4.47	AT4TE50640	ATLANTYS3	LTR/Gypsy
AT4G22415	1.03	0.12	8.83	AT4TE54700	ATGP3	LTR/Gypsy
AT4G32220	1.68	8.65	0.19	AT4TE74230	ATLANTYS3	LTR/Gypsy
AT5G12085	1.98	0.65	3.04	AT5TE14165	ATGP3	LTR/Gypsy
AT5G14810	1.54	8.82	0.17	AT5TE17325	ATGP9B	LTR/Gypsy
AT5G26236	16.35	0.41	39.52	AT5TE33235	ATGP1	LTR/Gypsy
AT5G26283	1.76	0.17	10.10	AT5TE33315	ATHILA7A	LTR/Gypsy
AT5G27885	5.10	0.59	8.67	AT5TE36040	ATGP1	LTR/Gypsy
AT5G27895	0.82	2.61	0.32	AT5TE36080	ATLANTYS3	LTR/Gypsy
AT5G27965	1.39	0.09	15.37	AT5TE36395	ATGP1	LTR/Gypsy
AT5G28335	6.82	2.48	2.75	AT5TE37660	ATGP3	LTR/Gypsy
AT5G28495	3.08	0.47	6.52	AT5TE38130	ATHILA4C	LTR/Gypsy
AT5G28593	1.86	0.32	5.75	AT5TE38635	ATHILA4	LTR/Gypsy
AT5G28596	2.08	0.62	3.34	AT5TE38650	ATHILA6A	LTR/Gypsy
AT5G28624	1.14	0.21	5.52	AT5TE38710	ATHILA7	LTR/Gypsy
AT5G28692	1.63	0.68	2.38	AT5TE39050	ATHILA4	LTR/Gypsy
AT5G28696	1.81	0.59	3.04	AT5TE39085	ATHILA3	LTR/Gypsy
AT5G28715	9.37	1.58	5.94	AT5TE39170	ATGP1	LTR/Gypsy
AT5G28865	2.05	0.06	35.21	AT5TE39540	ATLANTYS2	LTR/Gypsy
AT5G28870	1.35	0.09	14.90	AT5TE39550	ATLANTYS3	LTR/Gypsy
AT5G29020	1.66	0.70	2.36	AT5TE40100	ATLANTYS2	LTR/Gypsy
AT5G29043	1.36	0.24	5.67	AT5TE40260	ATHILA4	LTR/Gypsy
AT5G29046	1.72	0.38	4.52	AT5TE40275	ATHILA	LTR/Gypsy
AT5G29053	1.55	0.29	5.32	AT5TE40280	ATLANTYS1	LTR/Gypsy
AT5G29075	3.63	0.98	3.69	AT5TE40340	ATHILA4A	LTR/Gypsy
AT5G29720	2.16	0.27	8.15	AT5TE40940	ATHILA4	LTR/Gypsy
AT5G29762	3.37	0.59	5.67	AT5TE40960	ATHILA4A	LTR/Gypsy
AT5G29890	15.03	2.26	6.64	AT5TE41045	ATLANTYS3	LTR/Gypsy
AT5G29975	5.96	0.98	6.10	AT5TE41070	ATHILA6A	LTR/Gypsy
AT5G30189	1.58	0.06	27.09	AT5TE41110	ATLANTYS1	LTR/Gypsy
AT5G30942	1.90	0.00	--	AT5TE41250	ATLANTYS3	LTR/Gypsy
AT5G31087	2.11	0.74	2.84	AT5TE42015	ATHILA3	LTR/Gypsy
AT5G31662	2.32	0.56	4.12	AT5TE41345	ATHILA2	LTR/Gypsy

AT5G31685	2.74	0.00	--	AT5TE42030	ATLANTYS2	LTR/Gypsy
AT5G31719	5.73	0.74	7.77	AT5TE42135	ATHILA6A	LTR/Gypsy
AT5G31752	1.80	0.09	19.93	AT5TE41355	ATLANTYS2	LTR/Gypsy
AT5G31804	8.62	1.19	7.25	AT5TE42190	ATHILA6B	LTR/Gypsy
AT5G31855	1.05	0.33	3.18	AT5TE42265	ATHILA	LTR/Gypsy
AT5G32042	2.09	0.81	2.59	AT5TE41510	ATHILA4	LTR/Gypsy
AT5G32060	2.98	0.62	4.80	AT5TE41675	TAT1_ATH	LTR/Gypsy
AT5G32082	1.51	0.32	4.66	AT5TE41535	ATGP5	LTR/Gypsy
AT5G32107	7.99	1.18	6.75	AT5TE42440	ATHILA6A	LTR/Gypsy
AT5G32197	1.99	0.17	11.39	AT5TE42470	ATHILA6A	LTR/Gypsy
AT5G32228	4.27	0.21	20.64	AT5TE42470	ATHILA6A	LTR/Gypsy
AT5G32306	2.07	0.21	9.99	AT5TE42710	ATHILA6A	LTR/Gypsy
AT5G32345	3.93	0.50	7.80	AT5TE42825	ATHILA2	LTR/Gypsy
AT5G32358	2.92	0.15	19.68	AT5TE42860	ATHILA3	LTR/Gypsy
AT5G32386	2.00	0.17	11.44	AT5TE42870	ATHILA4D_LTR	LTR/Gypsy
AT5G32402	1.21	0.15	8.13	AT5TE42900	ATHILA2	LTR/Gypsy
AT5G32404	2.49	0.36	7.02	AT5TE42900	ATHILA2	LTR/Gypsy
AT5G32475	1.02	0.27	3.85	AT5TE43190	ATHILA2	LTR/Gypsy
AT5G32495	2.11	0.12	18.11	AT5TE43255	ATHILA2	LTR/Gypsy
AT5G32511	2.29	0.15	15.40	AT5TE43260	ATHILA6A	LTR/Gypsy
AT5G32512	1.09	0.00	--	AT5TE43260	ATHILA6A	LTR/Gypsy
AT5G32513	1.51	0.44	3.43	AT5TE43275	ATHILA6A	LTR/Gypsy
AT5G32514	1.94	0.09	21.45	AT5TE43300	ATHILA7	LTR/Gypsy
AT5G32516	1.81	0.17	10.36	AT5TE43315	ATHILA	LTR/Gypsy
AT5G32518	2.92	0.21	14.14	AT5TE43345	ATGP1	LTR/Gypsy
AT5G32520	2.15	0.80	2.69	AT5TE43360	ATLANTYS2	LTR/Gypsy
AT5G32521	2.07	0.45	4.64	AT5TE43315	ATHILA	LTR/Gypsy
AT5G32595	1.54	0.00	--	AT5TE43450	ATHILA6B	LTR/Gypsy
AT5G32598	1.25	0.06	21.49	AT5TE43515	ATLANTYS2	LTR/Gypsy
AT5G32610	2.11	0.56	3.75	AT5TE43540	ATHILA8A	LTR/Gypsy
AT5G32627	1.36	0.59	2.28	AT5TE43650	ATHILA4B_LTR	LTR/Gypsy
AT5G32654	1.53	0.36	4.29	AT5TE43725	ATHILA4A	LTR/Gypsy
AT5G33252	3.79	0.17	21.68	AT5TE44310	ATGP1	LTR/Gypsy
AT5G33306	3.82	0.38	10.00	AT5TE44550	TAT1_ATH	LTR/Gypsy
AT5G33381	1.80	0.63	2.87	AT5TE44680	ATHILA4C	LTR/Gypsy
AT5G33427	1.12	0.15	7.56	AT5TE45005	ATHILA3	LTR/Gypsy
AT5G33990	2.03	0.36	5.72	AT5TE45190	ATHILA4A	LTR/Gypsy
AT5G34082	1.61	0.12	13.86	AT5TE45200	ATHILA4	LTR/Gypsy
AT5G34358	1.30	0.59	2.19	AT5TE45235	ATHILA5	LTR/Gypsy
AT5G34480	2.34	0.98	2.38	AT5TE45925	ATGP8	LTR/Gypsy
AT5G34623	2.34	0.47	4.96	AT5TE46015	ATHILA3	LTR/Gypsy
AT5G34665	3.03	0.54	5.65	AT5TE46060	ATHILA4	LTR/Gypsy
AT5G34686	1.38	0.17	7.90	AT5TE46090	ATLANTYS1	LTR/Gypsy
AT5G34728	2.16	0.62	3.48	AT5TE46100	ATHILA3	LTR/Gypsy
AT5G34770	1.01	0.09	11.18	AT5TE46120	ATHILA	LTR/Gypsy
AT5G34846	1.35	0.27	5.08	AT5TE46355	ATHILA4A	LTR/Gypsy
AT5G34849	12.69	0.86	14.76	AT5TE46515	ATGP1	LTR/Gypsy
AT5G34851	15.83	3.36	4.71	AT5TE46580	ATGP8	LTR/Gypsy
AT5G34855	2.21	0.57	3.89	AT5TE46685	ATHILA4C	LTR/Gypsy
AT5G34862	1.80	0.74	2.42	AT5TE46795	ATHILA4A	LTR/Gypsy
AT5G34985	4.65	0.65	7.13	AT5TE47115	ATHILA6B	LTR/Gypsy
AT5G35057	3.28	1.46	2.25	AT5TE47200	ATHILA2	LTR/Gypsy

AT5G35113	3.85	0.65	5.90	AT5TE47395	ATGP1	LTR/Gypsy
AT5G35602	1.85	0.17	10.60	AT5TE49070	TAT1_ATH	LTR/Gypsy
AT5G38192	12.23	1.51	8.09	AT5TE55145	ATLANTYS2	LTR/Gypsy
AT5G38383	2.07	0.27	7.81	AT5TE55555	ATHILA7	LTR/Gypsy
AT5G38705	5.69	0.36	16.01	AT5TE56030	ATGP1	LTR/Gypsy
AT5G27882	2.61	0.62	4.20	AT5TE36030	RathE1_cons	RathE1_cons
AT1G35930	1.36	0.30	4.56	AT1TE43740	HELITRON1	RC/Helitron
AT1G35940	1.61	0.21	7.79	AT1TE43740	HELITRON1	RC/Helitron
AT1G36140	1.18	0.06	20.33	AT1TE44260	HELITRONY2	RC/Helitron
AT2G04290	1.02	0.36	2.87	AT2TE06880	ATREP11	RC/Helitron
AT2G10460	1.35	0.12	11.58	AT2TE17095	HELITRONY2	RC/Helitron
AT2G11150	0.63	2.13	0.29	AT2TE18495	HELITRON1	RC/Helitron
AT2G11820	1.16	0.24	4.84	AT2TE19730	HELITRONY3	RC/Helitron
AT2G16560	3.21	0.95	3.38	AT2TE29215	ATREP1	RC/Helitron
AT2G19803	3.09	0.50	6.20	AT2TE35745	ATREP4	RC/Helitron
AT2G31520	9.79	2.74	3.58	AT2TE58955	HELITRONY1C	RC/Helitron
AT3G30420	1.44	0.44	3.28	AT3TE50215	HELITRON4	RC/Helitron
AT3G30727	2.35	0.57	4.14	AT3TE51175	ATREP10D	RC/Helitron
AT3G30737	10.31	4.46	2.31	AT3TE51195	HELITRON3	RC/Helitron
AT3G30852	1.59	0.38	4.16	AT3TE51960	ATREP3	RC/Helitron
AT3G31420	1.23	0.41	2.97	AT3TE52570	ATREP3	RC/Helitron
AT3G31440	10.86	2.20	4.93	AT3TE52625	HELITRON1	RC/Helitron
AT3G31442	8.51	2.03	4.20	AT3TE52625	HELITRON1	RC/Helitron
AT3G31904	1.90	0.09	20.98	AT3TE52935	HELITRON2	RC/Helitron
AT3G32043	3.52	1.33	2.65	AT3TE53470	HELITRONY2	RC/Helitron
AT3G42115	1.58	0.06	27.09	AT3TE58680	ATREP5	RC/Helitron
AT3G43350	1.40	0.41	3.38	AT3TE62100	HELITRON2	RC/Helitron
AT3G43425	1.67	0.59	2.80	AT3TE62340	ATREP10B	RC/Helitron
AT4G04230	1.65	0.15	11.07	AT4TE09715	ATREP11A	RC/Helitron
AT4G37570	1.62	0.15	10.91	AT4TE85270	HELITRONY3	RC/Helitron
AT5G27845	2.51	0.45	5.62	AT5TE35925	HELITRONY3	RC/Helitron
AT5G28545	2.84	0.30	9.55	AT5TE38490	ATREP5	RC/Helitron
AT5G34960	2.51	0.53	4.74	AT5TE47085	HELITRON1	RC/Helitron
AT5G35340	2.63	0.32	8.15	AT5TE48170	ATREP11	RC/Helitron
AT5G37080	1.11	0.06	19.02	AT5TE52790	HELITRONY1D	RC/Helitron
AT5G37665	9.61	2.32	4.14	AT5TE53965	HELITRONY1C	RC/Helitron
AT1G03420	13.85	40.12	0.35	AT1TE02770	SADHU	Unassigned
AT1G06740	2.06	8.03	0.26	AT1TE06780	Unassigned	Unassigned
AT1G21260	4.00	12.88	0.31	AT1TE23980	Unassigned	Unassigned
AT1G21290	2.08	0.70	2.94	AT1TE23995	Unassigned	Unassigned
AT1G25430	5.25	0.30	17.65	AT1TE28830	Unassigned	Unassigned
AT1G43250	10.70	3.77	2.84	AT1TE53710	Unassigned	Unassigned
AT1G64240	0.60	1.36	0.44	AT1TE78470	Unassigned	Unassigned
AT1G64250	1.38	4.48	0.31	AT1TE78475	Unassigned	Unassigned
AT1G64270	1.48	30.05	0.05	AT1TE78490	Unassigned	Unassigned
AT1G80020	76.60	169.58	0.45	AT1TE98440	Unassigned	Unassigned
AT2G07737	5.83	0.80	7.28	AT2TE15155	Unassigned	Unassigned
AT2G10130	2.12	0.92	2.29	AT2TE16500	Unassigned	Unassigned
AT2G15720	1.20	0.47	2.54	AT2TE27880	Unassigned	Unassigned
AT2G24760	4.59	9.21	0.50	AT2TE45280	Unassigned	Unassigned
AT2G31080	4.36	0.42	10.39	AT2TE58010	Unassigned	Unassigned
AT3G04605	6.33	26.55	0.24	AT3TE05245	Unassigned	Unassigned

AT3G05850	18.72	38.21	0.49	AT3TE07330	Unassigned	Unassigned
AT3G13438	22.07	72.71	0.30	AT3TE18450	SADHU	Unassigned
AT3G17290	2.57	5.67	0.45	AT3TE24820	Unassigned	Unassigned
AT3G21010	8.82	17.96	0.49	AT3TE30960	Unassigned	Unassigned
AT3G21020	0.91	4.33	0.21	AT3TE30965	Unassigned	Unassigned
AT3G21030	3.26	25.10	0.13	AT3TE30970	Unassigned	Unassigned
AT3G21040	5.96	14.80	0.40	AT3TE30975	Unassigned	Unassigned
AT3G21050	1.52	14.27	0.11	AT3TE30980	Unassigned	Unassigned
AT3G43433	1.65	0.56	2.97	AT3TE62390	Unassigned	Unassigned
AT3G43436	4.50	1.30	3.46	AT3TE62395	Unassigned	Unassigned
AT3G43510	2.35	12.44	0.19	AT3TE62545	Unassigned	Unassigned
AT3G43690	10.48	34.06	0.31	AT3TE63065	Unassigned	Unassigned
AT4G01525	21.43	112.63	0.19	AT4TE03410	SADHU	Unassigned
AT4G08110	11.08	24.60	0.45	AT4TE21115	Unassigned	Unassigned
AT4G18410	1.25	0.21	6.05	AT4TE46305	Unassigned	Unassigned
AT5G07215	2.91	0.51	5.72	AT5TE08220	Unassigned	Unassigned
AT5G16505	7.14	37.37	0.19	AT5TE19520	Unassigned	Unassigned
AT5G27250	0.40	1.21	0.33	AT5TE34840	Unassigned	Unassigned
AT5G27345	2.83	0.72	3.95	AT5TE34980	Unassigned	Unassigned
AT5G27927	1.32	0.41	3.19	AT5TE36235	SADHU	Unassigned
AT5G28626	3.60	12.74	0.28	AT5TE38720	SADHU	Unassigned
AT5G34266	1.44	0.09	15.94	AT5TE45215	Unassigned	Unassigned
AT5G34853	10.19	44.53	0.23	AT5TE46635	Unassigned	Unassigned
AT5G36937	1.04	0.27	3.91	AT5TE52425	Unassigned	Unassigned
AT5G38365	5.44	1.21	4.50	AT5TE55490	Unassigned	Unassigned
AT5G41710	3.54	1.48	2.39	AT5TE60210	Unassigned	Unassigned
AT5G47815	2.68	0.85	3.14	AT5TE69650	Unassigned	Unassigned
AT5G55896	14.43	35.51	0.41	AT5TE81425	Unassigned	Unassigned
