# Supplementary material for: Meiosis-specific gene discovery in plants: RNA-Seq applied to isolated Arabidopsis male meiocytes
Source: BMC Plant Biol. 2010 Dec 17;10:280. doi: 10.1186/1471-2229-10-280 (PMC3018465; doi:10.1186/1471-2229-10-280)
Supplement: Additional file 8 — Table S4. A list of differentially expressed TEs in meiocytes and seedlings. The list of differentially expressed TEs in meiocytes and seedlings, the label of "--" refers to zero (0) reads from seedling. The shaded rows are genes that down-regulated in meiocytes and preferentially expressed in seedlings. M = meiocyte, S = seedling. [file 1471-2229-10-280-S8.PDF]

Supplemental Table S4. A list of differentially expressed TEs in meiocytes and seedlings

Gene	M	S	M/S
AT1G02228	1.21	0.00	--
AT1G03420	13.85	44.61	0.31
AT1G06740	2.06	5.60	0.37
AT1G08105	1.40	0.25	5.66
AT1G08735	3.81	0.16	23.14
AT1G08740	10.87	0.16	66.03
AT1G13660	2.98	0.16	18.13
AT1G15300	16.61	3.29	5.05
AT1G17275	1.63	0.00	--
AT1G17390	5.42	0.08	65.86
AT1G19030	9.88	0.16	60.02
AT1G20390	45.95	114.23	0.40
AT1G21020	9.03	0.16	54.83
AT1G21040	3.81	0.16	23.14
AT1G21290	2.08	0.00	--
AT1G21945	22.40	5.51	4.06
AT1G22560	1.64	0.08	19.91
AT1G23990	3.35	0.00	--
AT1G24640	1.66	0.00	--
AT1G24938	5.45	0.16	33.12
AT1G24967	7.25	0.16	44.07
AT1G25430	5.25	0.41	12.75
AT1G25886	2.00	0.00	--
AT1G26860	1.47	0.08	17.80
AT1G26990	1.31	0.08	15.95
AT1G27780	2.17	0.00	--
AT1G29075	4.80	0.00	--
AT1G29650	3.47	0.00	--
AT1G30030	3.05	0.00	--
AT1G30150	3.70	0.00	--
AT1G30180	2.98	0.00	--
AT1G30340	1.04	0.00	--
AT1G30390	7.78	0.16	47.24
AT1G30980	3.01	0.00	--
AT1G31030	3.86	0.00	--
AT1G31100	3.01	0.00	--
AT1G31210	9.88	0.00	--
AT1G31570	1.73	0.08	21.06
AT1G31993	7.72	0.00	--
AT1G32040	1.66	0.00	--
AT1G32590	2.75	0.00	--
AT1G32890	2.50	0.00	--
AT1G33000	3.85	0.00	--
AT1G33135	1.39	0.00	--
AT1G34530	6.98	0.00	--
AT1G34545	1.00	0.00	--
AT1G34600	1.15	0.00	--
AT1G34620	2.47	0.00	--
AT1G34740	2.37	0.00	--
AT1G34842	0.76	3.62	0.21
AT1G34904	2.83	0.00	--
AT1G34967	4.90	0.00	--
AT1G35060	7.67	0.16	46.58
AT1G35080	2.86	0.00	--
AT1G35090	1.32	0.00	--
AT1G35110	20.44	8.89	2.30
AT1G35115	6.53	32.34	0.20
AT1G35146	2.49	0.00	--
AT1G35186	2.98	0.08	36.27
AT1G35300	1.49	0.00	--
AT1G35390	1.71	0.00	--
AT1G35405	4.30	0.00	--
AT1G35535	1.46	0.00	--
AT1G35590	1.12	0.00	--
AT1G35600	2.73	0.00	--
AT1G35612	3.35	33.50	0.10
AT1G35650	2.26	0.00	--
AT1G35760	9.72	0.00	--
AT1G35770	21.50	0.00	--
AT1G35790	4.45	0.00	--
AT1G35840	1.62	0.00	--
AT1G35930	1.36	0.00	--
AT1G35940	1.61	0.00	--
AT1G35960	1.65	0.00	--
AT1G35970	1.30	0.08	15.83
AT1G35995	1.06	0.08	12.91
AT1G36010	3.23	0.00	--
AT1G36035	3.46	0.00	--
AT1G36040	1.65	0.00	--
AT1G36120	7.07	0.00	--
AT1G36130	3.23	0.08	39.30
AT1G36140	1.18	0.00	--
AT1G36190	1.47	0.00	--
AT1G36200	3.78	0.08	45.95
AT1G36210	2.39	0.00	--
AT1G36270	3.05	0.08	37.12
AT1G36300	2.74	0.00	--
AT1G36305	10.41	0.00	--
AT1G36360	2.35	0.00	--
AT1G36440	4.36	0.00	--
AT1G36460	1.35	0.08	16.35
AT1G36520	8.57	0.08	104.14
AT1G36530	1.07	0.00	--
AT1G36590	3.11	0.00	--
AT1G36600	1.26	0.00	--
AT1G36610	2.34	0.00	--
AT1G36620	1.30	0.00	--
AT1G36630	6.38	0.16	38.75

AT1G36720	4.07	0.00	--
AT1G36770	1.04	0.49	2.10
AT1G36790	1.03	0.00	--
AT1G37015	1.19	0.08	14.50
AT1G37040	1.59	0.00	--
AT1G37050	1.41	0.00	--
AT1G37057	1.23	0.00	--
AT1G37060	2.11	0.00	--
AT1G37170	1.02	0.00	--
AT1G37340	1.21	0.00	--
AT1G37471	2.54	0.08	30.85
AT1G37735	1.32	0.00	--
AT1G37867	1.29	0.00	--
AT1G38185	8.30	0.00	--
AT1G38230	2.21	0.16	13.44
AT1G38300	1.20	0.08	14.58
AT1G38360	2.07	0.08	25.11
AT1G38423	2.07	0.16	12.55
AT1G39110	1.68	0.00	--
AT1G39270	2.93	0.00	--
AT1G39910	1.31	0.00	--
AT1G39990	2.76	0.08	33.53
AT1G40072	2.76	0.08	33.53
AT1G40073	1.31	0.00	--
AT1G40093	2.70	0.00	--
AT1G40095	7.97	0.00	--
AT1G40101	3.37	0.08	40.94
AT1G40105	1.86	0.08	22.62
AT1G40107	1.09	0.00	--
AT1G40109	1.82	0.00	--
AT1G40123	1.03	0.00	--
AT1G41775	3.02	0.08	36.75
AT1G41790	2.35	0.00	--
AT1G41795	3.55	0.00	--
AT1G41797	3.35	0.00	--
AT1G41803	1.48	0.00	--
AT1G41840	1.07	0.00	--
AT1G41930	6.93	3.21	2.16
AT1G42045	1.77	0.00	--
AT1G42050	1.38	0.08	16.80
AT1G42060	1.02	0.00	--
AT1G42110	1.48	0.16	9.01
AT1G42130	2.93	0.08	35.56
AT1G42140	5.29	1.15	4.59
AT1G42150	2.08	0.08	25.33
AT1G42160	2.98	0.16	18.08
AT1G42170	2.23	0.00	--
AT1G42210	2.49	0.00	--
AT1G42320	2.94	0.00	--
AT1G42350	1.50	0.00	--
AT1G42377	1.30	0.16	7.92
AT1G42450	3.18	0.00	--

AT1G42460	2.80	0.00	--
AT1G42500	1.49	0.00	--
AT1G42510	3.15	0.00	--
AT1G42580	0.31	1.40	0.22
AT1G42590	1.80	0.00	--
AT1G42605	5.23	0.00	--
AT1G42620	1.17	0.08	14.17
AT1G42650	2.57	0.00	--
AT1G42695	1.34	0.08	16.24
AT1G42888	2.84	0.00	--
AT1G42924	2.40	0.41	5.82
AT1G43150	2.15	0.00	--
AT1G43200	1.42	0.00	--
AT1G43220	1.05	0.00	--
AT1G43240	1.11	0.00	--
AT1G43250	10.70	0.41	26.01
AT1G43270	3.29	0.00	--
AT1G43280	1.08	0.00	--
AT1G43300	1.89	0.00	--
AT1G43444	2.62	0.00	--
AT1G43570	1.90	0.00	--
AT1G43715	1.33	0.00	--
AT1G43740	1.24	0.00	--
AT1G43775	0.36	2.55	0.14
AT1G43785	1.07	0.00	--
AT1G43830	1.59	0.00	--
AT1G43840	1.50	0.00	--
AT1G43883	2.38	0.00	--
AT1G43960	2.02	0.00	--
AT1G44510	3.40	0.41	8.27
AT1G44840	2.85	0.16	17.30
AT1G44880	1.18	0.00	--
AT1G44935	3.20	0.08	38.83
AT1G45070	2.92	0.00	--
AT1G45090	1.72	0.00	--
AT1G45140	1.10	0.00	--
AT1G46120	1.28	0.16	7.77
AT1G46192	1.58	0.08	19.17
AT1G47565	0.88	2.88	0.30
AT1G47606	1.20	0.00	--
AT1G47650	1.11	0.00	--
AT1G47816	2.76	0.00	--
AT1G47910	2.82	0.25	11.42
AT1G48250	1.14	0.00	--
AT1G48290	3.92	0.00	--
AT1G50810	1.89	8.23	0.23
AT1G51175	9.55	0.00	--
AT1G51750	1.65	0.16	10.01
AT1G52020	1.26	0.00	--
AT1G52087	3.38	0.00	--
AT1G52210	1.54	0.00	--
AT1G52610	1.59	0.00	--

AT1G52850	2.30	0.00	--	AT2G05750	1.49	0.00	--
AT1G55100	5.09	2.47	2.06	AT2G05800	1.06	0.00	--
AT1G55400	1.22	0.00	--	AT2G05930	1.60	0.41	3.88
AT1G56675	2.29	0.00	--	AT2G05935	2.05	0.49	4.15
AT1G57640	3.16	0.25	12.78	AT2G05960	2.89	0.08	35.12
AT1G58020	12.96	0.33	39.37	AT2G06120	2.37	0.00	--
AT1G58889	2.33	6.50	0.36	AT2G06150	1.32	0.00	--
AT1G59265	1.07	5.60	0.19	AT2G06220	1.18	0.00	--
AT1G60020	3.45	0.00	--	AT2G06340	1.96	0.00	--
AT1G62460	3.01	0.49	6.10	AT2G06350	2.81	0.00	--
AT1G62695	6.69	0.00	--	AT2G06400	2.33	0.00	--
AT1G62766	1.36	0.33	4.14	AT2G06440	1.33	0.16	8.08
AT1G64270	1.48	13.58	0.11	AT2G06470	2.29	0.00	--
AT1G65347	2.69	0.25	10.90	AT2G06580	4.29	0.00	--
AT1G65750	1.17	0.00	--	AT2G06590	6.09	0.00	--
AT1G67240	1.97	0.00	--	AT2G06600	7.36	0.00	--
AT1G67626	6.89	0.41	16.75	AT2G06620	1.42	0.00	--
AT1G70010	2.57	0.00	--	AT2G06650	3.52	0.00	--
AT1G73445	1.03	0.41	2.50	AT2G06670	8.67	0.00	--
AT1G78095	2.73	0.00	--	AT2G06680	11.45	0.00	--
AT1G78350	2.81	0.00	--	AT2G06700	2.60	0.00	--
AT2G01022	3.84	0.25	15.55	AT2G06710	1.64	0.00	--
AT2G01026	1.54	0.00	--	AT2G06720	1.58	0.00	--
AT2G01029	3.41	0.16	20.71	AT2G06830	2.69	0.00	--
AT2G01034	1.38	0.00	--	AT2G06840	4.50	0.00	--
AT2G01037	4.42	0.00	--	AT2G06860	25.03	0.33	76.04
AT2G01550	2.20	0.00	--	AT2G06870	4.88	0.00	--
AT2G01840	3.23	0.00	--	AT2G06890	2.95	0.00	--
AT2G02260	1.60	0.00	--	AT2G06930	1.43	0.00	--
AT2G03100	2.15	0.08	26.14	AT2G06950	48.32	17.53	2.76
AT2G03540	0.53	3.79	0.14	AT2G06965	1.31	0.00	--
AT2G03940	1.27	0.00	--	AT2G06967	1.71	0.00	--
AT2G03970	3.25	0.00	--	AT2G06980	2.44	0.00	--
AT2G03990	1.99	0.00	--	AT2G07010	1.79	0.00	--
AT2G04010	1.60	0.00	--	AT2G07100	1.50	0.00	--
AT2G04180	2.74	0.08	33.34	AT2G07150	1.80	0.00	--
AT2G04200	1.73	0.00	--	AT2G07160	5.54	0.00	--
AT2G04210	1.44	0.08	17.50	AT2G07380	3.22	0.00	--
AT2G04290	1.02	0.00	--	AT2G07395	4.93	0.00	--
AT2G04310	1.79	0.08	21.77	AT2G07400	2.95	0.08	35.82
AT2G04330	1.54	0.00	--	AT2G07420	1.98	0.16	12.05
AT2G04670	5.66	0.00	--	AT2G07570	2.35	0.00	--
AT2G04990	1.74	0.00	--	AT2G07580	1.64	0.00	--
AT2G05025	1.97	0.08	23.99	AT2G07650	2.04	0.00	--
AT2G05110	1.71	0.08	20.73	AT2G07682	26.29	0.33	79.87
AT2G05130	1.78	0.00	--	AT2G07683	13.39	1.89	7.07
AT2G05200	1.40	0.00	--	AT2G07685	2.47	0.16	15.00
AT2G05490	2.67	0.00	--	AT2G07686	3.98	0.49	8.07
AT2G05550	1.22	0.00	--	AT2G07693	5.45	0.41	13.25
AT2G05660	2.25	0.00	--	AT2G07694	1.59	0.08	19.28
AT2G05680	4.06	0.00	--	AT2G07697	5.20	0.58	9.02
AT2G05690	1.57	0.00	--	AT2G07703	1.19	0.00	--

AT2G07704	1.43	0.25	5.80
AT2G07729	3.20	0.00	--
AT2G07730	3.33	0.00	--
AT2G07735	1.36	0.08	16.46
AT2G07736	2.06	0.33	6.25
AT2G07737	5.83	0.41	14.17
AT2G07740	2.43	0.00	--
AT2G07767	1.81	0.08	21.99
AT2G07769	1.60	0.16	9.70
AT2G07770	1.49	0.00	--
AT2G07780	2.28	0.00	--
AT2G07784	1.37	0.16	8.34
AT2G07789	4.00	0.00	--
AT2G07791	2.27	0.00	--
AT2G09589	2.82	0.00	--
AT2G09860	1.58	0.00	--
AT2G09910	1.47	0.16	8.96
AT2G09920	2.98	0.00	--
AT2G10070	1.62	0.08	19.69
AT2G10080	1.36	0.00	--
AT2G10100	1.29	0.00	--
AT2G10110	1.22	0.00	--
AT2G10120	1.33	0.00	--
AT2G10130	2.12	0.00	--
AT2G10150	1.04	0.00	--
AT2G10180	1.27	0.00	--
AT2G10250	1.99	0.16	12.09
AT2G10280	19.77	0.00	--
AT2G10290	1.20	0.00	--
AT2G10300	1.25	0.00	--
AT2G10310	2.35	0.00	--
AT2G10320	1.65	0.08	20.10
AT2G10330	6.15	0.00	--
AT2G10350	1.61	0.00	--
AT2G10400	2.22	0.16	13.50
AT2G10405	8.32	2.80	2.97
AT2G10410	73.53	155.88	0.47
AT2G10460	1.35	0.00	--
AT2G10490	1.18	0.00	--
AT2G10540	4.05	0.00	--
AT2G10600	13.04	0.74	17.60
AT2G10620	1.65	0.00	--
AT2G10640	1.04	0.00	--
AT2G10650	2.14	0.16	12.98
AT2G10660	1.55	0.08	18.84
AT2G10670	2.94	0.08	35.75
AT2G10780	1.92	0.00	--
AT2G10860	1.12	0.00	--
AT2G10890	1.20	0.00	--
AT2G10910	1.14	0.16	6.93
AT2G11020	1.05	0.00	--
AT2G11060	1.06	0.00	--

AT2G11140	5.33	2.30	2.31
AT2G11210	1.41	0.00	--
AT2G11220	1.00	0.00	--
AT2G11230	2.65	0.25	10.73
AT2G11240	43.51	214.07	0.20
AT2G11430	7.02	0.00	--
AT2G11450	1.70	0.08	20.62
AT2G11480	1.68	0.00	--
AT2G11550	2.61	0.00	--
AT2G11590	2.31	0.00	--
AT2G11600	1.30	0.00	--
AT2G11700	2.58	0.08	31.29
AT2G11720	1.17	0.00	--
AT2G11770	1.91	0.00	--
AT2G11790	2.16	0.00	--
AT2G11800	2.25	0.00	--
AT2G11820	1.16	0.00	--
AT2G12020	3.41	0.25	13.82
AT2G12060	1.45	0.00	--
AT2G12083	2.69	0.00	--
AT2G12100	1.67	0.00	--
AT2G12110	1.09	0.00	--
AT2G12150	2.79	0.00	--
AT2G12195	3.81	0.00	--
AT2G12210	2.51	0.00	--
AT2G12460	0.17	2.30	0.07
AT2G12570	1.11	0.00	--
AT2G12610	1.89	0.08	22.92
AT2G12680	5.31	0.00	--
AT2G12700	1.28	0.00	--
AT2G12720	2.43	0.00	--
AT2G12730	1.08	0.00	--
AT2G12740	23.66	0.08	287.45
AT2G12750	18.30	0.00	--
AT2G12760	8.79	0.00	--
AT2G12770	3.92	0.00	--
AT2G12800	1.13	0.00	--
AT2G12930	1.87	0.08	22.70
AT2G12970	3.40	0.00	--
AT2G12980	1.30	0.00	--
AT2G13000	3.31	0.16	20.12
AT2G13020	2.24	0.00	--
AT2G13050	1.27	0.00	--
AT2G13080	1.03	0.00	--
AT2G13110	3.04	0.00	--
AT2G13120	2.05	0.00	--
AT2G13160	1.44	0.00	--
AT2G13170	1.67	0.00	--
AT2G13175	1.79	0.00	--
AT2G13260	1.17	0.00	--
AT2G13270	2.16	0.00	--
AT2G13280	1.44	0.00	--

AT2G13300	4.15	0.00	--
AT2G13310	1.31	0.00	--
AT2G13330	1.87	0.00	--
AT2G13380	2.37	0.16	14.37
AT2G13390	2.46	0.08	29.89
AT2G13460	1.75	0.00	--
AT2G13470	1.13	0.00	--
AT2G13520	1.32	0.08	16.06
AT2G13700	1.76	0.00	--
AT2G13740	2.07	0.00	--
AT2G13750	1.11	0.00	--
AT2G13830	3.82	0.08	46.47
AT2G13860	3.94	0.00	--
AT2G13870	2.48	0.00	--
AT2G13940	3.13	0.00	--
AT2G13990	3.60	0.08	43.79
AT2G14030	1.67	0.00	--
AT2G14040	1.43	0.00	--
AT2G14200	1.07	0.00	--
AT2G14220	1.38	3.95	0.35
AT2G14320	1.77	0.08	21.47
AT2G14350	2.99	0.00	--
AT2G14380	1.93	0.00	--
AT2G14400	1.31	0.08	15.95
AT2G14430	1.83	0.00	--
AT2G14590	2.67	0.00	--
AT2G14595	3.42	0.08	41.57
AT2G14640	3.45	0.00	--
AT2G14650	5.09	0.08	61.86
AT2G14770	1.01	0.00	--
AT2G14970	2.45	0.00	--
AT2G14980	1.50	0.00	--
AT2G14990	4.80	0.00	--
AT2G15100	1.43	0.00	--
AT2G15250	2.66	0.08	32.30
AT2G15380	2.27	0.00	--
AT2G15410	8.16	0.00	--
AT2G15510	1.33	0.00	--
AT2G15540	1.26	0.00	--
AT2G15650	2.92	11.28	0.26
AT2G15720	1.20	0.00	--
AT2G15750	1.37	0.08	16.69
AT2G15810	1.20	0.49	2.43
AT2G15920	1.56	0.00	--
AT2G15940	1.59	0.41	3.86
AT2G16000	1.37	0.00	--
AT2G16140	0.36	1.07	0.34
AT2G16150	2.99	0.00	--
AT2G16180	1.55	0.00	--
AT2G16420	3.91	0.00	--
AT2G16560	3.21	0.00	--
AT2G16670	1.29	0.00	--

AT2G16832	2.30	0.00	--
AT2G17460	2.73	0.00	--
AT2G17490	0.45	1.07	0.43
AT2G18820	2.95	0.00	--
AT2G19803	3.09	0.16	18.76
AT2G22210	3.50	0.08	42.50
AT2G22350	1.58	0.00	--
AT2G23480	2.04	0.00	--
AT2G23500	2.56	0.08	31.15
AT2G23880	5.49	1.73	3.18
AT2G24660	1.40	0.49	2.83
AT2G24760	4.59	14.32	0.32
AT2G24890	2.62	0.00	--
AT2G24910	1.23	0.00	--
AT2G24930	2.98	0.00	--
AT2G25550	10.31	0.00	--
AT2G26630	1.49	0.25	6.04
AT2G28750	1.89	0.00	--
AT2G28980	1.86	0.00	--
AT2G29165	1.06	0.16	6.41
AT2G29230	4.03	0.08	48.98
AT2G29240	10.58	0.16	64.27
AT2G30640	4.54	1.32	3.44
AT2G31080	4.36	0.16	26.50
AT2G31520	9.79	0.74	13.22
AT2G34130	1.74	0.00	--
AT2G36402	1.31	0.08	15.95
AT2G41745	2.55	0.08	30.96
AT2G42050	1.08	0.00	--
AT2G45230	7.11	0.08	86.37
AT3G04605	6.33	26.58	0.24
AT3G09165	2.47	0.00	--
AT3G10100	8.20	1.07	7.67
AT3G11970	3.21	0.00	--
AT3G14800	24.65	11.36	2.17
AT3G15602	1.22	0.00	--
AT3G17050	20.73	264.02	0.08
AT3G17260	15.15	0.08	184.04
AT3G17290	2.57	0.16	15.63
AT3G20975	43.54	0.25	176.35
AT3G20990	9.20	0.00	--
AT3G21010	8.82	2.06	4.29
AT3G21030	3.26	0.66	4.95
AT3G21040	5.96	0.16	36.19
AT3G23725	1.61	0.16	9.79
AT3G24390	2.05	0.00	--
AT3G24675	1.54	0.00	--
AT3G25815	2.38	0.00	--
AT3G26265	3.23	0.00	--
AT3G26525	3.81	0.16	23.14
AT3G26530	10.87	0.16	66.03
AT3G26614	1.45	0.25	5.87

AT3G28160	5.33	14.98	0.36
AT3G28315	3.78	0.00	--
AT3G28705	1.84	0.08	22.40
AT3G28915	5.28	0.74	7.13
AT3G29032	3.26	0.00	--
AT3G29076	3.29	0.00	--
AT3G29078	1.34	0.00	--
AT3G29153	1.32	0.00	--
AT3G29156	8.01	0.16	48.69
AT3G29480	3.93	0.00	--
AT3G29510	3.68	0.00	--
AT3G29612	1.53	0.00	--
AT3G29615	2.33	0.00	--
AT3G29618	1.12	0.00	--
AT3G29641	2.38	0.00	--
AT3G29642	3.79	0.58	6.58
AT3G29695	2.44	0.08	29.70
AT3G29710	2.73	0.00	--
AT3G29777	2.47	0.00	--
AT3G29778	2.18	0.00	--
AT3G30170	2.21	0.00	--
AT3G30183	1.29	0.00	--
AT3G30190	1.32	0.08	16.06
AT3G30330	1.15	0.00	--
AT3G30396	1.33	0.08	16.13
AT3G30400	1.78	0.00	--
AT3G30420	1.44	0.00	--
AT3G30433	1.30	0.00	--
AT3G30436	1.31	0.00	--
AT3G30465	3.75	0.00	--
AT3G30490	1.10	0.00	--
AT3G30570	2.14	0.00	--
AT3G30582	1.11	0.00	--
AT3G30655	1.72	0.00	--
AT3G30670	1.47	0.00	--
AT3G30680	1.07	0.08	13.02
AT3G30695	24.74	0.08	300.56
AT3G30703	10.05	0.00	--
AT3G30721	1.40	0.00	--
AT3G30722	1.00	0.00	--
AT3G30727	2.35	0.00	--
AT3G30737	10.31	1.56	6.59
AT3G30742	1.00	0.00	--
AT3G30743	2.01	0.08	24.40
AT3G30746	1.47	0.00	--
AT3G30749	1.90	0.00	--
AT3G30763	1.79	0.00	--
AT3G30780	1.32	0.00	--
AT3G30790	4.85	0.08	58.92
AT3G30802	6.77	0.00	--
AT3G30803	1.12	0.00	--
AT3G30810	2.85	0.00	--

AT3G30811	1.62	0.00	--
AT3G30819	1.56	0.08	18.95
AT3G30821	1.30	0.00	--
AT3G30825	1.55	0.00	--
AT3G30836	1.68	0.00	--
AT3G30837	1.95	0.00	--
AT3G30838	1.41	0.00	--
AT3G30846	5.07	0.00	--
AT3G30852	1.59	0.00	--
AT3G31023	2.86	0.00	--
AT3G31310	1.17	0.00	--
AT3G31317	2.29	0.00	--
AT3G31410	1.03	0.00	--
AT3G31420	1.23	0.00	--
AT3G31440	10.86	0.66	16.49
AT3G31442	8.51	0.58	14.78
AT3G31450	3.38	0.16	20.53
AT3G31490	1.10	0.00	--
AT3G31500	1.36	0.00	--
AT3G31630	2.93	0.08	35.64
AT3G31904	1.90	0.00	--
AT3G31915	1.40	0.00	--
AT3G31920	1.03	0.08	12.50
AT3G31935	2.06	0.00	--
AT3G31945	1.62	0.00	--
AT3G31970	5.00	0.08	60.71
AT3G32032	4.74	0.00	--
AT3G32033	1.03	0.00	--
AT3G32043	3.52	0.16	21.36
AT3G32060	1.37	0.00	--
AT3G32080	1.42	0.00	--
AT3G32092	2.95	0.82	3.58
AT3G32110	2.67	0.08	32.49
AT3G32118	1.08	0.00	--
AT3G32164	1.73	0.00	--
AT3G32168	1.58	0.00	--
AT3G32195	1.03	0.00	--
AT3G32210	8.92	0.00	--
AT3G32226	1.54	0.08	18.65
AT3G32230	1.60	0.00	--
AT3G32383	1.38	0.00	--
AT3G32393	1.15	0.00	--
AT3G32425	2.73	0.00	--
AT3G32475	1.35	0.00	--
AT3G32677	1.13	0.00	--
AT3G32894	2.06	0.00	--
AT3G32914	2.79	0.00	--
AT3G32917	3.36	0.00	--
AT3G32925	2.18	0.33	6.62
AT3G32950	2.37	0.00	--
AT3G32966	2.57	0.00	--
AT3G32968	1.88	0.00	--

AT3G32970	2.55	0.00	--	AT3G42650	1.36	0.00	--
AT3G32975	1.34	0.00	--	AT3G42658	4.58	1.40	3.28
AT3G33005	2.08	0.00	--	AT3G42690	1.40	0.00	--
AT3G33006	1.49	0.00	--	AT3G42720	1.01	0.00	--
AT3G33009	1.48	0.00	--	AT3G42794	1.03	0.08	12.50
AT3G33058	3.18	0.00	--	AT3G42803	2.15	0.00	--
AT3G33069	2.52	0.08	30.63	AT3G42806	9.24	3.87	2.39
AT3G33076	8.14	0.16	49.45	AT3G42820	18.24	0.33	55.40
AT3G33080	1.93	0.00	--	AT3G42836	1.22	0.00	--
AT3G33084	8.40	0.16	51.06	AT3G42837	1.78	0.00	--
AT3G33091	4.40	0.00	--	AT3G42900	1.04	0.00	--
AT3G33097	1.22	0.00	--	AT3G42910	1.83	0.16	11.14
AT3G33100	3.27	0.00	--	AT3G42935	1.13	0.08	13.76
AT3G33106	2.37	0.00	--	AT3G42945	3.81	0.00	--
AT3G33109	1.16	0.00	--	AT3G42993	1.20	0.00	--
AT3G33115	1.10	0.00	--	AT3G42996	1.81	0.00	--
AT3G33133	2.26	0.08	27.40	AT3G43080	1.72	0.00	--
AT3G33136	1.65	0.00	--	AT3G43090	1.13	0.00	--
AT3G33175	1.18	0.00	--	AT3G43144	1.83	0.16	11.11
AT3G33178	1.14	0.00	--	AT3G43151	1.67	0.00	--
AT3G33225	1.50	0.00	--	AT3G43154	2.88	0.00	--
AT3G33235	2.91	0.08	35.42	AT3G43156	1.90	0.00	--
AT3G33448	1.64	0.08	19.91	AT3G43157	1.16	0.00	--
AT3G33537	4.45	0.25	18.03	AT3G43175	1.90	0.08	23.03
AT3G33555	3.09	0.00	--	AT3G43302	2.14	0.00	--
AT3G33565	1.58	0.00	--	AT3G43304	2.01	0.00	--
AT3G33595	1.43	0.00	--	AT3G43307	5.08	0.00	--
AT3G39230	2.23	0.00	--	AT3G43315	1.40	0.08	16.98
AT3G39935	1.03	0.00	--	AT3G43350	1.40	0.16	8.49
AT3G41345	1.23	0.00	--	AT3G43357	1.57	0.00	--
AT3G42057	6.66	0.00	--	AT3G43360	1.35	0.00	--
AT3G42070	1.17	0.00	--	AT3G43370	1.32	0.00	--
AT3G42115	1.58	0.00	--	AT3G43390	1.53	0.00	--
AT3G42120	1.26	0.00	--	AT3G43425	1.67	0.00	--
AT3G42252	3.25	0.00	--	AT3G43433	1.65	0.00	--
AT3G42256	1.35	0.00	--	AT3G43436	4.50	0.00	--
AT3G42257	1.54	0.08	18.65	AT3G43510	2.35	5.68	0.41
AT3G42270	1.54	0.00	--	AT3G43526	1.16	0.08	14.06
AT3G42290	2.12	0.00	--	AT3G43530	4.75	0.08	57.74
AT3G42300	1.50	0.00	--	AT3G43546	2.00	0.00	--
AT3G42305	2.72	0.08	33.04	AT3G43566	1.87	0.08	22.73
AT3G42313	10.28	0.00	--	AT3G43573	1.64	0.00	--
AT3G42430	1.49	0.00	--	AT3G43575	1.36	0.08	16.46
AT3G42431	1.10	0.00	--	AT3G43625	2.81	0.08	34.19
AT3G42436	1.38	0.00	--	AT3G43635	1.41	0.00	--
AT3G42445	1.13	0.00	--	AT3G43640	1.10	0.00	--
AT3G42478	1.82	0.08	22.10	AT3G43654	4.37	0.41	10.62
AT3G42530	1.00	0.00	--	AT3G43675	1.00	0.08	12.17
AT3G42545	8.37	0.00	--	AT3G43680	3.81	0.00	--
AT3G42553	2.04	0.08	24.81	AT3G43681	4.39	0.00	--
AT3G42626	1.58	0.00	--	AT3G43688	1.17	0.00	--
AT3G42645	3.87	0.00	--	AT3G43730	1.55	0.00	--

AT3G43760	1.09	0.00	--
AT3G43780	2.16	0.00	--
AT3G43825	3.52	0.08	42.83
AT3G43830	2.11	0.00	--
AT3G43835	2.84	0.16	17.24
AT3G43862	2.53	0.00	--
AT3G43863	6.65	0.00	--
AT3G43864	1.00	0.00	--
AT3G43955	4.83	12.02	0.40
AT3G44035	1.53	0.00	--
AT3G44042	2.26	0.08	27.48
AT3G44093	1.84	0.00	--
AT3G44096	1.93	0.82	2.34
AT3G44175	2.15	0.08	26.14
AT3G44215	1.16	0.00	--
AT3G44325	3.03	0.00	--
AT3G44425	2.89	0.08	35.12
AT3G44470	1.13	0.00	--
AT3G44640	1.02	0.00	--
AT3G44650	1.90	0.00	--
AT3G44705	4.18	0.00	--
AT3G44796	13.58	0.00	--
AT3G45095	2.35	0.16	14.28
AT3G45253	24.13	0.08	293.21
AT3G45256	1.21	0.00	--
AT3G45270	1.16	0.08	14.06
AT3G45340	1.18	0.08	14.39
AT3G45380	1.95	0.00	--
AT3G45520	1.65	0.00	--
AT3G45550	3.50	0.00	--
AT3G46487	1.09	0.00	--
AT3G47270	1.08	0.00	--
AT3G47875	1.14	0.33	3.47
AT3G50625	1.96	0.00	--
AT3G54823	3.00	0.16	18.23
AT3G57586	1.84	0.16	11.20
AT3G58865	16.63	4.12	4.04
AT3G59860	1.73	0.00	--
AT3G60565	13.63	0.00	--
AT3G60935	1.07	0.00	--
AT3G60965	4.72	9.79	0.48
AT3G62475	3.60	0.00	--
AT3G62480	1.07	0.08	13.02
AT3G62490	4.14	0.99	4.19
AT3G62520	1.44	0.00	--
AT4G01525	21.43	1.98	10.85
AT4G02314	2.02	0.00	--
AT4G02960	1.63	0.08	19.80
AT4G03310	1.74	0.00	--
AT4G03650	2.33	0.00	--
AT4G03760	1.42	0.00	--
AT4G03770	3.30	0.00	--

AT4G03790	1.86	0.00	--
AT4G03816	3.15	0.49	6.38
AT4G03840	1.36	0.00	--
AT4G03860	1.25	0.00	--
AT4G03910	1.62	0.00	--
AT4G03920	1.48	0.00	--
AT4G03970	1.12	0.08	13.57
AT4G03981	2.12	0.00	--
AT4G04000	2.02	0.00	--
AT4G04050	1.57	0.00	--
AT4G04070	1.00	0.00	--
AT4G04130	2.55	0.58	4.43
AT4G04157	1.44	0.00	--
AT4G04165	2.04	0.00	--
AT4G04170	1.80	0.00	--
AT4G04230	1.65	0.00	--
AT4G04270	1.65	0.00	--
AT4G04310	1.08	0.00	--
AT4G04393	1.05	0.08	12.72
AT4G04400	1.58	0.00	--
AT4G04410	0.50	64.19	0.01
AT4G04430	1.16	0.16	7.03
AT4G04530	1.84	0.00	--
AT4G04635	1.17	0.16	7.14
AT4G05073	1.04	0.00	--
AT4G05133	2.10	0.08	25.51
AT4G05145	1.57	0.00	--
AT4G05280	1.19	0.00	--
AT4G05570	1.33	0.16	8.08
AT4G05585	3.06	0.00	--
AT4G05613	1.00	0.00	--
AT4G05635	1.54	0.00	--
AT4G06477	47.00	0.91	51.92
AT4G06485	3.41	0.08	41.38
AT4G06499	1.15	0.08	13.98
AT4G06506	1.79	0.00	--
AT4G06509	3.41	0.08	41.46
AT4G06510	1.87	0.00	--
AT4G06516	1.40	0.00	--
AT4G06517	2.23	0.00	--
AT4G06518	1.39	0.00	--
AT4G06529	1.18	0.00	--
AT4G06556	1.15	0.08	13.95
AT4G06579	2.22	0.16	13.50
AT4G06584	1.36	0.08	16.46
AT4G06609	1.14	0.00	--
AT4G06656	25.52	0.00	--
AT4G06660	4.57	0.00	--
AT4G06664	14.55	0.00	--
AT4G06666	4.70	0.00	--
AT4G06670	1.09	0.00	--
AT4G06704	1.95	0.00	--

AT4G06712	20.75	0.00	--
AT4G06714	8.34	0.00	--
AT4G06724	1.12	0.00	--
AT4G06726	2.74	0.00	--
AT4G06728	1.36	0.00	--
AT4G06734	1.35	0.00	--
AT4G06736	1.17	0.00	--
AT4G06752	1.68	0.00	--
AT4G07315	1.42	0.00	--
AT4G07334	1.83	0.00	--
AT4G07339	1.20	0.00	--
AT4G07454	1.69	0.00	--
AT4G07456	2.61	0.00	--
AT4G07458	4.73	0.00	--
AT4G07460	1.84	0.00	--
AT4G07498	1.00	0.00	--
AT4G07500	1.23	0.00	--
AT4G07502	2.15	0.00	--
AT4G07504	1.02	0.00	--
AT4G07507	1.83	5.76	0.32
AT4G07516	5.63	0.00	--
AT4G07518	1.72	0.00	--
AT4G07520	1.45	0.00	--
AT4G07528	1.12	0.00	--
AT4G07600	1.07	0.00	--
AT4G07664	1.60	0.00	--
AT4G07668	1.51	0.00	--
AT4G07680	6.02	0.00	--
AT4G07688	1.24	0.00	--
AT4G07690	1.25	0.00	--
AT4G07693	2.48	0.00	--
AT4G07696	2.17	0.08	26.37
AT4G07700	2.08	0.00	--
AT4G07725	1.66	0.00	--
AT4G07733	4.18	0.00	--
AT4G07738	3.71	0.16	22.51
AT4G07742	1.29	0.00	--
AT4G07750	1.17	0.00	--
AT4G07760	2.03	0.00	--
AT4G07770	1.63	0.00	--
AT4G07830	1.11	0.41	2.69
AT4G07850	1.25	0.08	15.20
AT4G07856	2.71	0.00	--
AT4G07890	1.88	0.00	--
AT4G07893	2.59	0.00	--
AT4G07920	1.11	0.08	13.54
AT4G07933	4.88	0.00	--
AT4G07934	2.21	0.00	--
AT4G07935	1.69	0.00	--
AT4G07942	3.39	0.08	41.24
AT4G08030	1.21	0.16	7.34
AT4G08050	4.26	0.08	51.77

AT4G08053	3.65	0.16	22.19
AT4G08054	1.03	0.16	6.25
AT4G08060	2.24	0.00	--
AT4G08070	1.08	0.00	--
AT4G08078	1.36	0.00	--
AT4G08080	1.92	0.00	--
AT4G08096	1.00	0.00	--
AT4G08099	1.24	0.00	--
AT4G08100	0.27	3.21	0.08
AT4G08101	1.35	0.00	--
AT4G08103	1.54	0.00	--
AT4G08105	5.09	0.00	--
AT4G08110	11.08	24.61	0.45
AT4G08114	1.29	0.08	15.72
AT4G08115	0.36	1.81	0.20
AT4G08120	1.22	0.00	--
AT4G08130	1.13	0.00	--
AT4G08131	1.20	0.00	--
AT4G08138	4.90	0.00	--
AT4G08220	2.01	8.31	0.24
AT4G08262	8.13	0.00	--
AT4G08333	1.41	0.00	--
AT4G08340	25.84	0.58	44.86
AT4G08490	2.32	0.00	--
AT4G08600	1.33	0.00	--
AT4G08650	2.08	0.08	25.33
AT4G08660	2.39	0.00	--
AT4G08680	2.73	0.16	16.61
AT4G08710	1.64	0.08	19.91
AT4G08720	2.33	0.00	--
AT4G08820	1.53	0.00	--
AT4G08830	2.12	0.00	--
AT4G08860	2.55	0.00	--
AT4G08880	2.11	0.08	25.63
AT4G08890	1.06	0.00	--
AT4G08970	2.97	0.00	--
AT4G08995	6.50	0.00	--
AT4G09146	2.12	0.00	--
AT4G09205	10.55	0.66	16.02
AT4G09230	6.68	0.00	--
AT4G09255	10.73	0.66	16.30
AT4G09280	6.59	0.00	--
AT4G09290	1.90	0.08	23.14
AT4G09313	4.45	0.16	27.03
AT4G09316	1.11	0.00	--
AT4G09380	1.01	0.16	6.14
AT4G09410	1.13	0.00	--
AT4G09455	3.55	0.66	5.40
AT4G09480	1.13	0.16	6.88
AT4G09710	2.21	0.00	--
AT4G10460	1.15	0.00	--
AT4G10580	3.72	0.41	9.03

AT4G10830	4.25	0.00	--
AT4G10865	1.04	0.00	--
AT4G11200	5.33	0.74	7.19
AT4G11375	2.62	0.00	--
AT4G11710	4.02	0.08	48.87
AT4G12423	5.33	0.00	--
AT4G13120	1.58	5.93	0.27
AT4G13470	3.38	0.00	--
AT4G15590	6.50	0.00	--
AT4G16870	3.11	65.02	0.05
AT4G16910	0.45	2.30	0.20
AT4G18410	1.25	0.00	--
AT4G19280	2.64	0.00	--
AT4G19300	1.46	0.00	--
AT4G19310	5.32	0.00	--
AT4G19320	4.65	0.00	--
AT4G20365	1.48	0.00	--
AT4G20490	2.66	0.08	32.30
AT4G20500	1.89	0.08	22.92
AT4G20510	5.94	0.33	18.05
AT4G20725	8.68	0.33	26.38
AT4G20730	8.95	1.07	8.37
AT4G21420	0.46	1.56	0.30
AT4G22040	3.29	0.25	13.34
AT4G22415	1.03	0.00	--
AT4G26360	3.78	0.08	45.98
AT4G27200	2.25	0.00	--
AT4G27210	3.28	0.00	--
AT4G27597	1.44	0.00	--
AT4G32205	1.44	0.08	17.50
AT4G37570	1.62	0.08	19.69
AT5G01185	4.42	0.00	--
AT5G01335	4.53	0.74	6.11
AT5G03950	1.84	0.00	--
AT5G06805	1.19	0.00	--
AT5G07215	2.91	0.00	--
AT5G07505	4.85	0.00	--
AT5G12085	1.98	0.00	--
AT5G13475	2.23	0.08	27.07
AT5G14810	1.54	0.33	4.67
AT5G14830	7.23	2.06	3.51
AT5G16505	7.14	31.69	0.23
AT5G17125	4.54	0.00	--
AT5G17725	1.76	0.25	7.12
AT5G18633	1.79	0.25	7.23
AT5G20750	1.85	0.00	--
AT5G23955	3.31	9.22	0.36
AT5G24915	6.21	0.08	75.43
AT5G25045	3.11	0.41	7.56
AT5G25205	2.36	0.00	--
AT5G25615	4.91	0.00	--
AT5G26236	16.35	0.08	198.66
AT5G26283	1.76	0.00	--
AT5G26345	2.74	0.00	--
AT5G26350	1.59	0.08	19.28
AT5G26618	1.10	0.08	13.35
AT5G26775	6.30	0.00	--
AT5G27160	2.20	0.00	--
AT5G27190	1.86	0.00	--
AT5G27250	0.40	2.55	0.16
AT5G27345	2.83	0.00	--
AT5G27500	1.22	0.00	--
AT5G27505	3.66	0.00	--
AT5G27590	2.44	0.00	--
AT5G27845	2.51	0.08	30.44
AT5G27882	2.61	0.08	31.67
AT5G27885	5.10	0.00	--
AT5G27895	0.82	1.98	0.42
AT5G27902	1.04	0.25	4.20
AT5G27905	2.84	0.00	--
AT5G27927	1.32	0.16	8.03
AT5G27965	1.39	0.00	--
AT5G28053	2.92	0.82	3.54
AT5G28165	2.46	0.00	--
AT5G28170	1.88	0.08	22.81
AT5G28173	7.60	0.16	46.17
AT5G28200	2.90	0.00	--
AT5G28232	1.50	0.08	18.21
AT5G28250	1.78	0.00	--
AT5G28253	1.09	0.00	--
AT5G28263	6.75	2.14	3.16
AT5G28285	1.61	0.08	19.50
AT5G28335	6.82	0.00	--
AT5G28405	1.15	0.00	--
AT5G28468	1.00	0.00	--
AT5G28480	2.21	0.00	--
AT5G28484	1.08	0.00	--
AT5G28487	3.74	0.00	--
AT5G28495	3.08	0.00	--
AT5G28523	7.81	0.00	--
AT5G28526	3.01	0.08	36.56
AT5G28545	2.84	0.00	--
AT5G28593	1.86	0.08	22.59
AT5G28596	2.08	0.00	--
AT5G28600	22.87	0.08	277.93
AT5G28605	3.62	0.00	--
AT5G28622	3.46	1.40	2.47
AT5G28624	1.14	0.00	--
AT5G28635	1.18	0.00	--
AT5G28641	1.23	0.00	--
AT5G28692	1.63	0.00	--
AT5G28696	1.81	0.00	--
AT5G28715	9.37	0.00	--
AT5G28760	1.15	0.00	--

AT5G28773	5.46	0.00	--
AT5G28776	5.42	0.00	--
AT5G28785	1.68	0.00	--
AT5G28865	2.05	0.00	--
AT5G28870	1.35	0.00	--
AT5G28923	1.28	0.00	--
AT5G28970	10.00	0.00	--
AT5G28980	1.60	0.00	--
AT5G28993	6.53	0.16	39.70
AT5G29015	4.21	0.00	--
AT5G29020	1.66	0.00	--
AT5G29043	1.36	0.00	--
AT5G29046	1.72	0.00	--
AT5G29053	1.55	0.00	--
AT5G29075	3.63	0.25	14.69
AT5G29408	1.06	0.00	--
AT5G29646	1.18	0.00	--
AT5G29720	2.16	0.00	--
AT5G29762	3.37	0.08	40.94
AT5G29890	15.03	0.00	--
AT5G29975	5.96	0.00	--
AT5G30189	1.58	0.00	--
AT5G30269	4.20	0.00	--
AT5G30545	1.03	0.00	--
AT5G30584	1.33	0.08	16.13
AT5G30942	1.90	0.00	--
AT5G31087	2.11	0.08	25.63
AT5G31662	2.32	0.00	--
AT5G31685	2.74	0.00	--
AT5G31719	5.73	0.00	--
AT5G31752	1.80	0.00	--
AT5G31804	8.62	0.00	--
AT5G31845	1.46	0.08	17.72
AT5G31855	1.05	0.00	--
AT5G31891	1.03	0.00	--
AT5G31962	1.09	0.00	--
AT5G32042	2.09	0.08	25.44
AT5G32060	2.98	0.00	--
AT5G32082	1.51	0.08	18.32
AT5G32107	7.99	0.00	--
AT5G32197	1.99	0.00	--
AT5G32228	4.27	0.00	--
AT5G32306	2.07	0.00	--
AT5G32345	3.93	0.25	15.92
AT5G32358	2.92	0.00	--
AT5G32386	2.00	0.00	--
AT5G32402	1.21	0.00	--
AT5G32404	2.49	0.00	--
AT5G32475	1.02	0.00	--
AT5G32484	2.99	0.00	--
AT5G32487	2.19	0.00	--
AT5G32495	2.11	0.00	--

AT5G32511	2.29	0.00	--
AT5G32512	1.09	0.00	--
AT5G32513	1.51	0.00	--
AT5G32514	1.94	0.00	--
AT5G32516	1.81	0.00	--
AT5G32518	2.92	0.00	--
AT5G32520	2.15	0.16	13.07
AT5G32521	2.07	0.08	25.11
AT5G32595	1.54	0.00	--
AT5G32598	1.25	0.00	--
AT5G32610	2.11	0.00	--
AT5G32616	1.27	0.00	--
AT5G32621	1.08	0.16	6.56
AT5G32627	1.36	0.25	5.49
AT5G32654	1.53	0.08	18.54
AT5G32702	1.26	0.08	15.32
AT5G33220	1.54	0.16	9.38
AT5G33234	3.53	0.08	42.94
AT5G33252	3.79	0.00	--
AT5G33306	3.82	0.00	--
AT5G33360	5.68	0.00	--
AT5G33381	1.80	0.00	--
AT5G33382	2.37	0.66	3.60
AT5G33402	2.18	0.08	26.48
AT5G33427	1.12	0.00	--
AT5G33990	2.03	0.08	24.70
AT5G34082	1.61	0.00	--
AT5G34266	1.44	0.00	--
AT5G34358	1.30	0.00	--
AT5G34412	2.07	0.00	--
AT5G34480	2.34	0.00	--
AT5G34623	2.34	0.00	--
AT5G34665	3.03	0.00	--
AT5G34686	1.38	0.00	--
AT5G34728	2.16	0.08	26.25
AT5G34770	1.01	0.25	4.09
AT5G34800	0.59	1.48	0.40
AT5G34846	1.35	0.00	--
AT5G34849	12.69	0.00	--
AT5G34851	15.83	1.98	8.01
AT5G34853	10.19	27.82	0.37
AT5G34855	2.21	0.00	--
AT5G34856	1.17	0.00	--
AT5G34862	1.80	0.08	21.88
AT5G34960	2.51	0.58	4.36
AT5G34985	4.65	0.00	--
AT5G35025	2.89	0.00	--
AT5G35035	1.05	0.00	--
AT5G35045	1.34	0.08	16.24
AT5G35057	3.28	0.00	--
AT5G35076	3.65	0.00	--
AT5G35113	3.85	0.00	--

AT5G35116	1.07	0.00	--
AT5G35205	6.40	0.00	--
AT5G35331	1.95	0.08	23.66
AT5G35340	2.63	0.00	--
AT5G35413	3.27	0.00	--
AT5G35416	4.02	0.08	48.84
AT5G35495	1.48	0.00	--
AT5G35535	3.74	0.00	--
AT5G35575	1.13	0.25	4.59
AT5G35602	1.85	0.00	--
AT5G35606	1.37	0.00	--
AT5G35643	1.17	0.08	14.28
AT5G35720	2.53	0.00	--
AT5G35725	7.34	0.00	--
AT5G35756	1.76	0.00	--
AT5G35791	1.70	0.08	20.62
AT5G35794	3.67	0.00	--
AT5G35802	1.10	0.00	--
AT5G35820	2.53	0.08	30.74
AT5G35914	0.41	1.89	0.22
AT5G35935	2.17	4.53	0.48
AT5G36005	1.22	0.00	--
AT5G36020	4.50	0.00	--
AT5G36030	4.63	0.00	--
AT5G36040	1.04	0.00	--
AT5G36050	1.47	0.00	--
AT5G36060	1.02	0.00	--
AT5G36070	1.39	0.00	--
AT5G36075	7.48	0.08	90.85
AT5G36650	1.30	0.00	--
AT5G36655	1.00	0.00	--
AT5G36860	2.13	0.00	--
AT5G36905	6.47	0.00	--
AT5G36935	10.10	0.08	122.71
AT5G36937	1.04	0.33	3.15
AT5G37080	1.11	0.00	--
AT5G37145	1.03	0.00	--
AT5G37330	1.09	0.00	--
AT5G37665	9.61	0.00	--
AT5G38035	3.45	0.00	--
AT5G38192	12.23	0.00	--
AT5G38285	4.90	0.16	29.80
AT5G38365	5.44	0.00	--
AT5G38383	2.07	0.00	--
AT5G38437	1.00	0.00	--
AT5G38705	5.69	0.08	69.16
AT5G38870	8.03	30.37	0.26
AT5G39060	1.14	0.33	3.47
AT5G39095	1.81	0.00	--
AT5G39155	11.39	0.00	--
AT5G39185	11.34	0.00	--
AT5G39245	2.78	0.00	--

AT5G39862	3.12	0.00	--
AT5G40110	1.53	0.00	--
AT5G40605	3.23	0.08	39.27
AT5G41710	3.54	0.25	14.34
AT5G41755	1.07	0.00	--
AT5G41835	1.71	0.16	10.36
AT5G43015	3.51	0.16	21.32
AT5G43065	9.09	0.00	--
AT5G43105	13.30	0.00	--
AT5G43415	3.37	0.00	--
AT5G43800	6.42	0.16	38.97
AT5G44875	3.81	0.16	23.14
AT5G44890	10.87	0.16	66.03
AT5G45082	2.00	0.00	--
AT5G45116	2.14	0.00	--
AT5G45576	1.33	0.08	16.13
AT5G45605	2.80	0.00	--
AT5G46645	2.27	0.00	--
AT5G46665	2.16	0.91	2.39
AT5G47445	6.27	1.15	5.44
AT5G47815	2.68	0.08	32.60
AT5G49465	1.29	0.00	--
AT5G52055	3.06	0.00	--
AT5G52065	1.80	0.00	--
AT5G53775	7.42	0.08	90.15
AT5G53815	3.37	0.08	40.94
AT5G54203	3.43	0.25	13.89
AT5G55875	2.29	0.00	--
AT5G55896	14.43	39.09	0.37
AT5G56367	3.49	0.16	21.21
AT5G57126	1.05	0.00	--
AT5G59640	3.38	0.08	41.05
AT5G64685	8.35	1.81	4.61
